# A pre‐Miocene Irano‐Turanian cradle: Origin and diversification of the species‐rich monocot genus *Gagea* (Liliaceae)

**DOI:** 10.1002/ece3.5170

**Published:** 2019-04-26

**Authors:** Angela Peterson, Dörte Harpke, Jens Peterson, Alexander Harpke, Lorenzo Peruzzi

**Affiliations:** ^1^ Institute of Biology Martin‐Luther‐University of Halle‐Wittenberg Halle/Saale Germany; ^2^ Leibniz Institute of Plant Genetics and Crop Plant Research (IPK) Gatersleben Germany; ^3^ State Office for Environmental Protection of Saxony‐Anhalt Halle/Saale Germany; ^4^ Department of Community Ecology Helmholtz Centre for Environmental Research (UFZ) Halle Germany; ^5^ Department of Biology, Unit of Botany University of Pisa Pisa Italy

**Keywords:** ancestral area reconstruction, biogeography, dated phylogeny, *Gagea*, Irano‐Turanian region, southwestern Asia

## Abstract

The Irano‐Turanian (IT) floristic region is considered an important center of origin for many taxa. However, there is a lack of studies dealing with typical IT genera that also occur in neighboring areas. The species‐rich monocot genus *Gagea* Salisb. shows a center of diversity in IT region and a distribution in adjacent regions, therefore representing a good study object to investigate spatial and temporal relationships among IT region and its neighboring areas (East Asia, Euro‐Siberia, Himalaya, and Mediterranean). We aimed at (a) testing the origin of the genus and of its major lineages in the IT region, (b) reconstructing divergence times, and (c) reconstructing colonization events. To address these problems, sequences of the ribosomal DNA internal transcribed spacer (ITS) region of 418 individuals and chloroplast intergenic spacers sequences (*psb*A‐*trn*H, *trn*L‐*trn*F) of 497 individuals, representing 116 species from all sections of the genus and nearly its entire distribution area were analyzed. Divergence times were estimated under a random molecular clock based on nrITS phylogeny, which was the most complete data set regarding the representation of species and distribution areas. Ancestral distribution ranges were estimated for the nrITS data set as well as for a combined data set, revealing that *Gagea* most likely originated in southwestern Asia. This genus first diversified there starting in the Early Miocene. In the Middle Miocene, *Gagea* migrated to the Mediterranean and to East Asia, while migration into Euro‐Siberia took place in the Late Miocene. During the Pleistocene, the Arctic was colonized and *Gagea serotina,* the most widespread species, reached North America. The Mediterranean basin was colonized multiple times from southwestern Asia or Euro‐Siberia. Most of the currently existing species originated during the last 3 Ma.

## 1 INTRODUCTION

The Irano‐Turanian (IT) floristic region has been considered crucial for plant species composition as it is also a source of taxa to colonize neighboring areas, especially the Mediterranean and the Saharo‐Arabian floristic region (Manafzadeh, Salvo, & Conti, 2014; Manafzadeh, Staedler, & Conti, 2017). Some studies could show that the radiation of several plant lineages most probably started in the IT floristic region (e.g., Brassicaceae tribe Arabideae: Karl & Koch, 2013; other plant examples reviewed by Manafzadeh et al., 2017). Nevertheless, this kind of studies is still limited (Manafzadeh et al., 2017).

Different opinions on the geographical border limits of the IT floristic region exist (e.g., Djamali, Brewer, Breckle, & Jackson, 2012; reviewed by Manafzadeh et al., 2017). According to a detailed climatic study (Djamali et al., 2012), the IT floristic region (southwestern Asia) is characterized by cold winters, hot summers, high continentality index, generally low mean annual precipitations concentrated in winter and spring. Such a climatically distinct region corresponds to the western portion of the IT region as defined by Manafzadeh et al. (2017).

Southwestern Asia harbors about 27,000 vascular plant species (Sales & Hedge, 2013), and it is characterized by semideserts and steppes at different altitudes (Manafzadeh et al., 2017). Within this area, the species‐rich mountains of the Thian Shan and the Pamir (the easternmost IT subregion, according to Djamali et al., 2012) are a biodiversity hot spot, inhabited by a high number of endemics (Manafzadeh et al., 2017). A few biogeographic studies could show the important role of this latter area for diversification processes and for colonization of neighboring areas. Malik et al. (2017) suggested that diversification processes in *Artemisia* subg. *Seriphidium* started in the Thian Shan, Pamir, and Hindu Kush mountain ranges and that this subgenus subsequently expanded to Eurasia.

**Figure 1:**
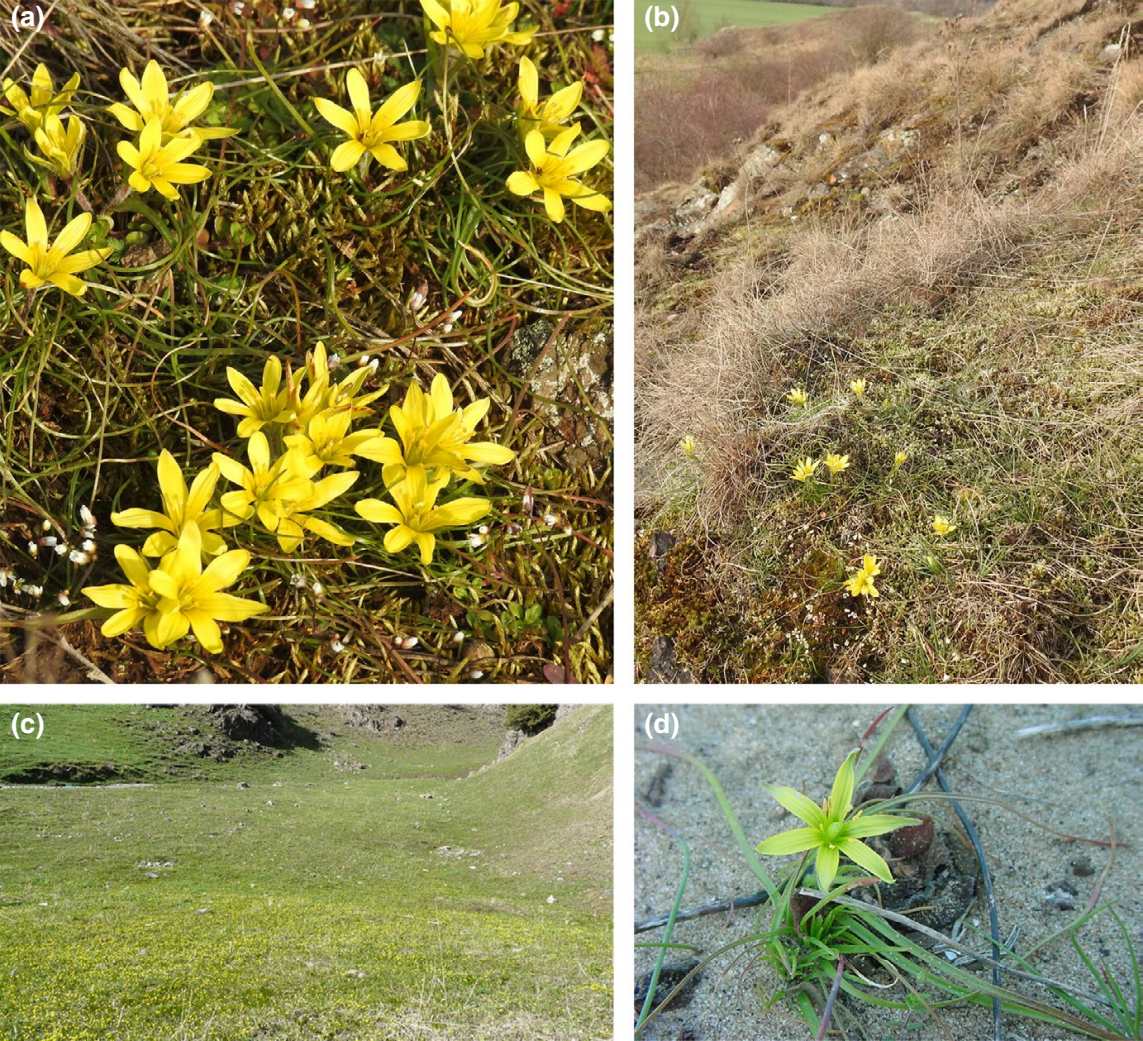
(a) *Gagea bohemica *(Germany, Saxony‐Anhalt, Mücheln nearby Wettin); (b) *Gagea bohemica*; rocky slope with semiarid‐grassland Germany, Saxony‐Anhalt; (c) subalpine grassland in Bogda Shan, China, Xinjiang with different *Gagea* species (*G*. *angelae*, *G*. *nigra, G*. *huochengensis*); (d) *Gagea apulica* on sand in Italy, Apulia.

There is a lack of studies dealing with typical IT genera that also occur in neighboring areas. The genus *Gagea* Salisb. (Figure 1) shows the highest number of species (ca. 300) within Liliaceae (Peruzzi, 2016), and it occurs in different habitats, like dry steppe and Mediterranean grasslands, rocky slopes, alpine meadows, open scrublands, and deciduous forests. Mountain areas with different ecological and altitudinal conditions are usually more species‐rich than plain areas (Levichev, 1999). The genus represents a good case study to investigate the spatial and temporal relationships among southwestern Asia and its neighboring areas, for several reasons: (a) A high species richness (76%; Levichev, 1999) occurs in southwestern Asia. (b) A relevant diversity (about 100 species: Levichev, 1999; Peterson et al., 2016) can be found in the IT hot spot areas of western Thian Shan and the Pamir Alai. (c) Most of species (about 135 species) are narrow endemic to southwestern Asia (Levichev, 1999). (d) The genus as a whole is widely distributed in adjacent regions like Asia, Mediterranean basin, and temperate Europe. (e) The most widespread species, *Gagea serotina* (L.) Ker Gawl., also occurs in Arctic tundra in northern Asia, in western part of North America, and in Alpine vegetation zones of the Rocky Mountains (Meusel, Jäger, & Weinert, 1965). Finally, (f) a further species‐rich area is the Mediterranean basin, mostly colonized by the large lineage of *G*. sect. *Didymobulbos* (K.Koch) Boiss. (about 40 species; Tison, Peterson, Harpke, & Peruzzi, 2013).

While it has been shown that speciation in *Gagea* is influenced by polyploidization and recent intrasectional hybridization (e.g., Peterson et al., 2009; Peterson, Levichev, Peterson, Harpke, & Schnittler, 2011; Peterson et al., 2016; Tison et al., 2013; Zarrei et al., 2012), the impact of colonization processes and divergence time on the diversification in the genus is still unclear. Only for two sections of the genus, further speculations about age and origin were published. Levichev, Tuniyev, and Timukhin (2010) suggested a late Miocene origin in Asia Minor for *G*. sect. *Spathaceae* Levichev, and Zarrei et al. (2012) speculated that the ancestral population of *G*. sect. *Platyspermum* Boiss. occurred in Iran in the same period. However, the studies mentioned above were either too general, under sampled, or focused only on one particular group within *Gagea*. As a consequence, it is still unclear when and where the genus and its major lineages originated. In a recent study focused on the evolution of Liliaceae, Kim and Kim (2018) inferred an Asian origin (23.32–43.45 Ma) for *Gagea*, based on only a few accessions.

Therefore, our aim was to test the following three biogeographic hypotheses: (*H1*) The IT region is not only a current center of species diversity, but also a center of origin for the genus *Gagea* and its major linages (sections); (*H2*) the Mediterranean basin represents a secondary speciation center caused by in situ speciation; (*H3)* climate change played an important role for colonization processes.

The reconstruction of evolutionary histories is possible by taking phylogenies into account in a biogeographic context and combining this information with divergence time estimations (e.g., Nieto Feliner, 2014). We based our ancestral range reconstruction and divergence time estimations on a well‐resolved ITS (ITS1 + 5.8S rRNA + ITS2) phylogeny, including about 40% of the existing species and representing 13 sections of the genus, sampled throughout their distribution range. Relationships inferred by the ITS region are in agreement with morphology and with the current classification of the genus (Peruzzi, 2012). The suitability of the ITS region and/or the usage of just one marker is often criticized (e.g., Álvarez & Wendel, 2003; Harpke & Peterson, 2006). In *Gagea*, potential conflicts could be caused by frequent hybridization. However, our extensive molecular and morphological studies had shown that hybridization takes place only on intrasectional level, between closely related species. This phenomenon is usually restricted to small areas of a specific phytogeographic region (Peterson et al., 2016, 2009, 2011; Peterson, John, Koch, & Peterson, 2004; Tison et al., 2013). As a consequence, we assume that ITS, biparentally inherited, is a suitable marker for our purpose, well reflecting phylogenetic relationships of major lineages (sections) of the genus. However, to address uncertainties connected with the usage of only one marker, ancestral area ranges were also estimated using a combined data set (ITS + two chloroplast intergenic regions). The chloroplast data set (*psb*A‐*trn*H IGS + *trn*L‐*trn*F IGS) was analyzed separately, to detect possible incongruences and to get further insights into the biogeographic distribution of chloroplast haplotypes, by calculating haplotype genealogy using a parsimony network approach. The latter approach is more suitable to infer relationships when the resolution of phylogenetic trees is rather low, which was the case for chloroplast markers in *Gagea* even when several markers were combined together (Peterson, Levichev, & Peterson, 2008; Zarrei et al., 2009) albeit they were generally in congruence to ITS phylogenies concerning the major lineages (sections).

## 2 MATERIAL AND METHODS

### 2.1 Taxonomic treatment and infrageneric classification of *Gagea* Salisb

Concerning the infrageneric classification of *Gagea*, different taxonomic systems have been proposed, based on different weighting of morphological and/or molecular phylogenetic features (see Table 1).

**Table 1:** Comparison of infrageneric classifications of *Gagea* Salisb. appeared in the last 10 years. Taxonomical units on genus level are indicated in bold and italics

Levichev (2013) (see also Peterson et al., 2008)	Zarrei et al. (2011)	Peruzzi (2012)
***Gagea*** Salisb.	***Gagea*** Salisb.	***Gagea*** Salisb.
*Gagea*	*Gagea*	*Gagea*
*Didymobulbos* (K.Koch) Boiss.	*Didymobulbos* (K.Koch) Boiss.	*Didymobulbos* (K.Koch) Boiss.
*Fistulosae* (Pascher) Davlian.		
*Minimae* (Pascher) Davlian.		*Minimae* (Pascher) Davlian.
*Spathaceae* Levichev		*Spathaceae* Levichev
*Stipitatae* (Pascher) Davlian.		*Stipitatae* (Pascher) Davlian.
*Dschungaricae* Levichev		
		*Persicae* (Levichev) Peruzzi
*Plecostigma* (Turcz.) Pascher	*Plecostigma* (Turcz.) Pascher	*Plecostigma* (Turcz.) Pascher
*Platyspermum* Boiss.	*Platyspermum* Boiss.	*Platyspermum* Boiss.
*Graminifoliae* Levichev		
*Incrustatae* Levichev		*Incrustatae* Levichev
*Bulbiferae* Levichev		*Bulbiferae* Levichev
*Anthericoides* A.Terracc.	*Anthericoides* A.Terracc.	*Anthericoides* A.Terracc.
***Lloydia*** Salisb. ex Rchb.	*Lloydia* (Salisb. ex Rchb.) Peruzzi, J.‐M.Tison, A.Peterson & J.Peterson	*Lloydia* (Salisb. ex Rchb.) Peruzzi, J.‐M.Tison, A.Peterson & J.Peterson
	*Tricholloydia* (Engl.) Zarrei & Wilkin	*Tricholloydia* (Engl.) Zarrei & Wilkin
***Kharkevichia*** Levichev		*Triflorae* Peruzzi

One of the main disagreements among authors concerns the inclusion of the former genus *Lloydia* Salisb. ex Rchb. in *Gagea*. While I.G. Levichev (Levichev, 2013; Peterson et al., 2008) keeps these genera as separate, other authors include it in *Gagea*.

Recently, the two sections of the former genus *Lloydia* were transferred under *Gagea*: *G*. sect. *Lloydia* (Rchb.) Peruzzi, Tison, Tison, Peterson, and Peterson (2008), and *G*. sect. *Tricholloydia* (Engl.) Zarrei, Wilkin, Ingrouille, and Chase (2011).

Another point of dispute concerns the putative genus *Kharkevichia* Levichev (2013), which is considered as a further section of *Gagea*, namely *G*. sect. *Triflora*e by Peruzzi (2012).

Two species of the former genus *Lloydia* (*Lloydia tibetica*: “*tib*”, *L. yunnanensis*: “*yun*”) are not yet assigned to any specific section, since they lack molecular and/or morphological data. The provisional subdivision of *Gagea* in 14 sections, as circumscribed by Peruzzi (2012), is adopted here (Table 1).

### 2.2 Sampling, definition of phytogeographic units, and map reconstruction

In total, 517 accessions were included in this study, representing 116 species. Of them, 107 accessions (28 species; originating from herbarium vouchers; for details see Appendix Table S1) were newly investigated here, including DNA isolation. Information on species distribution is based on data published by Ali and Levichev (2007), Levichev and Jezniakowsky (2008), Meusel et al. (1965), Tison et al. (2013), Xinqi and Turland (2000), and own data of Peterson et al., 2011; Peterson et al., 2016 (for details, see Appendix Table S1). All new samples used in this paper were provided as herbarium material and were collected before 2015.

The distribution of the genus *Gagea* was categorized in seven phytogeographic units (PUs hereafter), largely based on a previous study published by Levichev (1999). These PUs are as follows: (a) southwestern Asia (A‐SW) including North and East Anatolia, Iraq, Iran, Afghanistan, the Middle Asia states, and South of Kazakhstan (Levichev, 1999; Peterson et al., 2009; see also Djamali et al., 2012); (b) Mediterranean (M); (c) East Asia (A‐E); (d) Euro‐Siberia (ES); (e) Himalaya (A‐H); (f) Circumboreal (Bo); and (g*)* Arctic.

**Figure 2:**
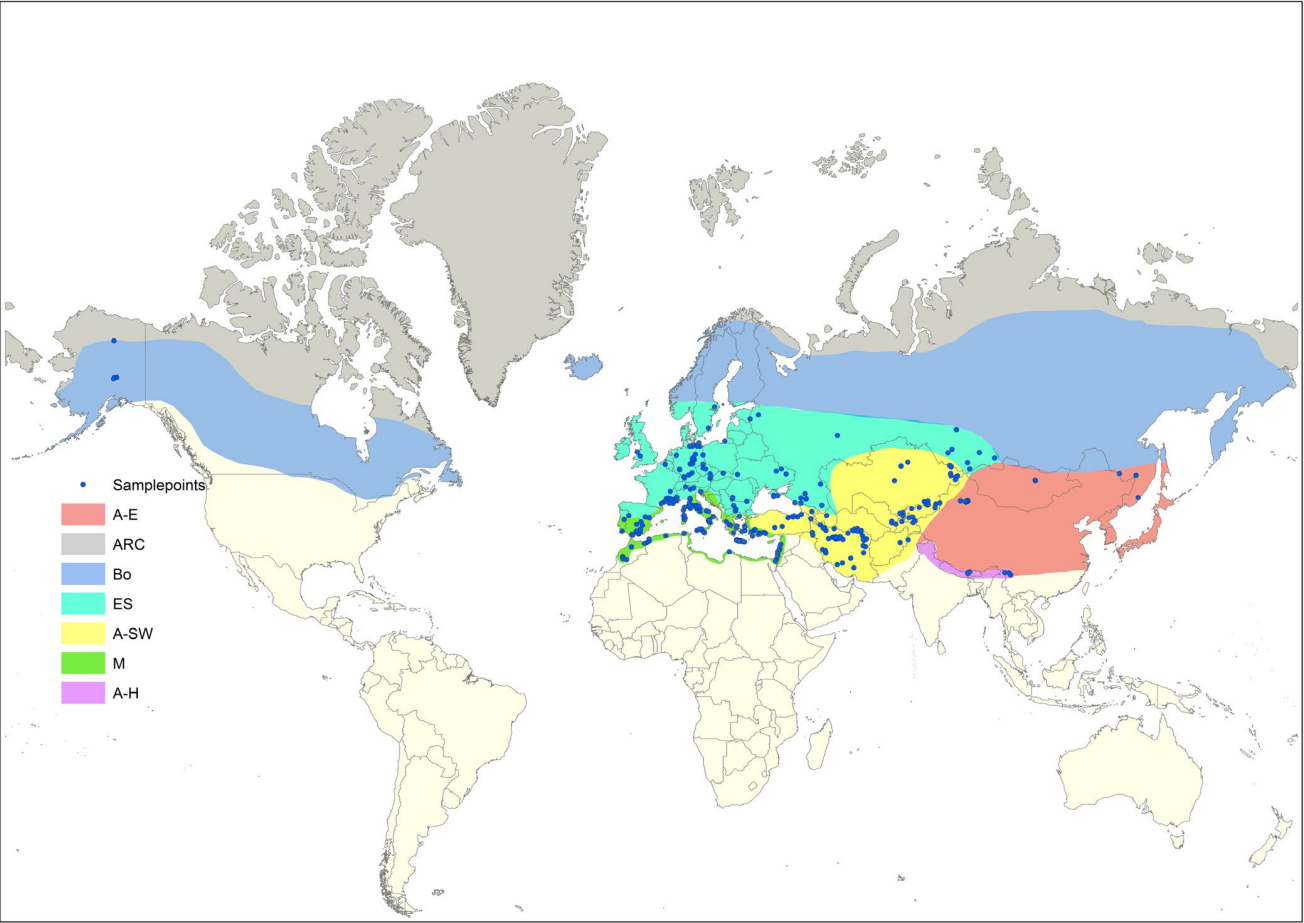
Map of the investigated representatives of *Gagea* (including 504 samples; indicated by dots) distributed in the six investigated PUs (for color code see legend). The map was generated using ESRI ArcGIS software (ESRI 2017; ArcGIS Desktop: Release 10.6). In some cases, one dot stands for different samples cooccurring in the same area. For further details see Appendix Table S1

A distribution map of the investigated representatives of *Gagea* within six phytogeographic units was generated. No material of *G. serotina* originating from the Arctic PU could be included (Appendix Table S1). The map (including 504 localities; Figure 2) was generated using ESRI ArcGIS software (ESRI 2017. ArcGIS Desktop: Release 10.6). For most of the accessions we relied on coordinates collected during our fieldwork or from the information available on herbarium labels (for details see Appendix Table S1). For older herbarium vouchers, where only information of the collection area was available, the localization was derived with an uncertainty of several kilometers.

### 2.3 DNA extraction, PCR amplification, and sequencing

DNA isolation, amplification of the ITS region (ITS1 + 5.8S rRNA + ITS2) and the two chloroplast markers *psb*A‐*trn*H IGS and *trn*L‐*trn*F IGS was carried out as described by Peterson et al. (2016). Gel‐purified PCR products (50–200 ng) were prepared and both strands were sequenced as “u‐mixes” via Sequencing Service (StarSeq, Mainz, Germany).

### 2.4 Phylogenetic analyses

All sequences newly obtained in this project (20 ITS, 101 *psb*A‐*trn*H IGS, 104 *trn*L‐*trn*F IGS) were deposited in the European Nucleotide Archive (ENA; Appendix Table S1). In addition, we included sequences of our previous molecular investigations (Peruzzi, Peterson, Tison, & Harpke, 2011; Peruzzi, Peterson, Tison, & Peterson, 2008; Peruzzi, Tison, et al., 2008; Peterson et al., 2016, 2009, 2010, 2004, 2008, 2011; Pfeiffer, Klahr, Peterson, Levichev, & Schnittler, 2012; Tison et al., 2013) and sequences from ENA deposited by other authors (Wörz, Hohmann, & Thiv, 2012; Zarrei et al., 2009). In total, the ITS data set included 418 sequences (107 species). Sequences of both chloroplast markers (*psb*A‐ *trn*H IGS, *trn*L‐*trn*F IGS) of 497 individuals (113 species) were concatenated and further analyzed together. The combined data set of (ITS + cpDNA) included 265 combined sequences corresponding to 103 species (for further details see Table 2; Appendix Table S1).

**Table 2:** Number of estimated and investigated species (Appendix Table S1) for each of the *Gagea* sections recognized by Peruzzi (2012)

				No. of investigated species		
No.	*Gagea* section^a^	Acronym	No. of estimated species	ITS	cpDNA	ITS + cpDNA
1	*Anthericoides*	ANT	2	2	2	2
2	*Bulbiferae*	BUL	~7	2	2	2
3	*Didymobulbos*	DID	>43	36	36	36
4	*Gagea*	GAG	>59	24	24	23
5	*Incrustatae*	INC	~8	1	1	1
6	*Lloydia*	LOY	2	1	2	1
7	*Minimae*	MIN	~9	6	6	6
8	*Persicae*	PER	1	1	1	1
9	*Platyspermum*	PLA	~56	14	15	12
10	*Plecostigma*	PLE	~35	9	10	9
11	*Spathaceae*	SPA	1	1	1	1
12	*Stipitatae*	STIP	~65	8	9	8
13	*Tricholloydia*	TRIL	~2	0	2	0
14	*Triflorae*	TRIF	1	1	1	1
	n.d.^b^	*	~4	1	1	0
	Whole genus		>295	107	113	103

Note

No, number. n.d., not determined.

a For infrageneric classification see Table 1.

b Species are not assigned to a specific section caused by the lack of further morphological or molecular data.

The final data sets include *Gagea* species representing all 14 sections (plus two “*Lloydia*” taxa not yet assigned to *Gagea*: *Lloydia tibetica*: “*tib*” and *L. yunnanensis*: “*yun*”). As outgroup, we used representatives of other genera from tribes Tulipeae Duby and Lilieae Lam. & DC. (Appendix Table S1).

*Gagea* is a genus showing regular intrasectional hybridization (e.g., Peruzzi, 2008; Peterson et al., 2009; see also Introduction), resulting in (a) different species sharing identical sequences and (b) species which are not monophyletic in gene trees. Additionally, many *Gagea* species have different ribotypes and chloroplast haplotypes. As a consequence, for the purpose of our study, we relied on ribotypes and haplotypes and their geographical origin, rather than on species. ITS ribotypes and chloroplast haplotypes (HTs) were labeled and numbered at sectional level (further information concerning species is provided in Appendix Table S1). Only ITS and chloroplast sequences originating from the same accession were concatenated, and identical sequences were removed from the combined data set.

The number of parsimony‐informative ITS and cpDNA sites was determined using DnaSP v5.10.01, excluding gap sites (Librado & Rozas, 2009).

ITS sequence data set (418 sequences) and combined data set (ITS type + cpDNA haplotypes; 265 sequence types) were used for preliminary phylogenetic analyses using Bayesian phylogenetic inference with MRBAYES 3.2. (Ronquist et al., 2012) to evaluate whether the inferred topologies are in agreement with the ITS BEAST analyses. The analysis of the combined data set with two data partitions was carried out to check, if the results are congruent with the ITS phylogeny, and to use selected trees for the reconstruction of the ancestral distribution ranges. For Bayesian analyses (BA), 2 times 4 chains were run for six (ITS) and four (combined) million generations under the GTR+Γ model of sequence evolution, sampling a tree every 1,000 generations. Converging log‐likelihoods, potential scale reduction factors for each parameter and inspection of tabulated model parameters in MRBAYES suggested that stationarity had been reached in all analyses. The first 25% of trees of each run were discarded as burn‐in. Two independent runs of BA analysis were performed for each data set to confirm that separate analyses converged on the same result. Each of these two runs resulted in the same topology and similar posterior probabilities (pp) for nodal support.

### 2.5 Divergence time estimations and ancestral area reconstruction

Divergence time estimations based on ITS data were performed under a relaxed molecular clock. Due to the lack of fossils for geophytes like *Gagea*, a calibration is more difficult. Within Liliaceae, *Gagea* belongs to the tribe Tulipeae (*Amana*‐*Gagea‐Erythronium‐Tulipa* clade), where it is sister to the other three genera. The root of the *Gagea* phylogeny was calibrated using the crown age of the tribe Tulipeae, 39.9 Ma, provided by Givnish et al. (2016). A similar approach was used in the calibration of the root nodes of a recent *Cardiocrinum* (Lilieae) phylogeny (Yang et al., 2016). Independent estimations of the crown and stem ages for the tribe Lilieae by Huang et al. (2018) were similar to Givnish et al. (2016). In the recently published study of Kim and Kim (2018), the age estimated for tribe Tulipeae was older (39.48–66.86 Ma) in comparison to Givnish et al. (2016), and included also an estimation for genus *Gagea* (23.32–43.45 Ma). We used our ITS data set (418 sequences; 107 species) as input. BEAST (v.2.4.3; Bouckaert et al., 2014) analyses were run under a random local clock model for 4 × 10^8^ generations, logging parameters every 2,000 generations, and assuming a death–birth process in two independent runs. To address to slightly different published ages two calibrations (CAL1 and CAL2) were carried out. The crown age of tribe Tulipeae was set to 40 Ma with a sigma factor of 2 for a calibration (CAL1), according to Givnish et al. (2016). For CAL2, the crown age of *Gagea* was set to 35.5 Ma with a sigma factor of 6.5 to cover the range provided by Kim and Kim (2018). Convergence among chains and effective sample size of all parameters was calculated and visualized in Tracer v1.7 (Rambaut & Drummond, 2009). The trees of the two independent runs were joined using LogCombiner v.2.4.3 (Bouckaert et al., 2014) for each calibration. Twenty‐five percent of trees were removed as burn‐in, and summary statistics were calculated from the remaining trees using TreeAnnotator (BEAST v.2.4.3 package), to provide a summary tree. Since our combined data set (ITS and cpDNA) only included ca. 77% of the accessions and just two of the outgroups, it was not used for divergence time estimations.

We estimated ancestral ranges on the *Gagea* tree obtained from the BEAST analyses (ITS) and five trees out of the last 20 trees with different topologies of both runs inferred by Bayesian analysis (BA) of the combined data set (ITS + cpDNA) in BioGeoBEARS 1.1.2 (Matzke, 2013). BioGeoBEARS (Matzke, 2013,2014) allows testing various biogeographic models: DEC (dispersal‐extinction‐cladogenesis), DEC+J (J: extra parameter adding founder‐event speciation), DIVA (dispersal‐variance analysis), DIVA+J, BAYAREALIKE (a likelihood interpretation of BayArea), and BAYAREALIKE+J. The best model was chosen using AIC corrected for sample size (AICc).

### 2.6 cpDNA haplotype network

A statistical parsimony network (combined cpDNA data: *psb*A–*trn*H IGS and *trn*L–*trn*F IGS; 497 sequences; 113 species) was calculated using the algorithm of the R package “haplotypes” (Aktas, 2015) to obtain a chloroplast genealogy for the analyzed individuals and to get further insights into the biogeographic distribution of chloroplast haplotypes within the genus and its sections. Gaps were treated as missing data. Because of the uncertain homology of the sequence positions, (a) length variation at one mononucleotide repeat (T) in the *trn*L–*trn*F IGS was generally excluded for haplotype determination (resulting number of haplotypes: 169; number of these haplotypes per taxon and section are provided in Appendix Table S1), and (b) a highly variable region in the *psb*A‐*trn*H IGS was excluded for haplotype network construction of the whole genus, reducing the alignment length to 568 bp and reducing the total number of haplotypes (HT) to 98.

## 3 RESULTS

### 3.1 Phylogenetic analyses

The number of parsimony‐informative ITS sites within *Gagea* (alignment length: 783 bp with outgroups) was 234 (Table 3) and much higher compared to the 45 parsimony‐informative sites of the cpDNA marker (*psb*A‐*trn*H IGS + *trn*L‐*trn*F IGS; alignment length: 733 bp with outgroups).

**Table 3:** Number (No) of investigated *Gagea* species and sequences for ITS analyses, number of variable and parsimony‐informative ITS sites, for G. section acronyms see Table 2

Sections	No. investigated species	No. sequences	No. variable sites/total sites (excluding gaps)	Parsimony‐informative sites (Pi)
ANT	2	7	12/622	0 (0.0097)
BUL	2	14	6/616	6 (0.0049)
DID	36	171	184/586	140 (0.0446)
GAG	24	82	121/605	78 (0.0300)
INC	1	1	–	–
LOY	1	6	6/589	0 (0.0041)
MIN	6	43	41/613	29 (0.0186)
PER	1	4	8/816	0 (0)
PLA	14	43	66/621	32 (0.0207)
PLE	9	19	97/607	71 (0.0611)
SPA	1	8	0/615	0 (0)
STI	8	18	86/619	46 (0.0454)
TRIF	1	1	–	–
“*tib*”^a^	1	1	–	–
Total	107	418	266/529	234 (0.0881)

Note

a Species not assigned to a specific section caused by the lack of further morphological and molecular data.

The ITS data set included 418 sequences from 107 species (Table 2; Appendix Table S1), of which 234 were unique and included in the analysis.

**Figure 3:**
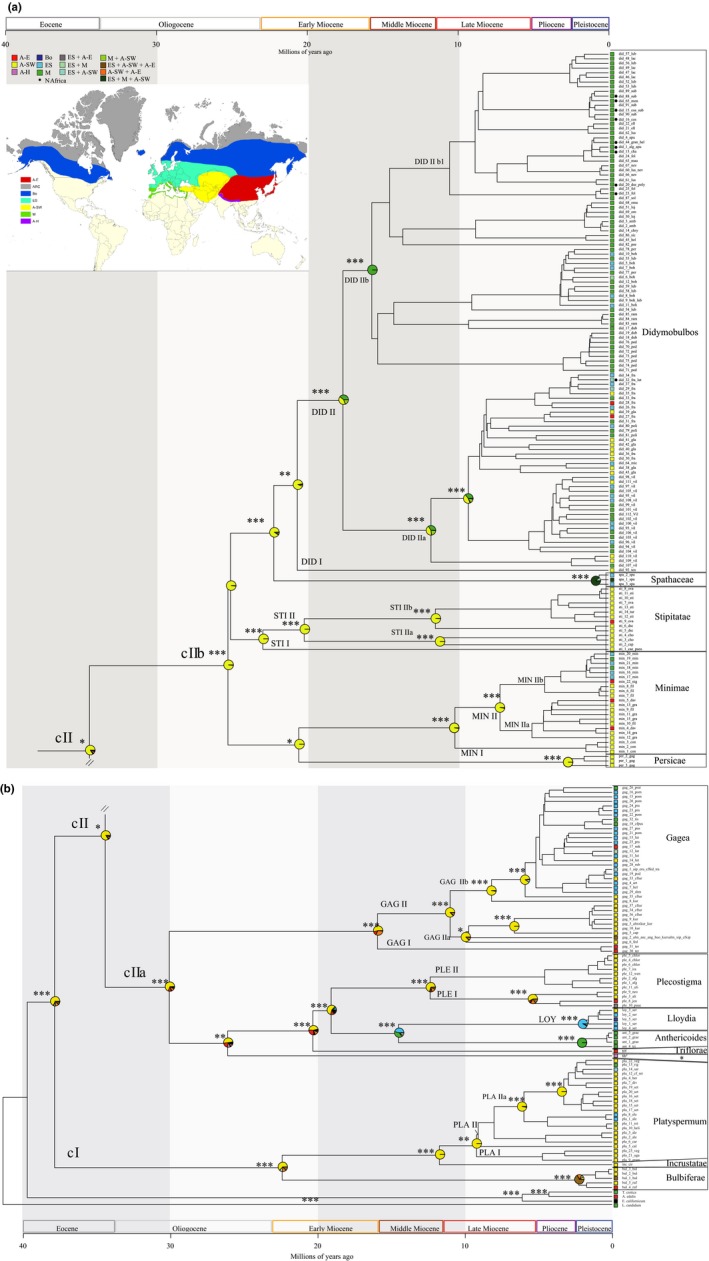
Ancestral area estimations for 418 ITS sequences of *Gagea*, using the DEC+J model in BioGeoBEARS. Probabilities for the inferred areas occupied by the ancestors are represented as pie charts at the nodes (for color code, see legend). ColoStates at nodes represent the “before the instantaneous speciation” event. State node of moderate‐to‐low support (pp < 0.90) has been removed. Posterior supports (with pp > 0.90) are indicated with asterisks (0.90–0.95*, 0.96–0.98**, 0.99–1.00***). The two major clades are marked as cI and cII. A‐E: East Asia; A‐H: Himalaya; A‐SW: southwestern Asia; Bo: Circumboreal; ES: Euro‐Siberia; M: Mediterranean. The outgroup *E. californicum* is distributed in the Mandrean Region, indicated by a black square. For the infrageneric classification of *Gagea* shown on the right side, see Appendix Table S1.

All *Gagea* sections were recovered as monophyletic with high support, (0.96) –0.99–1.00 pp, in the ITS tree (BEAST analyses; Figure 3 and Appendix Figures S1 and S2). The obtained phylogenetic tree is divided into two major clades: cI including *G*. sections *Bulbiferae* Levichev*, Incrustatae* Levichev and *Platyspermum*, and cII. The latter clade is divided into two major subclades: including *G*. sections *Anthericoides* A. Terracc., *Gagea*, *Lloydia* (Salisb. ex Rchb.) Peruzzi, J.‐M. Tison, A. Peterson & J. Peterson, *Plecostigma* (Turcz.) Pascher, *Triflorae* Peruzzi, and the sample *“tib”*; cIIb, including *G*. sections *Didymobulbos*, *Minimae* (Pascher) Davlian., *Persicae* (Levichev) Peruzzi, *Spathaceae*, and *Stipitatae* (Pascher) Davlian.

**Figure 4:**
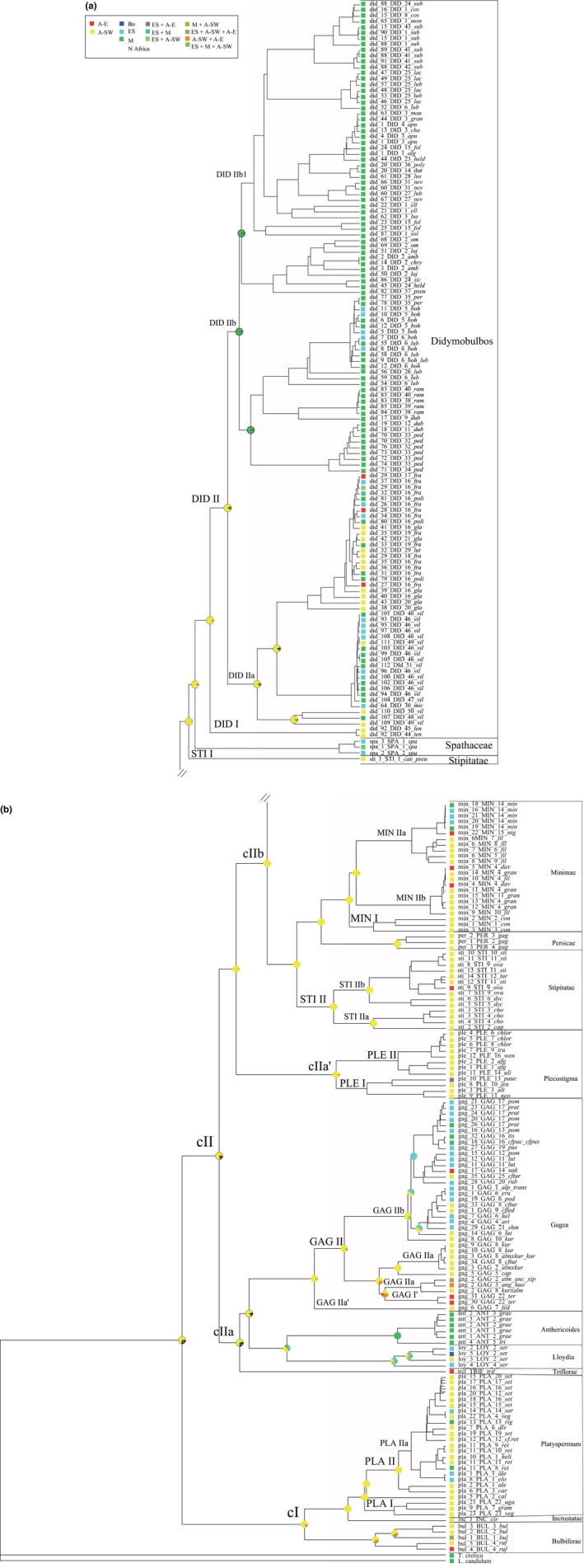
Ancestral range estimations for one selected tree inferred by MrBayes based on 265 sequences of the combined data set of the internal transcribed spacer (ITS) and cpDNA (*psb*A‐*trn*H IGS + *trn*L‐*trn*F IGS); ITS‐type_taxon_cpDNA‐haplotype (see Appendix Table S1); using the DEC+J model in BioGeoBEARS (see also Figure Appendix S4). Lineages which are not congruent with the ITS tree are marked with an apostrophe. A‐E: East Asia; A‐SW: southwestern Asia; Bo: Circumboreal; ES: Euro‐Siberia; M: Mediterranean

After concatenating ITS and chloroplast markers for each accession, a data set including 265 combined sequences was built. The phylogeny inferred by BA (Appendix Figure S3; see also Figure 4) was found to be congruent to the ITS BEAST tree (Figure 3) except for the position of *G*. sect. *Plecostigma* and two accessions, sharing the same sequences, of *G*. sect. *Stipitatae* (clade STI, Appendix Figure S3). Instead of being sister to *G*. sect. *Gagea*, *G*. sect. *Plecostigma* was found as sister to clade cIIb (pp 0.83; Appendix Figure S3). The backbone of clade cII (Appendix Figure S3) is unresolved and/or characterized by lower support values compared to those of the ITS tree (Figure 3). One combined sequence type of *G*. sect. *Stipitatae* (shared by *G. caelestis* Levichev and *G. pseudominutiflora* Levichev) is recovered as sister to *G*. sect. *Didymobulbos*, while all other sections are monophyletic with high support values (0.98–1.00 pp).

### 3.2 Divergence time estimation

Divergence time estimations using two different calibrations in BEAST (CAL1: using the divergence time of tribe Tulipeae according to Givnish et al., 2016; Figure 3; see also Appendix Figure S1; CAL2: using the crown age of the genus *Gagea* according to Kim & Kim, 2018; see Appendix Figure S2) were run for the ITS data. The estimated mean age of CAL2 was slightly lower and age ranges in general higher than in CAL1 (Table 4). However, the geological time scales were in the same range for both calibrations (Table 4). The topology of trees is identical for both calibrations. Therefore, the following description refers to both ITS trees and the total ranges for time estimation are provided.

**Table 4:** Results of Bayesian dating analysis (BEAST) based on the ITS data, mean node ages of major clades and crown ages and time of diversification of *Gagea* sections

Clade	Age (95% HPD) in Ma			
Calibration^a^	CAL1	CAL2	CAL1 and CAL2	Time of diversification of *Gagea* sections
Genus	37.84 (42.66–32.62)	(43.45–23.32)^b^	43.45–23.32^b^	
cI	22.39 (27.79–17.05)	17.69 (25.32–10.50)	27.79–10.50	
INC	n.d.			n.d.
PLA	9.19 (11.73–6.63)	6.58 (13.11–5.12)	13.12–5.12	Late Miocene
BUL	2.25 (3.93–0.88)	1.16 (2.39–0.34)	3.93–0.34	Pleistocene
cII	34.40 (39.62–28.56)	28.95 (40.04–18.83)	40.04–18.83	
cIIa	30.05 (35.77–24.10)	22.78 (35.65–16.32)	35.77–16.32	
“*tib*”^c^	n.d.			n.d.
TRIF	n.d.			n.d.
GAG	15.92 (20.07–11.91)	12.79 (18.65–7.63)	20.07–7.63	Middle/Late Miocene
PLE	12.34 (16.49–8.36)	10.16 (15.63–5.56)	16.49–5.56	Middle/Late Miocene
ANT	2.03 (3.23–0.94)	1.05 (2.01–0.40)	3.23–0.40	Pleistocene
LOY	1.98 (3.86–0.42)	1.12 (2.32–0.32)	3.86–0.32	Pleistocene
cIIb	25.22 (30.06–20.44)	20.34 (28.11–12.82)	30.06–12.82	
STI	22.90 (27.75–18.06)	18.35 (25.82–11.50)	27.75–11.50	Early/Middle Miocene
DID	20.62 (24.93–16.49)	17.49 (22.27–10.12)	24.93–10.12	Early/Middle Miocene
MIN	10.17 (13.74–6.72)	7.19 (11.30–4.01)	13.74–4.01	Late Miocene/(Pliocene)
PER	2.86 (4.52–1.02)	1.57 (3.10–0.52)	4.52–0.52	(Pliocene)/Pleistocene
SPA	0.77 (1.85–0.01)	0.28 (0.95–0)	1.85–0	Pleistocene

Note

n.d., not determined.

a CAL1: divergence time of the tribe *Tulipeae* according to Givnish et al. (2016) (Figure 3 and Appendix Figure S1), CAL2: crown age of the genus *Gagea* according to Kim and Kim (2018) (Appendix Figure S2).

b Calibration point CAL2 (see Material and Methods).

c Not assigned to a specific section (Table 2).

The diversification of sections within all major clades started at different times (Table 4; Figure 3; Appendix Figure S1). In clade cI (27.8–10.5 Ma) diversification of the current lineages of *G*. sect. *Platyspermum* started in the Late Miocene, while for *G*. sect. *Bulbiferae* it started in the Pleistocene. Within clade cII (40.0–18.8 Ma), for *G*. sections *Gagea* and *Plecostigma* crown ages in the Middle/Late Miocene were revealed, while diversification of *G*. sections *Anthericoides* and *Lloydia* started more recently, during the Pleistocene. Also within clade cIIb (30.1–12.8 Ma), different crown ages were found for the sections: Early/Middle Miocene for *G*. sections *Stipitatae* and *Didymobulbos*, Late Miocene/(Pliocene) for *G*. sect. *Minimae*, (Pliocene)/Pleistocene for *G*. sect. *Persicae*, and Pleistocene for *G*. sect. *Spathaceae*.

Nearly 2/3 (ca. 64%) of the investigated *Gagea* species originated during the Pleistocene.

### 3.3 Reconstruction of ancestral distribution ranges

The results of the ancestral range reconstruction based on the ITS data using six different models (for model comparison see Table 5) are mostly in agreement (results are summarized in Table 6, see also Figure 3). According to AIC criteria, the DEC+J was the best fitting model for our data set: the genus (68%), the major clades (cI: 63%; cII 84%), and both subclades of cII (cIIa: 69%; cIIb: 98%) most likely originated in southwestern Asia. According to the other models (see Table 6), an even higher probability for a southwestern‐Asian origin was found for the genus (98%–100%) and for all major clades and subclades (cI: 99%–100%; cII: 97%–100%; cIIa: 81%–92%; cIIb: 100%). In principle, the results of ancestral area reconstruction under the different models were identical for all the sections, with the exception of *G*. sections *Bulbiferae, Gagea* and *Spathaceae* (Table 6).

**Table 5:** Results from model comparison based on ITS data between six biogeographic models

			Parameter estimates				
Model	Likelihood	numparams	*d*	*e*	*j*	AIC	AIC analysis weight ration
DEC	−372.7	2	0.0079	0.0042	0	749.3	1.00E−27
DEC+J	−309.5	3	0.0024	1.00E−12	0.025	625.1	1
DIVALIKE	−371.5	2	0.0099	0.0026	0	747.1	3.10E−27
DIVALIKE+J	−314.9	3	0.0030	1.00E−12	0.024	635.8	0.0046
BAYAREALIKE	−324.7	3	0.0020	1.00E−12	0.028	655.5	2.50E−07
BAYAREALIKE+J	−324.7	3	0.0020	1.00E−12	0.028	655.5	2.50E−07

**Table 6:** Results of ancestral area determination based on ITS data by different models and current distribution of clades

		Probabilities of six biogeographic models						Current distribution in phytogeographic units						
Clade	Most probable ancestral areas	DEC+J	DEC	DIVA	DIVA+J	BAYAREALIKE	BAYAREALIKE+J	A‐SW	A‐H	A‐E	M	ES	Bo	Arc
Genus *Gagea*	A‐SW	0.68	1.00	0.99	0.98	1.00	1.00	×	×	×	×	×	×	×
cI	A‐SW	0.63	1.00	0.99	0.99	1.00	1.00	×	×	×	×	×		
INC	n.d.							×	×					
PLA	A‐SW	1.00	1.00	1.00	1.00	1.00	1.00	×	×		×	×		
BUL	A‐SW	0.09	1.00	0.90	0.95	1.00	1.00	×		×		×		
	A‐SW + A‐E + ES	0.67	0	0	0.02	0	0							
cII	A‐SW	0.84	0.99	0.97	0.97	1.00	1.00							
cIIa	A‐SW	0.69	0.92	0.81	0.85	0.92	0.92	×	×	×	×	×	×	×
“*tib*”^a^	n.d.								×					
TRIF	n.d.									×				
GAG	A‐SW	0.54	0.90	0.04	0.44	0.90	0.90	×	×	×	×	×		
	A‐SW + AE	0.31	0	0.93	0.51	0	0							
PLE	A‐SW	0.69	0.93	0.95	0.93	0.93	0.93	×	×	×	×	×		
ANT	M	1.00	1.00	1.00	1.00	1.00	1.00				×			
LOY	ES	0.91	0.99	0.99	0.98	0.99	0.99	×	×	×		×	×	×
cIIb	A‐SW	0.98	1.00	1.00	1.00	1.00	1.00	×	×	×	×	×	×	
STI	A‐SW	1.00	1.00	1.00	1.00	1.00	1.00	×	×	×	×			
DID	A‐SW	0.92	0.98	0.60	0.91	0.98	0.97	×	×	×	×	×	×	
MIN	A‐SW	0.99	1.00	1.00	1.00	1.00	1.00	×	×	×	×	×	x	
PER	A‐SW	1.00	1.00	1.00	1.00	1.00	1.00	×			×			
SPA	A‐SW + M	0	0.49	0	0.12	0.49	0.49	×			×	×		
	ES	0	0.49	0.96	0.62	0.49	0.49							
	A‐SW + ES+M	0.88	0	0.03	0.23	0	0							

a Not assigened to a specific section (see Table 2; Appendix Table S1).

Ancestral ranges were also estimated for the combined data set (ITS + cpDNA; Figure 4 and Appendix Figure S4) using five trees of the BA showing a different topology for the unresolved or low supported clades. According to AIC criteria, the DEC+J was also the best fitting model for this data set (Table 7).

**Table 7:** Results from model comparison based on the combined data set (ITS + cpDNA) between six biogeographic models

			Parameter estimates				
Model	Likelihood	numparams	*d*	*e*	*j*	AIC	AIC analysis weight ration
DEC	−351.3	2	0.0092	1,00E−12	0	706.7	1.30E−29
DEC+J	−283.8	3	0.0027	1.00E−12	0.028	573.6	0.9900
DIVALIKE	−381.6	2	0.0130	0.0028	0	767.3	8.90E−43
DIVALIKE+J	−291.5	3	0.0031	1.00E−12	0.028	589.0	0.0005
BAYAREALIKE	−289.3	3	0.0020	1.00E−12	0.030	584.5	0.0043
BAYAREALIKE+J	−289.3	3	0.0020	1.00E−12	0.030	584.5	0.0043

The most probable ancestral region for the major lineages (shown for one tree in Table 8) are congruent with the ITS analysis (Table 6). However, this data set missed the accessions from the Himalaya, for which only ITS was available. Thus, only five PUs were considered in this case.

**Table 8:** Results of ancestral area determination based on combined data (ITS + cpDNA) data by different models

		Probabilities of six biogeographic models					
Clade	Most probable ancestral areas	DEC+J	DEC	DIVA	DIVA+J	BAYAREALIKE	BAYAREALIKE+J
Genus *Gagea*	A‐SW	0.73	0.15	0.94	0.97	0.99	0.99
	A‐SW + A‐E + M	0	0.53	0.02	0	0	0
cI	A‐SW	0.81	0.83	0.99	1.00	1.00	1.00
INC	n.d.						
PLA	A‐SW	1.00	1.00	1.00	1.00	1.00	1.00
BUL	A‐SW	0.65	0.85	0.93	0.98	1.00	1.00
cII	A‐SW	0.80	0.12	0.93	0.95	0.99	0.99
	A‐SW + M	0.03	0.08	0.01	0	0	0
	A‐SW + A‐E + M	0.01	0.63	0.02	0	0	0
cIIa	A‐SW	0.71	0.12	0.33	0.76	0.93	0.93
	A‐SW + M	0.02	0	0.56	0.01	0	0
	A‐SW + A‐E + M	0.05	0.71	0.04	0	0	0
TRIF	n.d.						
GAG	A‐SW	0.93	0.89	1.00	1.00	1.00	1.00
ANT	M	1.00	1.00	1.00	1.00	1.00	1.00
LOY	ES	0.35	0	0.06	0.34	0.53	0.53
	A‐SW	0.24	0	0	0.23	0.46	0.46
	A‐SW + Bo+ES	0.05	0.73	0.76	0.09	0	0
cIIa’	A‐SW	0.99	0.99	1.00	1.00	1.00	1.00
PLE	A‐SW	0.98	0.87	1.00	1.00	1.00	1.00
cIIb	A‐SW	0.99	0.99	1.00	1.00	1.00	1.00
ST I	n.d.						
STI II	A‐SW	1.00	1.00	1.00	1.00	1.00	1.00
DID	A‐SW	0.97	0.81	0.96	0.99	1.00	1.00
MIN	A‐SW	1.00	0.96	1.00	1.00	1.00	1.00
PER	A‐SW	1.00	1.00	1.00	1.00	1.00	1.00
SPA	ES	0.01	0	0.30	0.55	0.50	0.50
	A‐SW + M	0	0	0	0.14	0.44	0.44
	A‐SW + ES+M	0.75	0.81	0.60	0	0	0

The diversification of most sections originating in southwestern Asia (ITS tree; Figure 3) started in the Miocene (Early/Middle Miocene: *G*. sections *Didymobulbos* and *Stipitatae*; Middle/Late Miocene: *G*. sect. *Plecostigma*; Late Miocene: *G*. sections *Minimae* and *Platyspermum*), but *G*. sect. *Persicae* diversified in the same area later during the (Pliocene)/Pleistocene. Also considering the accessions within each section, early diverging lineages are from A‐SW. On the contrary, accessions from ES, M, and A‐E diversified recently (see also the combined trees: ITS + cpDNA; Appendix Figure S3 and Figure 4) starting at the end of the Late Miocene (Figure 3). Examples (Figures 3 and 4) are the representatives in ES and M (PLA IIa) of *G*. sect. *Platyspermum* (cI) and in ES, M, and A‐E (PLE I) of *G*. sect. *Plecostigma* (cIIa). In *G*. sect. *Minimae* (clade cIIb) samples from A‐E were found in the more recently branching clade MIN II (end of the Late Miocene/Pliocene). In addition to A‐SW and A‐E representatives, in one group (MIN IIb; Pliocene) also ribotypes from M and ES were found. In *G*. sect. *Stipitatae*, ribotypes from A‐E can be found in the relatively young subclade STI IIb (Pliocene), whereas STI I and STI IIa include only representatives of A‐SW. During the Early Miocene/Middle Miocene (Figure 3), a deep split separated *G. tenera* Pascher (clade DID I) from all other investigated samples of *G*. sect. *Didymobulbo*s (see also Figure 4). In subclade DID IIa (Late Miocene, Figure 3; see also Figure 4), ribotypes from A‐SW and ribotypes from other PUs (ES, M, A‐E) were found together, whereas subclade DID IIb mostly consists of ribotypes from M, with a few ribotypes from ES. In clade DID IIa, the ribotypes of *G. fragifera* (Vill.) Ehr.Bayer & G.López (“*fra*”) diversified in A‐E, M, and ES since the Late Miocene (Figure 3).

Considering the position of N African accessions within *G*. sect. *Didymobulbos* in our phylogenetic trees (Figures 3 and 4), it can be recognized, that with the exception of *G. fragifera* from Morocco (subclade DID IIa) all other accessions from North Africa (Algeria, Morocco, and Tunisia) are in subclade DID IIb1, consisting of Mediterranean species only. Several ITS ribotypes occurring in North Africa were also found in samples from other Mediterranean regions (e.g., Morocco/Spain: “*did15,*” “*did88*”; Morocco/Algeria/Spain: “*did20*”; Algeria/Sardinia: “*did23*”; Tunisia/Sardinia/Sicily: “*did44*”; Figures 3 and 4; Appendix Table S1).

All the sections for which A‐SW was not recognized as the most probable ancestral area (according to the DEC+J model; ITS tree: Table 6 and combined tree (ITS + cpDNA) Table 8) diversified more recently (Table 6), during the Pleistocene (the Mediterranean *G*. sect. *Anthericoides*; *G*. sect. *Lloydia*; *G*. sect. *Spathaceae*). Within *G*. sect. *Anthericoides*, *Gagea trinervia* (Viv.) Greuter (“*tri*”) split very early from *G. graeca* (L.) Irmsch (“*grae*”). Within *G*. sect. *Lloydia* (LOY), accessions from Bo (“*loy5*”; N America) and A‐SW (“*loy*3”) split from samples from ES about 0.8 Ma (Figure 3) or more recently (Appendix Figure S2).

### 3.4 cpDNA haplotype network

The analysis of cpDNA sequences of 497 individuals representing 113 species (Appendix Table S1) identified 98 haplotypes (Table 9), of which most can be found in only one PU. Only 23 haplotypes are shared across different PUs.

**Table 9:** *Gagea* cpDNA haplotypes (HTs) used for network reconstruction after exclusion of highly variable regions, number of haplotypes sharing different phytogeographic regions (PUs) and corresponding clade of the cpDNA haplotype network

Section	Clade^b^	No. of sequences^a^	HTs^b^	Missing HTs^b^	Total HTs^b^	Hts sharing PUs^b^
LOY	c1	14	2	0	2	1
PLE		24	8	14	22	2
TRIL		3	3	1	4	0
“*yun*”^c^		2	1	0	1	‐
ANT	c2	10	3	1	4	0
GAG		100	19	8	27	6
TRIF		1	1	0	1	‐
DID	c3	199	25	13	38	5
MIN		49	7	0	7	3
PER		4	3	2	5	0
SPA		8	1	0	1	1
STI		25	6	8	14	1
BUL	c4	14	3	2	5	2
INC		1	1	0	1	‐
PLA		43	15	5	20	2
Total		497	98	54	152	23

Note

No, number.

a For further details see Appendix Table S1.

b See Figure 5.

c Not assigned to a specific section (see Table 1; Appendix Table S1).

**Figure 5:**
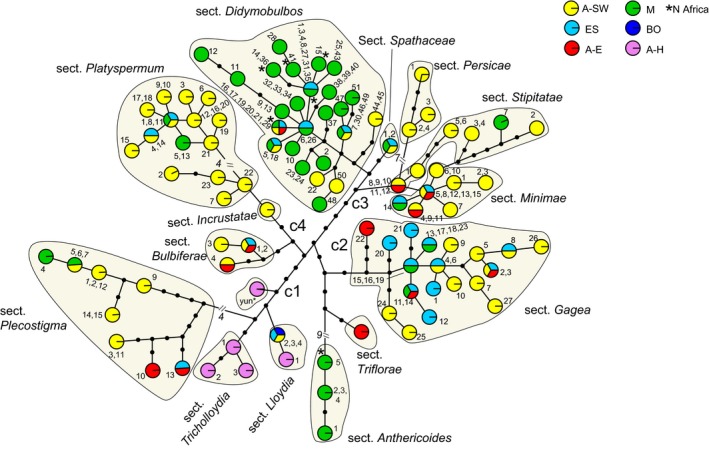
Chloroplast DNA haplotype genealogy of 497 sequences (*psb*A‐*trn*H IGS + *trn*L‐*trn*F IGS) of *Gagea* inferred using the R package haplotypes. A total of 152 haplotypes were recognized, 54 of them occurring as missing intermediates (black dots). Major lineages are marked as c1, c2, c3, and c4. Phytogeographic units of the haplotypes are marked by different colors. A‐E: East Asia; A‐H: Himalaya; A‐SW: southwestern Asia; Bo: Circumboreal; ES: Euro‐Siberia; M: Mediterranean

Four major clusters (c1 to c4; Figure 5) were recognized. They are congruent with the major clades recovered by the ITS tree (Figure 3), with the exception of clade IIa (ITS tree), that is split in two separate major clusters (c1 and c2) in the chloroplast haplotype network.

Haplotypes from the A‐H and Bo PUs were only found in cluster c1, which includes *G*. sections *Lloydia* (one haplotype A‐H and one haplotype: A‐SW/Bo/ES), *Plecostigma* (A‐SW, A‐E, ES, and M), *Trichollyodia* (A‐H), and “*yun*” (A‐H).

*Gagea* sect. *Incrustatae* (A‐SW; c4, Figure 5) is only one mutation step away from *G*. sect. *Platyspermum* (c4). About 93.7% of all detected haplotypes of the latter section can be found in A‐SW. Within *G*. sect. *Platyspermum*, a few A‐SW samples either have haplotypes also found in ES and/or M and one haplotype, which was found only in the Mediterranean, was one mutation step away from A‐SW haplotypes.

Some sections are split into different lineages (Figure 5), containing in addition to A‐SW haplotypes, haplotypes from different neighboring PUs. Examples are *G*. sect. *Plecostigma* (c1), split in two clearly separated groups. The first group includes haplotypes found in A‐SW, A‐E, and ES while the second group includes haplotypes found in A‐SW and M. The haplotypes of samples outside A‐SW (M, ES, and A‐E) are found at the tips of the network. Also, *G*. sect. *Stipitatae* (c3; Figure 5) split in two groups. The first group includes haplotypes from A‐SW and A‐E, the second group includes haplotypes from A‐SW and M.

In the mostly Mediterranean *G*. sect. *Didymobulbos* (c3; Figure 5), haplotypes from A‐SW are either found close to the parent node of the section or shared across other PUs (M, ES, and A‐E). In contrast, samples from North Africa (Algeria, Morocco, and Tunisia) are found at the tips of the network. One of them is found in samples from four PU's (A‐SW, A‐E, M, and ES).

While the most distinct haplotype in *G*. sect. *Gagea* (c2; haplotype *“gag 22”;* Figure 5) was found in the accessions of *G. terraccianoana* from A‐E (Mongolia and East Russia), six out of 19 identified haplotypes are shared across samples from two or three different PUs.

In the Mediterranean *G*. sect. *Anthericoides* (c2; Figure 5), the most ancestral haplotype (*“ant 5”*) is found in the accessions of *G. trinervia* from Libya and Sicily, separated one‐to‐three steps from samples of *G. graeca* from Greece. *Gagea* sect. *Spathaceae* (c3; Figure 5) shows a single haplotype shared across A‐SW, E‐S, and M.

## 4 DISCUSSION

### 4.1 Southwestern Asia as the ancestral area for the genus and for most of its sections

According to divergence time estimations on ITS data, we inferred that the genus *Gagea* had a pre‐Miocene origin in the IT floristic region (southwestern Asia; corresponding to the western IT floristic region according to Manafzadeh et al., 2017). A much larger region (Asia) was postulated by Kim and Kim (2018) to be the most probable ancestral area for the genus.

The major lineages of *Gagea*, in most cases corresponding to sections, started to diversify from Early Miocene onwards (Early Miocene/Middle Miocene: *G*. sections *Didymobulbos* and *Stipitatae*; Middle/Late Miocene: *G*. sections *Gagea* and *Plecostigma*; Late Miocene: *G*. sections *Minimae* and *Platyspermum*). Also *G*. sect. *Persicae* diversified in southwestern Asia, but during the (Pliocene)/Pleistocene. While no estimate was available for other sections, our data are congruent with the hypothesis made by Zarrei et al. (2012) that *G*. sect. *Platyspermum* could have originated during late Miocene in the Irano‐Turanian Region.

Tectonic movements like the uplift and crustal shortenings in mountain regions (e.g., Thian Shan, Alborz, and Zagros Mountains), the formation of the Iranian and East Anatolian Plateau, the subsidence of the Caspian Basin were connected with climatic changes (e.g., cooling of uplift regions, development of rain shadows, aridification), and with an increase of habitat diversity. This created topographically isolated habitats in southwestern Asia since the late Miocene (for references on geological and climatic history of the IT floristic region, see the review of Manafzadeh et al., 2017). All these likely resulted in allopatric speciation with high species diversity and endemism degree. The climate, as an important abiotic factor (Djamali et al., 2012), mainly influenced distribution, radiation, and diversification processes of *Gagea* within southwestern Asia.

An origin and early diversification of *Gagea* in southwestern Asia is also in agreement with the hypothetical genome downsizing experienced by this genus with respect to other Tulipeae genera (Carta & Peruzzi, 2016 and literature cited therein). According to the latter authors, open and xeric habitats (typical of large areas of southwestern Asia) counter‐select indeed against large genome sizes.

### 4.2 Early migration from southwestern Asia

**Figure 6:**
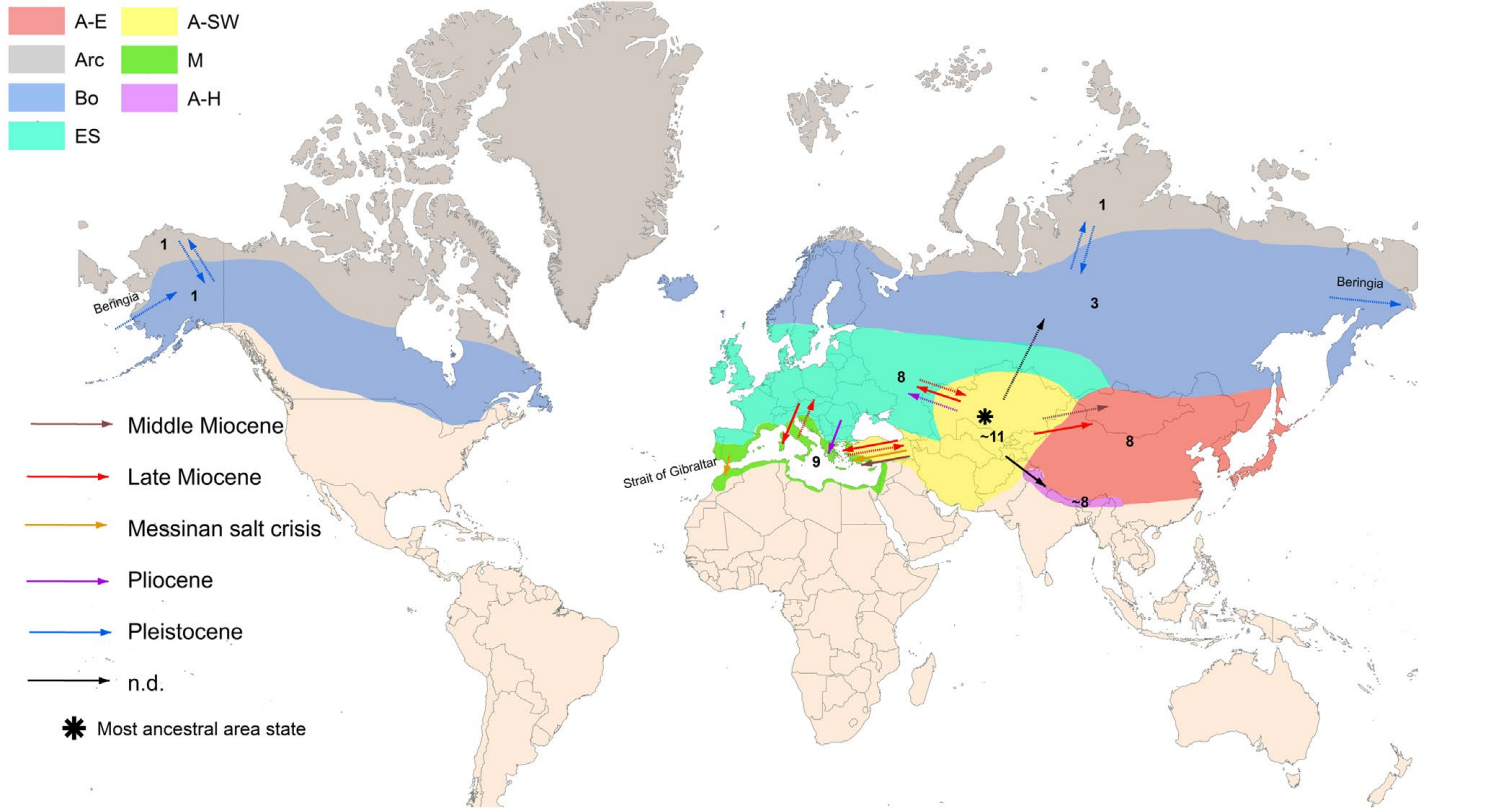
Biogeographic history of *Gagea*. The putative ancestral area is marked by *. Each phytogeographic unit is indicated by a different color code. Putative migration routes are indicated by arrows. Different colors of the arrows indicate the time and intensity of migration (continuous line: main migration route; dashed line: migration route for a limited number of lineages). Migration routes via land bridges (Beringia, Strait of Gibraltar) are indicated. In addition, for all phytogeographic units the number of currently occurring *Gagea* sections is provided. A‐E: East Asia; A‐H: Himalaya; A‐SW: southwestern Asia; Arc: Artic; Bo: Circumboreal; ES: Euro‐Siberia; M: Mediterranean

Based on the age of individual clades and ancestral area reconstruction, several migration routes can be hypothesized (Figure 6). Starting from the Middle Miocene, the Mediterranean area and East Asia were colonized from southwestern Asia, while the Euro‐Siberian area was colonized later in the Late Miocene. According to our data (ITS data, Figure 3), within all the sections originated in southwestern Asia lineages afterward diversified in the Mediterranean and Euro‐Siberian region (Late Miocene: *G*. sections *Didymobulbos*, *Gagea*, *Platyspermum,* and *Plecostigma*) as well as in East Asia (Late Miocene: *G*. sect. *Plecostigma*; Pliocene: *G*. sections *Minimae* and *Stipitatae*). The hypothesis of a more recent colonization is also congruent with the position of several accessions from Mediterranean, Euro‐Siberia, and East Asia PUs within the combined BA tree (ITS + cpDNA; Figure 4) and at the tips of the cpDNA haplotype network (Figure 5).

In our study, accessions from Himalaya are underrepresented. Nevertheless, we argue that colonization of this region most probably started from southwestern Asia and that further migrations were blocked by geographical barriers in the northeast (Tibet Plateau: Early Oligocene; Renner, 2016) and by climate conditions (wet subtropical–tropical climate) in the east and south.

According to the ITS data, we hypothesize for some species a migration route across Euro‐Siberia, Mediterranean, and southwestern Asia in the Late Miocene, caused by similar climatic conditions and by the absence of geographical barriers. This is testified by a few species in the genus showing wide distribution ranges, as for instance *Gagea fragifera* of *G*. sect. *Didymobulbos* (Peruzzi et al., 2011).

Levichev (1999) postulated that *Gagea* came to East Asia, which is species‐poor, either from southwestern Asia or from the Siberian region. Our analysis showed that colonization of East Asia took place from southwestern Asia and Euro‐Siberia. Within *G*. sect. *Gagea*, there are two species endemic to East Asia, namely *G. terraccianoana* and *G. nakaiana* (Levichev, 1999), but they are found at different positions in our ITS phylogeny. Whereas *G. nakainana* diversified in East Asia during the Late Miocene and is sister to a group mostly consisting of species from Euro‐Siberia, *G. terraccianoana* split earlier in the Middle Miocene and is sister to all other investigated *G*. sect. *Gagea* members. This is also congruent with the cpDNA data (Figure 5), where samples of this species show haplotypes putatively ancestral to this section.

In general, this study confirms the important role of the IT region as an ancestral area and source of the flora of the neighboring regions of Euro‐Siberia, the Mediterranean, and East Asia (e.g., Manafzadeh et al., 2017). Radiation and migration out of southwestern Asia were mainly driven by Miocene climate changes (e.g., Zachos, Pagani, Sloan, Thomas, & Billups, 2001) creating drier and open habitats.

### 4.3 Pleistocene speciation, diversification, and migration to North America

Climatic oscillation during the Pleistocene were postulated to be drivers for speciation processes (Comes & Kadereit, 1998), which are also clearly evident in *Gagea*, where the majority of currently existing species had a Pleistocene origin.

This also applies to the representatives of the species‐poor *G*. sections *Anthericoides, Lloydia*, and *Spathaceae*. Our data suggest that they underwent a bottleneck with only one surviving lineage each, and diversified again during the Pleistocene. For none of these sections, a southwestern‐Asian origin was inferred. The ancestral area for the common ancestor of *G*. sections *Anthericoides* and *Lloydia*, which are sister groups, most likely was the Mediterranean or Euro‐Siberia.

The clonal species *Gagea spathacea*, the only representative of *G*. sect. *Spathaceae*, occurs in central (incl. southern Scandinavia) and southeastern Europe, but also in the western Colchis floristic region (A‐SW). Levichev et al. (2010) hypothesized a Late Miocene A‐SW origin for this species, which is not contradicted by our analysis. Pfeiffer et al. (2012) considered the Colchis population as putatively relictual, which is not reflected in here. However, a population genetics approach (e.g., genome‐wide single‐nucleotide polymorphism data) and a thorough sampling would be necessary to determine in which PU the most ancestral populations of *G. spathacea* occur.

We assume that during the Pleistocene, some *Gagea* species migrated northwards to the Circumboreal PU. Only four species occur there: *G. granulosa* Turcz. and *G. minima* (L.) Ker Gawl. (*G*. sect. *Minimae*), *G*. *fragifera*, and *G*. *serotina* (Meusel et al., 1965). Although we did not include accessions of these species from the Circumboreal PU, with the exception of *G. serotina* (*G*. sect. *Lloydia*), it is plausible that colonization of northern Asia started from Euro‐Siberia also for the other three species.

The only species in the genus which colonized northwestern America is *Gagea serotina*, also inhabiting Europe and northern Asia (Peterson et al., 2008). The colonization of northwestern America presumably happened during the Pleistocene via the Beringia land bridge, which connected East Asia and North America during glacial periods. At the Pliocene/Pleistocene boundary, the vegetation type of Beringia mostly comprised boreal forest‐tundra (Graham, 2018), so that migration from Asia was possible for cold‐tolerant species (Edwards, Lloyd, & Armbruster, 2018) such as *G. serotina* (Peruzzi, Tison, et al., 2008). The role of Beringia for the colonization of North America was discussed for several plant groups (tribe Lilieae: Huang et al., 2018; main *Arabis* clade of tribe Arabideae of the Brassicaceae: Karl & Koch, 2013; for further examples see Graham, 2018; Maguilla, Escudero, & Luceño, 2018). The flat seeds of *Gagea serotina* are adapted to dispersal by wind. This effective dispersal is possibly the cause for the large range with disjunct occurrences. Therefore, it cannot be ruled out that *G. serotina* arrived to Northern America through long‐distance dispersal, without involving land bridges. This was also discussed, for example, for *Carex* section *Glareosae* (Maguilla et al., 2018).

### 4.4 The colonization of the Mediterranean resulted in a secondary center of diversity

It is generally admitted that Irano‐Turanian elements could colonize the eastern Mediterranean region via land bridges during the Early‐to‐Late Miocene or later in the Pleistocene (Manafzadeh et al., 2017), while the western Mediterranean was colonized via land bridges either in the Oligocene/Miocene or later during the Messinian Salinity Crises (5.96–5.33 Ma; Duggen, Hoernle, Bogaard, Rupke, & Morgan, 2003). According to our analyses, the Mediterranean region was repeatedly colonized in the Miocene, and later during the Messinian Salinity Crises, by *G*. sect. *Didymobulbos*, that shows its highest diversity in this region (Tison et al., 2013). Most of the currently existing Mediterranean *G*. sect. *Didymobulbos* species originated during the last 3 Ma, suggesting that the onset of the Mediterranean climate (3.4–2.8 Ma) played an important role in speciation and diversification processes, in agreement with other studies (for examples in plants, see Fiz‐Palacios & Valcárcel, 2013).

According to the ITS data set and the combined data set (ITS and cpDNA) and to the position of North African accessions at the tips of the cpDNA network, it seems that *G*. sect. *Didymobulbos* colonized North Africa from northern parts of the Mediterranean, presumably via a land‐bridge connection between Iberia and North Africa, during the Messinian Salinity Crises, before the (re)establishment of the Strait of Gibraltar (5.3 Ma; e.g., Rodríguez‐Sánchez, Pérez‐Barrales, Ojeda, Vargas, & Arroyo, 2008).

A similar pattern was observed in the geophyte lowland *Narcissus* subg. *Hermione*, where the colonization of the Mediterranean is supposed to be influenced by different factors like land‐bridges between Africa and Iberia, the Messinian salinity crisis and Pleistocene climate oscillations (Santos‐Gally, Vargas, & Arroyo, 2012).

However, seemingly the Mediterranean was also colonized from the Euro‐Siberian PU. A large subclade of *G*. sect. *Gagea* (GAG IIb; Figures 3 and 4) diversified in Euro‐Siberia during the Pliocene (Figure 3). The few Mediterranean samples, nested within the latter subclade, split relatively late. Although European and Mediterranean species are quite well represented in our study, it is important to note that we only included about 41% of the species of *G*. sect. *Gagea*, which is characterized by a high amount of endemism (Peterson et al., 2016). Also the cpDNA network (Figure 5) is characterized by about 30% of missing haplotypes, confirming a gap in our sampling.

Unclear is how the ancestor of *G* sect. *Anthericoides* (3.2 –0.4 Ma) colonized the Mediterranean. This section is endemic to this area (Peruzzi, Tison, et al., 2008) and includes only two species. Whereas *G. graeca* is restricted to the northeastern Mediterranean (Schnittler et al., 2017), *G. trinervia* inhabits the Central Mediterranean, that is, Libya and Sicily (Peruzzi, Tison, et al., 2008).

The Mediterranean can be considered as a secondary center of speciation for *Gagea* (e.g., Peterson et al., 2009). A high degree of dispersal from IT floristic region to the Mediterranean was shown in some other phytogeographical studies (e.g., Apiaceae: Banasiak et al., 2013; *Haplophyllum* (Rutaceae): Manafzadeh et al., 2014). However, particularly for *G*. sect. *Didymobulbos*, an early colonization (Miocene) of the Mediterranean might be responsible for the high species diversity of this section in this area.

## 5 CONCLUSION

Our study demonstrates that the species‐rich IT floristic region, a current center of *Gagea* species diversity, is also the center of origin of this monocot genus and of its major linages. This supports our hypothesis (*H1*) that the IT region is an important source of taxa for adjacent areas of Euro‐Siberia, the Mediterranean and East Asia, since the colonization of these areas started from the IT floristic region. The high species diversity of *G*. sect. *Didymobulbos* in the Mediterranean could be explained by an early colonization of this region, followed by extensive in situ speciation, also in this case supporting our starting hypothesis (*H2*). Miocene climate changes created open and dry habitats and allowed *Gagea* to colonize regions adjacent to southwestern Asia. During the Pleistocene, land bridges seemingly played an important role for the migration to northwestern Africa (strait of Gibraltar) and to North America (Beringia), although long‐distance dispersal events cannot be excluded. *Gagea* includes only a single widespread species (*G*. *serotina*) that was able to colonize the Arctic and America. Thus, periods of climatic changes played an important role for colonization processes, in agreement with our third hypothesis (*H3*). Accordingly, geographical and climatic barriers contributed to the current distribution and to the high degree of stenochory in the genus.

The most likely model for ancestral area reconstruction for both the ITS data set and the combined data set (ITS + cpDNA) was DEC+J, accounting for founder events, resulting in genetically isolated populations evolving independently. It is usually reported for island, and often criticized (Matzke, 2014). In *Gagea*, species are indeed often geographically isolated, growing on distinct, separated mountain ranges. Many species are also reproductively isolated by different ploidy levels or propagate mainly vegetatively (e.g., Tison et al., 2013). It is also important to highlight that all models tested (with the exception of one model in the combined data set) eventually estimated southwestern Asia as the most likely ancestral area for *Gagea* (Tables 6 and 8) and for its major lineages. Since the probabilities for ancestral ranges are influenced by those of parental nodes, as well as by those of derived nodes, we assume that the inclusion of outgroups from A‐SW would have resulted in an even higher probability for a southwestern‐Asian origin of the genus.

However, further plant case studies dealing with IT elements, to analyse their temporal and spatial relationships to neighboring floristic regions, are needed.

In contrast to other studies, where a single accession is assigned to all PUs for which a species is recorded (e.g., Maguilla et al., 2018), in our study each accession was assigned to its PU of provenance. Such an approach is crucial in a species‐rich, biologically complex, and taxonomically difficult genus as *Gagea*.

To avoid a reduction of the number of accessions (517 to 399) and PUs (six to five), our reconstruction of the evolutionary history of the genus was based only on the nrITS region. However, the reduced data set (77%) of combined sequences (ITS + cpDNA) produced only a few incongruences. The few observed incongruences are more likely a result of the different tools used for inferring the phylogenies, since, for example, the position of *G*. section *Plecostigma* and the one sequence type of *G*. sect. *Stipitatae* (*G. caelestis* and *G. pseudominutiflora*) was already agreed with that of earlier studies (ITS as well as ITS + cpDNA trees; Peterson et al., 2008). Although intersectional hybridization is not proved so far within *Gagea* (see also Introduction), we cannot rule out that nondetected hybridization processes could have influenced our analyses. Concerted evolution can cause the maintenance of just one parental ribotype, which is one of the reasons why the inference of relationships in phylogenies can be misleading (Gehrke, Martín‐Bravo, Muasya, & Luceño, 2010). The same applies to uniparentally inherited plastid markers, where incomplete linage sorting can play a role (Jakob & Blattner, 2006). However, there is still need to find further appropriate molecular markers to obtain a fully resolved backbone for *Gagea*.

Despite this, our study summarizes the current phylogenetic knowledge about this genus and highlights which sections (*Anthericoides*, *Didymobulbos*, *Lloydia*, *Minimae*, *Persicae*, *Spathaceae, Triflorae*) are well investigated and which sections (*Gagea*, *Platyspermum*, *Plecostigma*, *Stipitatae*) are still in need of additional phylogenetic and biogeographical studies. For the latter four sections in particular, the inclusion of more samples from the Himalaya would be useful.

Taxonomic conclusions on sectional circumscription, for example, concerning *G*. sect. *Stipitatae,* or concerning the classification of “*tib*” and “*yun*” are certainly premature and would deserve further morphological and molecular investigations. Also at species level (e.g., several taxa within *G*. sect. *Gagea*) more studies are necessary. Nevertheless, these problems are far out of the scope of this paper.

## CONFLICT OF INTEREST

None declared.

## AUTHOR CONTRIBUTIONS

AP, DH, and JP conceived the idea; AP, DH, JP, and LP conducted the fieldwork and collected the material with additional material from collaborators; AP carried out the laboratory work. AP (sequence data) and DH (ancestral area and network construction) analyzed the data. AH prepared the distribution map. AP and DH drafted and revised the manuscript with the assistance from all other coauthors. All authors approved the submission.

## Data Availability

All DNA sequence data are deposited in the European Nucleotide Archive (ENA). For accession numbers see Appendix Table S1. The Appendix including Table S1 and Figures S1‐S4 can be accessed under https://doi.org/10.5061/dryad.97np7bt.
