# Supplementary material for: A pre‐Miocene Irano‐Turanian cradle: Origin and diversification of the species‐rich monocot genus Gagea (Liliaceae)
Source: Ecol Evol. 2019 Apr 26;9(10):5870–90. doi: 10.1002/ece3.5170 (PMC6540665; doi:10.1002/ece3.5170)

APPENDIX

Table S1. Sources of the studied material (voucher, origin and references), cpDNA haplotype (HT) and ITS type and Gene bank accession numbers (European Nucleotide Archive) for *psb*A–*trn*H IGS, *trn*L-*trn*F IGS and ITS region: ITS1+5.8SrDNA+ITS2. Information about distribribution of the studied species.

Section / *Species*[a]	Distribution[b]	Origin	Region	cpDNA HT	ITS-type	Voucher	Gene bank Acc. No. *psb*A-*trn*H IGS	*trn*L-*trn*F IGS	ITS	Coordinates[c] NORTH	EAST	WEST	References[d]
Anthericoides **A.Terracc.**													
G. graeca (L.) Irmsch.	Mediterranean*	Greece: Crete, Lassithi Plateau, Kera	M	ANT-3	ant-2	HAL 099962	AM049263	AJ810090	AJ810089	35°28'11''	23°43'34''		Peruzzi et al., 2008b
		Greece: Crete, Levka Ori Mts	M	ANT-2	ant-1	HAL 119722	LT985395	LT985679	LT985640	35°24'33''	24°04'16''		this study
		Greece: Crete, Ori Thriptis	M	ANT-2	ant-2	HAL 105185	AM939648	AM939645	AM939649	35°05'	25°50'		Peruzzi et al., 2008b
		Greece: Crete, Plakias, Koroupu hills	M	ANT-1	ant-1	HAL 119717	LT985394	LT985678	LT985639	35°13'37''	24°24'10''		this study
		Greece: Crete, Psilioritis Mts., Anogia	M	ANT-1	-	HAL 119723	LT985393	LT985677	-	35°17'58''	24°55'36''		this study
		Greece: Crete	M	ANT-2	ant-2	HAL 099936	AM939647	AM939646	AM939650	35°08'	25°33'		Peruzzi et al., 2008b
		Greece: Delos	M	ANT-4	-	Davis 40591 (K) (Kew 23339)	EU939285	EU912316	-	37°23'	25°16'		*Zarrei et al., 2009*
		Greece: Lakonia	M	ANT-2	ant-3	B 100073942/Mus. Bot. Berol.92.398	AM779097	AM779096	AJ810088	37°03'	22°52'		Peterson et al., 2008
G. trinervia (Viv.) Greuter	Mediterannean*	Italy: Sicily	M	ANT-5	ant-4	CLU 19785	AM409356	AM409359	AM779093	37°15'	14°39'		Peruzzi et al., 2008a
		Libya: Cirenaica el-Beda, Sidi Rafa	M	ANT-5	-	6/IV/1933 Pampanini (FI)	AM779094	AM779095	-	32°45'0''	21°43'60''		Peruzzi et al., 2008b
Bulbiferae **Levichev**													
G. bulbifera (Pall.) Salisb.	East Asian, European, Siberia,South-west Asian, Western Tien Shan*	Azerbaijan: nr. border of Iraq, Dizaj	A-SW	BUL-2	bul-2	TUH-E BOT.EXP. 35713 (TUH) (Kew 23140)	EU939226	EU912254	EU912027	37°09'			
		China: Xinjiang, Urumqi,Yamalik Hill Forest Park	A-E	BUL-1	bul-1	HAL107953	FR690829	FR690234	FR689753	43°48′00″	87°34′51''		*Zarrei et al., 2009*
		Iran: Ardabil	A-SW	BUL-3	bul-3	Zarrei & al. TUH-E BOT.EXP. 35709 (TUH) (Kew 23166)	EU939227	EU912255	EU912028	38°15'14''	48°17'60''		*Zarrei et al., 2009*
		Kazakhstan: Almaty, S Kapchagay	A-SW	BUL-1	bul-1	HAL 118024	LT985396	LT985706	LN874788	43°44'24''	77°01'16''		Peterson et al., 2016; this study
		Kazakhstan: Almaty, S Kapchagay	A-SW	BUL-1	bul-1	HAL 118026	LT985397	LT985707	LN874789	43°44'18''	77°00'43''		Peterson et al., 2016; this study
		Kazakhstan: Ili-Alatau range, Almaty, Medeu statium	A-SW	BUL-1	bul-1	HAL 118081	LT985398	LT985708	LN874790	43°10'12''	77°04'42''		Peterson et al., 2016; this study
		Kazakhstan: Ili-Alatau range, Almaty, Medeu statium	A-SW	BUL-1	bul-1	HAL 119676	LT985399	LT985709	LN874791	43°10'12'''	77°04'42'		Peterson et al., 2016; this study
		Kazakhstan: Ili-Alatau range, Almaty, S Turar	A-SW	BUL-1	bul-1	HAL 118028	LT985400	LT985710	LN874793	43°14'58''	76°32'13''		Peterson et al., 2016; this study
		Kazakhstan: Ili-Alatau range, Almaty, S Turar	A-SW	BUL-1	bul-1	HAL 118030	LT985401	LT985711	LN874794	43°13'15''	76°33'10''		Peterson et al., 2016; this study
		Russia: District Astrakhan	ES	BUL-1	bul-1	HAL 101840	AM049260	AJ969119	AM162669	47°11'	47°09'		Peterson et al., 2008
G. rufidula Levichev	South-west Asian, Western Tien Shan*; China: Xinjiang, Eastern Tian-Shan*	China: Xinjiang,Tian-Shan, Bogda-Shan, Tianchi Lake	A-E	BUL-4	bul-4	HAL 107929	FR690840	FR690245	FR689764	43°53′38''	88°07'02''		Peterson et al., 2011
		China: Xinjiang,Tian-Shan, Bogda-Shan, Tianchi Lake	A-E	BUL-4	bul-4	HAL 107924	FR690841	FR690245	FR689765	43°53′31''	88°06′02''		Peterson et al., 2011
		China: Xinjiang,Tian-Shan, Bogda-Shan, Tianchi Lake	A-E	BUL-4	bul-4	HAL 107922	FR690842	FR690247	FR689766	43°54′27''	88°06'46''		Peterson et al., 2011
		Kyrgyzstan	A-SW	BUL-4	bul-5	149/09DNALevichev (LE)	FR690843	FR690248	FR689767	42°30′	74°35'		Peterson et al., 2011
Didymobulbos **(K.Koch) Boiss.**													
G. algeriensis Chabert	Morocco, Algeria**	Morocco: Oujda, Jerada	M	DID-1	did-1	HAL 108486	HE649692	HE649596	HE649505	34°41'		01°54'	Tison et al., 2013
G. amblyopetala Boiss. & Heldr.	Italy, Sicily, Greece, Aegean islands**	Greece: Evritania - Gardiki	M	DID-2	did-2	HAL 107892	HE649693	HE649597	HE649506	38°59'	21°43'		Tison et al., 2013
		Greece: Evvoia - Steni: à proximité du col des Monts Dirphys	M	DID-2	did-3	HAL 107891	HE649694	HE649598	HE649507	38°34'	23°49'		Tison et al., 2013
		Italy: Sicily, Iblei, Monte Lauro	M	DID-2	did-2	HAL 107890	HE649695	HE649599	HE649508	37°12'	14°48'		Tison et al., 2013
G. apulica Peruzzi & J.-M.Tison	France, Italy, Sicily**	France: Martigues: plateau de Bonnieu	M	DID-3	did-4	HAL 107902	HE649696	HE649600	HE649509	43°24'	05°03'		Tison et al., 2013
		Italy: Apulia, Lesina	M	DID-3	did-4	CLU 12697	AM409348	AM283111	AM903046	41°51'	15°21'		Tison et al., 2013
		Italy: Apulia, Murge: S. Simone, Taranto	M	DID-3	did-1	CLU 18172	HE649697	HE649601	HE649511	40°37'	17°15'		Tison et al., 2013
		Italy: Sicily, Campobello di Mazara, Trapani	M	DID-3	did-4	PI *s.n.*	HE649699	HE649603	HE649512	37°37'	12°44'		Tison et al., 2013
		Italy: Sicily, San Vito Lo Capo, Trapani	M	DID-4	did-1	PI *s.n.*	HE649700	HE649604	HE649513	37°37`	12°44'		Tison et al., 2013
G. bohemica (Zauschn.) Schult. & Schult.f.	Europe, Algeria; Near East?**	Czech Republic: Kamenište	ES	-	did-6	DANIHELKA s.n. (STU)	-	-	FR874906	50° 47'	14° 25'		*Wörz et al., 2012*
		Czech Republik: Moravia, Ivančice near Brno	ES	DID-5	did-5	HAL 095852	AJ416370	AJ419161	AJ427549	49°05'	16°23'		Peterson et al., 2004
		Germany: Rhineland-Palatinate, Muenster-Sarmsheim	ES	DID-6	did-8	HAL 095855	LT985404	FM204907	AJ437199	49°56'	7°53'		Peterson et al., 2004, 2008, 2010
		Germany: Rhineland-Palatinate, Neu-Bamberg, Haarberg	ES	DID-5	-	HAL 095856	LT985405	FM204922	-	49°48'	7°55'		Peterson et al., 2004, 2010
		Germany: Saxony-Anhalt, Ostrau	ES	DID-6	did-7	HAL 095849	AJ416371	FM204911	AJ437198	51°37°	12°1'		Peterson et al., 2004, 2008, 2010
		Greece	M	DID-6	-	GAT 34674	LT985402	LT985712	-	37.574869	26.060942		this study
		Greece: Crete, near Ideon Andron	M	DID-5	did-6	HAL 101645	AM161463	AM161469	AM265528	35°12'	24°50'		Peterson et al., 2008, 2010
		Greece: Crete, Psiliorithis Mts.	M	DID-7	-	HAL 119726	LT985403	LT985713	-	35°13'07''	24°49'00''		this study
		Greece: Crete, Psiliorithis Mts.	M	DID-35	-	HAL 119724	LT985426	LT985806	-	35°12'37''	24°49'28''		this study
		Italy: Abruzzo, Gran Sasso	M	DID-6	did-9	CLU 14925	AM282987	AM265600	AM287271	42°19'	13°35'		Peruzzi et al., 2008a; Peterson et al. 2010
		Russia: Dagestan	ES	DID-5	did-10	HAL 101787	AM085142	AJ969117	AM287272	42°59'	47°29''		Peterson et al., 2008
		Spain: Orihvela del Tremedal/Tervel	ES	DID-5	did-11	HAL 107869	HE649701	HE649605	AM287273	40°33'		01°39'	Peruzzi et al., 2008a; Tison et al., 2013
		United Kingdom: Wales, Stanner Rocks, Radnorshire	ES	DID-5	-	-	AM265595	AM265526	AM287276	52°13'03.4"		3°04'53.8"	Peruzzi et al., 2008a
		Turkey: W, near Yatagan	M	DID-5	did-12	HAL 108495	HE649702	HE649606	AM287274	37°19'	28°08'		Peruzzi et al., 2008a; Tison et al., 2013
		Turkey: W, near Milas	M	DID-6	did-12	HAL 108494	HE649703	HE649607	AM287275	37°18'	27°47'		Peruzzi et al., 2008a; Tison et al., 2013
G. chabertii A.Terracc.,	Algeria**	Algeria: Tlemcen – Parc national de Tlemcen: Tizi-n-Hafir	M	DID-3	did-13	HAL 107954	HE649706	HE649610	AM287277	34°52'		01°18'	Peruzzi et al., 2008a; Tison et al., 2013
G. chrysantha Schult. & Schult.f.	Sicily**	Italy: Sicily, Ficuzza	M	DID-2	did-14	CLU 9237	AM409353	AM283110	AM287278	37°52'	13°22'		Peruzzi et al., 2008a
G. cossoniana Pascher	Spain, Morocco**	Morocco: Marrakech - Ait Barka	M	DID-8	did-15	HAL 107915	HE649689	HE649593	AM287279	31°36'		07°58'	Peruzzi et al., 2008a; Tison et al., 2013
		Morocco: Meknes - Ifrane	M	DID-1	did-16	HAL 107914	HE649690	HE649594	AM287280	33°54'		05°33'	Peruzzi et al., 2008a; Tison et al., 2013
		Spain: Malaga, Ronda	M	DID-1	did-15	HAL 107851	HE649691	HE649595	AM287281	36°43'		05°09'	Peruzzi et al., 2008a; Tison et al., 2013
G. dubia A.Terracc.	Crete, Turkey, Crimea**	Greece: Crete Hania - Omalos: secteur de Kallergi	M	DID-9	did-17	HAL 107865	HE649765	HE649668	AM287282	35°27'	24°04'		Peruzzi et al., 2008a; Tison et al., 2013
		Turkey: Central Taurus (Isauria), Akseki district	M	DID-10	-	PI *s.n.*	HE649762	HE649665	AM287283	36°58'	31°46'		Peruzzi et al., 2008a; Tison et al., 2013
		Turkey: Western Taurus (Lycia), Thermessos National Park	M	DID-11	did-18	PI *s.n.*	HE649763	HE649666	AM287284	37°00'	30°26'		Peruzzi et al., 2008a; Tison et al., 2013
		Turkey: SW-Turkey, S-Babadag	M	DID-12	did-19	HAL 108498	HE649764	HE649667	AM287285	37°48'	28°51'		Peruzzi et al., 2008a; Tison et al., 2013
		Turkey: SW Türkei, Ak dag, Gömbe	M	DID-13	-	HAL 108496	HE649761	HE649664	AM287286	37°41'	28°55'		Peruzzi et al., 2008a; Tison et al., 2013

G. durieui Parl.	Spain, Baleares, Morocco, Algeria**	Algeria: Sidi-Bel-Abbes – Sidi-bel-Abbès	M	DID-14	did-20	HAL 108493	HE649714	HE649618	HE649526	35°11'		00°38'	Tison et al., 2013
		Morocco: Oujda - Taforalt: col de la S403 au N du village	M	DID-14	did-20	HAL 107863	HE649711	HE649615	HE649523	34°41'		01,54'	Tison et al., 2013
		Spain: Granada – Velez-de-Benaudalla: colline dominant le village	M	DID-14	did-20	HAL 107864	HE649712	HE649616	HE649524	36°49'		03°30'	Tison et al., 2013
		Spain: Baleares, Mallorca – Llucmajor: entre Son Granada Nou, Puigderros d'Alt et Puigderros de Baix	M	DID-14	did-20	HAL 108492	HE649713	HE649617	HE649525	39°29'		02°53'	Tison et al., 2013
G. elliptica (A.Terracc.) Prain	Spain, Morocco; Portugal**	Portugal: Rocha de Pena, Salir, Algarve	M	DID-1	-	HAL 117636	LT985406	LT985714	-	37°26'		08°09'	this study
		Spain: Grazalema: à l'est du Puerto de las Palomas	M	DID-1	did-21	HAL 107861	HE649715	HE649619	HE649527	36°45'		05°21'	Tison et al., 2013
		Spain: Jubrique: au N du Puerto de Penas Blancas	M	DID-1	did-22	HAL 107860	HE649716	HE649620	HE649528	36°33'		05°12'	Tison et al., 2013
G. foliosa (C.Presl) Schult. & Schult.f.	Sardegna, Sicily, Algeria**	Algeria: Bouira – Tikjda	M	DID-15	did-23	HAL 107852	HE649717	HE649621	HE649529	36°27'	04°03'		Tison et al., 2013
		Italy: Sardinia, Oliena: montée au Sopramonte	M	DID-15	did-23	HAL 107854	HE649718	HE649622	HE649530	40°12'	09°21'		Tison et al., 2013
		Italy: Sardinia, Oliena, col Corrasi	M	DID-15	did-23	HAL 107853	HE649719	HE649623	HE649531	40°16'36''	09°24'7''		Tison et al., 2013
		Italy: Sardinia, Orani	M	DID-15	-	HAL 104326	AM903063	AM903066	-	40°14'	09°10'		Peruzzi et al., 2008a
		Italy :Sardegna	M	DID-15	did-24	Z 34697	AM265596	AM265523	AM162676	40°02	09°14		Peterson et al., 2008
		Italy: Sicily, Madonie	M	DID-15	did-25	CLU 12691	AM282993	AM265603	AM409346	37°54'	14°01'		Peruzzi et al., 2008a
		Italy: Sicily, Petralia Sottana	M	DID-15	did-23	HAL 107855	HE649720	HE649624	HE649532	37°48'	14°05'		Tison et al., 2013
G. fragifera (Vill.) Ehr.Bayer & G.López	Europe, Sibiria, W. & C. Asia, Morocco**; China: Xinjiang, Eastern Tian-Shan*	Azerbaijan: nr boarder of Iraq	A-SW	DID-16	did-29	TUH-E BOT.EXP. 35711 (TUH) (Kew 23171)	EU939244	EU912274	EU912046	37°09'026	44°52'58''		*Zarrei et al., 2009*
		Bulgaria: Vichren (Pirin)	ES	DID-16	did-26	HAL 070407	AJ973158	AJ890368	AM162677	41°46'	23°24'		Peterson et al., 2008
		China: Xinjiang, Tian-Shan ,Bogda-Shan, Tianchi Lake	A-E	DID-16	did-27	HAL 107898	FN868198	FN868226	FN868162	43°50'54''	88°09'47''		Peterson et al., 2011
		China: Xinjiang, Tian-Shan, Bogda-Shan, Tianchi Lake	A-E	DID-16	did-28	HAL 107896	FN868199	FN868227	FN868163	43°50'17	88°10'15''		Peterson et al., 2011
		China: Xinjiang, Tian-Shan, Bogda-Shan, Tianchi Lake	A-E	DID-16	did-28	HAL 107894	FN868200	FN868228	FN868164	43°58'10''	88°05'28''		Peterson et al., 2011
		France: Alpes-Maritimes, Saint-Dalmas-le-Selvage	ES	DID-16	did-29	HAL 104325	FN868197	FN868223	FN868165	44°17'	06°51'		Peruzzi et al., 2011
		France: S France, versante meridionale di Col de la Cayolle	ES	DID-16	did-29	PI *s.n.*	FN868201	FN868224	FN868166	44°15'	06°44'		Peruzzi et al., 2011
		Iran: Ardabil, Sarein, Sabalan Mts.	A-SW	DID-17	did-29	TUH-E BOT.EXP. 35307 (TUH) (Kew 23294)	EU939278	EU912309	EU912086	38°11'	47°53'		*Zarrei et al., 2009*
		Iran: NW Ardabil, Asalem	A-SW	DID-18	did-29	Zarrei 35820 (TUH) (Kew 23144)	EU939243	EU912273	EU912045	37°35`	48°40`		*Zarrei et al., 2009*
		Iran: Tehran, Tochal Mts	A-SW	-	did-30	Zarrei & Zarrei 35265 (K, TUH) (Kew 23296)	-	-	EU912047	35°41'21''	51°23'20''		*Zarrei et al., 2009*
		Italy: Calabria, Verbicaro-Orsomarso mountains	M	DID-16	did-31	CLU 12693	AM282994	AM283103	AM422468	39°50'	16°03'		Peruzzi et al., 2008a
		Italy: Piemonte, Valle di Bellino (Cuneo), sopra Rif. Melezè	ES	DID-16	did-32	PI *s.n.*	FN868202	FN868225	FN868167	44°34'	06°59'		Peruzzi et al., 2011
		Italy: Dolomiti di Brenta, Malga Massodi	ES	DID-16	did-34	PI *s.n.*	FN868195	FN868221	FN868160	46,16328	10,91596		Peruzzi et al., 2011
		Italy: Dolomiti di Brenta, Malga Massodi	ES	DID-16	did-29	PI *s.n.*	FN868196	FN868222	FN868161	46,16328	10,91596		Peruzzi et al., 2011
		Italy: Sardegna, tra Bruncu Spina e Genne Erbeghe	M	DID-16	-	PI *s.n.*	LT985407	LT985767	-	40,01997	9,29902		this study
		Italy: Sicily, Madonie	M	DID-19	did-33	CLU 12692	AM282995	AM283102	AM287285	37°54'	14°01'		Peruzzi et al., 2008
		Kazakhstan	A-SW	DID-16	did-35	HAL 101796	AM238521	AM161467	AM180455	48°29'	84°51'		Peterson et al., 2008
		Kazakhstan: Mountains over Almaty	A-SW	-	did-35	DNA IPKKZ124	-	-	LN874804	43°03'27'	76°58'18''		Peterson et al., 2016
		Kazakhstan: Ili-Alatau range, Almaty, Medeu stadium	A-SW	DID-16	did-29	HAL 118018	LT985411	LT985771	LN874802	43°10'09''	77°02'25''		Peterson et al., 2016; this study
		Kazakhstan: Ili-Alatau range, Almaty, Shymbulak ski ressort	A-SW	DID-16	did-29	HAL 118015	LT985409	LT985769	LN874801	43°06'44''	77°04'26''		Peterson et al., 2016; this study
		Kazakhstan: Ili-Alatau range, Almaty, Shymbulak ski ressort	A-SW	DID-16	did-29	HAL 118013	LT985412	LT985772	LN874800	43°08'30''	77°03'51''		Peterson et al., 2016; this study
		Kazakhstan: Ili-Alatau range, Lake Bol. Almatinskoje	A-SW	DID-19	did-35	HAL 118032	LT985410	LT985770	LN874803	43°03'22''	76°58'05''		Peterson et al., 2016; this study
		Kazakhstan: Tenis-Korgalshinsky Nature Reserve, Astana	A-SW	DID-16	did-36	HAL 117634	LT985413	LT985773	LN874799	50°28'59''	69°32'50''		Peterson et al., 2016; this study
		Morocco: Marrakech, Oukaimeden	M	DID-16	did-32	HAL 116006	LT985408	LT985768	LN874805	31°12' 09''		07°51' 30''	Peterson et al., 2016; this study
		Switzerland: Canton Graubuenden, Jufner Alp	ES	DID-16	-	ZT 10726	AM238531	AJ890375	-	46°39'	9°34'		Peterson et al., 2008
		Ukraine: Crimea, Ai Petri	ES	DID-16	did-37	HAL 101797	AM238522	AM161466	AM265532	44°45'	34°05'		Peterson et al., 2008
G. glacialis K.Koch	Turkey**	Turkey: Rize district Ovitdagi Gecidi	A-SW	DID-20	did-38	HAL 101647	AM265586	AM287281	AM265535	40°37'31''	40°46'47''		Peterson et al., 2008
		Turkey: Goelyurt-Pass, Prov. Erzurum	A-SW	DID-16	did-40	HAL 107880	FN868207	FN868229	FN868182	38°16'	34°22'		Peruzzi et al., 2011
		Turkey: NE, Pass from Gümüshane to Kelkit	A-SW	DID-16	-	HAL 116012	LT985415	LT985775	-	40°46'	39°48'		this study
		Turkey: MeloDag, Prov. Artvin	A-SW	DID-16	did-39	HAL 107879	FN868203	FN868231	FN868178	41°05'	41°49'		Peruzzi et al., 2011
		Turkey: Ovit Pass, Prov. Rize	A-SW	DID-21	did-42	HAL 107882	FN868209	FN868235	FN868184	40°33'	40°46'		Peruzzi et al., 2011
		Turkey: Ovit Pass, Prov. Rize	A-SW	DID-20	did-43	HAL 107882	FN868210	FN868236	FN868185	40°33'	40°46'		Peruzzi et al., 2011
		Turkey: NE, Saricicek dagi	A-SW	DID-22	-	HAL 116011	LT985417	LT985777	-	40°23'	38°08'		this study
		Turkey: NE, Tersundagi-Gecidi	A-SW	DID-16	-	HAL 116013	LT985416	LT985776	-	40°31'	39°31'		this study
		Turkey: NE, Zigana-Gecidi	A-SW	DID-16	-	HAL 116010	LT985414	LT985774	-	40°37'	39°22'		this study
		Turkey: Zigana Pass, Prov. Trabzon	A-SW	DID-16	did-41	HAL 107881	FN868206	FN868234	FN868181	40°38'56''	39°23'34''		Peruzzi et al., 2011
G. granatellii (Parl.) Parl	W. Mediterranean region to Italy and Tunisia**	France: Corse, Tolla: col de Mercujo	M	DID-3	did-44	HAL 107957	HE649721	HE649625	HE649533	41°57'	08°58'		Tison et al., 2013
		France: Herault, Lacaunette	M	DID-3	did-44	HAL 107856	HE649722	HE649626	HE649534	43°21'	02°46'		Tison et al., 2013
		Italy: Apulia, Murge, S. Paolo, Taranto	M	DID-3	did-44	CLU 18184	HE649725	HE649629	HE649537	40°37'	17°15'		Tison et al., 2013
		Italy: Apulia, Murge, S. Paolo, Taranto	M	DID-3	did-44	CLU 18176	HE649726	HE649630	HE649538	40°37'	17°15'		Tison et al., 2013
		Italy: Sardinia, Sassari, Tempio Pausania	M	DID-3	did-44	HAL 104329	HE649727	HE649631	HE649539	40°54'	09°06'		Tison et al., 2013
		Italy: Tuscany, Isola del Giglio	M	DID-3	did-44	PI *s.n.*	HE649723	HE649627	HE649535	42°20'	10°54'		Tison et al., 2013
		Italy: Tuscany, Elba Island	M	DID-3	did-44	PI *s.n.*	HE649724	HE649628	HE649536	42°45'	10°12'		Tison et al., 2013
		Italy: Calabria, Sila	M	DID-3	did-44	CLU 9266	AM282988	AM265598	AM409344	39°23'	16°17'		Peruzzi et al., 2008a
		Italy: Sardinia, Orani	M	DID-3	-	HAL 104330	AM903058	AM903067	-	40°14'	09°10'		Peruzzi et al., 2008a
		Italy: Sicily, Iblei , Monte Lauro	M	DID-3	did-44	CLU 9267	AM282989	AM265599	AM409343	37°06'	14°49'		Peruzzi et al., 2008a
		Tunisie: Tabarka, arrière-plage de Barkoukech	M	DID-3	did-44	HAL 107961	HE649728	HE649632	HE649540	36°58'	08°50'		Tison et al., 2013
G. heldreichii (A.Terracc.) Stroh	Greece, Crete, Cyprus, Turkey**	Greece: Attiki, Agia Triada	M	DID-23	did-44	HAL 107857	HE649729	HE649633	HE649541	38°10'	23°43'		Tison et al., 2013
		Greece: Crete, Hania - Omalos: plaine entre le village et le col de Xyloskalo	M	DID-24	did-45	HAL 107873	HE649731	HE649635	HE649543	35°20'	23°54'		Tison et al., 2013

G. lacaitae A.Terracc.	W. Mediterranean region to Italy and Algeria**	France: Gignac: les Piélettes c. 10 m – friche au bord de l'autoroute	M	DID-25	did-46	HAL 075509	HE649732	HE649636	HE649544	43°39'	03°33'		Tison et al., 2013
		France: Herault	M	DID-25	did-47	HAL 101837	AM265592	AM265515	AM409333	43°21'	02°46'		Peterson et al., 2008; Peruzzi et al., 2008a
		France: Réauville: plateau de St-Pancrace	M	DID-25	did-48	HAL 107909	HE649733	HE649637	HE649545	44°26'	04°50'		Tison et al. ,2013
		Italy: Abruzzo, Gran Sasso	M	DID-25	did-46	CLU 14924	AM282990	AM265597	AM409342	42°19'	13°35'		Peruzzi et al., 2008a
		Italy: Sicily, Caltagirone al'ene de la ville pres du lieu dit Ogliastro	M	DID-25	did-47	HAL 107910	HE649734	HE649638	HE649546	37°15'	14°33'		Tison et al., 2013
		Spain: Malaga - Ronda: Puerto del Viento	M	DID-25	did-46	HAL 105936	HE649735	HE649639	HE649547	36°43'		05°09'	Tison et. al., 2013
		Spain: Malaga - Ronda: Puerto del Viento	M	DID-25	did-49	HAL 071229	HE649736	HE649640	HE649548	36°43'		05°09'	Tison et. al., 2013
G. lojaconoi Peruzzi	Italy, Sardegna, Sicily; N.-E. Mediterranean region?**	Italy: Apulia, Monte Sant'Angelo, Foggia, au-dessus du village sur la route du Gargano	M	DID-2	did-51	HAL 107908	HE649737	HE649641	HE649549	41°42'	15°57'		Tison et al., 2013
		Italy: Calabria, San Marco Argentano	M	DID-2	did-50	CLU 9256	AM282997	AM283106	AM287272	39°36'	16°13'		Peruzzi et al., 2008a
		Italy, Sicily,Iblei, Monte Lauro	M	DID-2	did-50	CLU 9253	AM282998	AM283107	AM422464	37°35' 59	14°0'55		Peruzzi et al., 2008a
G. luberonensis J.-M.Tison	France, Italy**	France: Ardiche, Berrias-et-Casteljau	M	DID-6	did-52	HAL 107870	HE649738	HE649642	HE649550	44°22'	04°12'		Tison et al., 2013
		France: Berrias-et-Casteljau: plateau du Gras de la Rouveyrolle	M	DID-6	did-55	HAL 107904	HE649742	HE649645	HE649553	44°22'	04°12'		Tison et al., 2013
		France: Bouches-de-Rhone, Auriol	M	DID-6	did-59	HAL 107859	HE649745	HE649648	HE649556	43°22'	5°63'		Tison et al., 2013
		France: Gard, Sommieres	M	DID-25	did-53	HAL 107871	HE649739	HE649643	HE649551	43°47'	04°05'		Tison et al., 2013
		France: Robion: au S.-E. de la bergerie du Colombier	M	DID-26	did-56	HAL 107906	HE649743	HE649646	HE649554	43°50'	05°06'		Tison et al., 2013
		France: Sommières: plateau de Fontbouisse	M	DID-25	did-57	HAL 107905	HE649744	HE649647	HE649555	43°47'	04°05'		Tison et al., 2013
		France: Taillades: pied du Petit Luberon, vallon de la Combe	M	DID-6	did-54	HAL 107907	HE649741	HE649644	HE649552	43°50'	05°05'		Tison et al., 2013
		France: Vaucluse, Cheval-Blanc	M	DID-6	-	HAL 105944	HE649740	FM200221		43°48'	05°03'		Tison et al., 2013
		Italy: Abruzzo, Gran Sasso	M	DID-6	did-58	CLU 14926	AM283000	AM283105	AM422467	42°19'	13°35'		Peruzzi et al., 2008a
		Italy: Latium, M. Tancia	M	DID-6	did-9	CLU 20581	AM903062	AM903068	AM422463	42°18'	12°44'		Peruzzi et al., 2008a
G. lusitanica A.Terracc	Spain, France; Portugal?**	Spain: Granada – Loja: N321 au pied de la montagne de Los Infiernos (900 m)	M	DID-28	did-61	HAL 108500	HE649747	HE649650	HE649558	37°10'		04°09'	Tison et al., 2013
		Spain: Grazalema: à l'est du Puerto de las Palomas	M	DID-27	did-60	HAL 107887	HE649746	HE649649	HE649557	36°45'		05°21'	Tison et al., 2013
		Spain: Malaga - Ronda: versant ouest du Puerto del Viento	M	DID-3	did-62	HAL 107911	HE649748	HE649651	HE649559	36°43'		05°09'	Tison et al., 2013
G. luteoides Stapf	Turkey; Bulgaria?**	Turkey: btwn. Penarbau and Sariz, 10km from Sariz, Demici Pass	A-SW	DID-29	did-32	Baytor, T. ISTE 44270 (K)(Kew 23280)	EU939251	EU912282	EU912053	38°29'	36°29'		*Zarrei et al., 2009*
G. mauritanica Durieu	Algeria, Baleares**	Algerine: Oran, St. Eugene	M	DID-3	-	HAL 107901	HE649749	HE649652	-	35°41'		00°37'	Tison et al., 2013
		Spain: Baleares, Mallorca, Llucmajor	M	DID-3	did-63	HAL 107903	HE649750	HE649653	HE649560	39°29'		02°53'	Tison et al., 2013
		Spain: Baleares, Mallorca, Llucmajor	M	DID-3	did-63	HAL 107900	HE649751	HE649654	HE649561	39°29'		02°53'	Tison et al., 2013
G. microfistulosa Levichev	Crimea*	Ukraine: Crimea	ES	DID-30	did-64	HAL 101728	AM265589	AM265517	AM409332	44°57'	34°06'		Peterson et al., 2008
G. moniliformis J.-M.Tison	Morocco**	Morocco: Marrakech - Oukaimeden: à la station	M	DID-1	did-65	HAL 107866	HE649752	HE649655	HE649562	31°05'		07°52'	Tison et al., 2013
G. nevadensis Boiss.	Spain, Morocco**	Spain: Granada, Monachil	M	DID-31	did-60	HAL 107888	HE649753	HE649656	HE649563	37°07'		03°32'	Tison et al., 2013
		Spain: Granada, Monachil	M	DID-31	did-66	HAL 107889 (DNA1)	HE649754	HE649657	HE649564	37°07'		03°32'	Tison et al., 2013
		Spain: Granada, Monachil	M	DID-27	did-67	HAL 107889 (DNA2)	HE649755	HE649658	HE649565	37°07'		03°32'	Tison et al., 2013
G. omalensis J.-M.Tison	Crete**	Greece: Crete Hania - Omalos: plaine entre le village et le col de Xyloskalo	M	DID-2	did-68	HAL 107877	HE649756	HE649659	HE649566	35°20'	23°54'		Tison et al., 2013
		Greece: Crete Hania- Omalos Plateau	M	DID-2	did-69	HAL 108490	HE649567	HE649660	HE649567	35°20'	23°54'		Tison et al., 2013
G. peduncularis (J.Presl & C.Presl) Pascher	Italy, Greece, Aegean islands, Crete,Turkey**	Greece	M	DID-33	did-73	GAT 34679	LT985422	LT985802	LT985642	40.657531	24.512631		this study
		Greece	M	DID-33	did-73	GAT 34676	LT985423	LT985803	LT985643	38.381844	26.073728		this study
		Greece	M	DID-33	did-74	GAT 34672	LT985424	LT985804	LT985644	38.583086	25.996572		this study
		Greece: Attiki, near Vraila (S of Lidoriki)	M	-	did-75	GAT 23065	-	-	LT985645	38°26'50,6	22°11'14,7		this study
		Greece: Fokida - Lilea: contreforts du Mont Parnasse	M	DID-32	did-70	HAL 107885	HE649758	HE649661	HE649568	38°43'	22°09'		this study
		Greece: Crete, Pano, Elounda	M	DID-33	-	HAL 118005	LT985418	LT985798	-	35°15'56''	25°42'57''		this study
		Greece: Crete, hills above Elounda	M	DID-33	did-72	HAL 118000	LT985419	LT985799	LT985641	35°8'56''	25°25'49''		this study
		Greece: East crete, Ori Thriptis, near Orino	M	DID-33	did-70	HAL 108499	HE649759	HE649662	HE649569	35°03'	25°57'		Tison et al., 2013
		Greece: Crete: Kritsa to Katharo high plain	M	DID-33	-	HAL 118001	LT985420	LT985800	-	35.16	25.64		this study
		Greece: Crete, Kritsa, Katharo high plain	M	DID-33	-	HAL 118004	LT985421	LT985801	-	35.16'	25.64		this study
		Greece: Is Rodhes, Mt. Profet Elias, Archangelos Church	M	DID-34	did-71	Davis 40349 (K) (Kew 23333)	EU939252	EU912283	EU912054	36°21'	28°12'		*Zarrei et al., 2009*
		Italy: Apulia, Murge, Taranto	M	DID-32	did-76	CLU 18163-18165	AM903056	AM409357	AM903050	40°37	17°15'		Peruzzi et al., 2008a
		Turkey: SW, Bey daglari	M	DID-33	-	HAL 116085	LT985425	LT985805	-	36°60'	30°12'		this study
G. peruzzii J.-M.Tison	Italy**	Italy: Basilicata, Pollino Massif	M	DID-35	did-78	CLU 12685	HE649704	HE649608	HE649517	39°56'	16°16'		Tison et al., 2013
		Italy: Calabria, Pollino Massif	M	DID-35	did-78	CLU 9236	HE649705	HE649609	HE649518	39°51'	16°05'		Tison et al., 2013
		Italy: Calabria, Verbicaro-Orsomarso mountains	M	DID-35	did-77	CLU 12688	AM932484	AM932485	AM931940	39°50'	16°03'		Peterson et al., 2009, 2010
G. polidorii J.-M.Tison	France, Italy**	France: Alpes-Maritimes, Saint-Dalmas-le-Selvage	ES	DID-16	did-80	HAL 107417	FN868211	FN868237	FN868187	44°17'	06°51'		Peruzzi et al., 2011
		France: Corse, Sorio: au niveau du col entre les Monte Grimaesta et Astu	M	DID-16	did-79	HAL 107416	FN868212	FN868238	FN868186	42°34'	09°16'		Peruzzi et al., 2011
		Italy: Calabria, Verbicaro-Orsomarso mountains	M	DID-16	did-81	CLU 12700	AM282999	AM283104	AM903053	39°50'	16°03'		Peruzzi et al., 2008a
G. polymorpha Boiss	Spain**	Spain: Granada – La Calahorra	M	DID-36	did-20	HAL 107913	HE649766	HE649669	HE649574	37°10'		03°03'	Tison et al., 2013
		Spain: Granada – Padul: contreforts ouest de la Sierra del Manar (1000 m)	M	DID-36	did-20	HAL 107912	HE649767	HE649670	HE649575	37°01'		03°37'	Tison et al., 2013
G. pseudopeduncularis J.-M.Tison	Greece**	Greece: Attiki - Agia Triada: crête du Mont Parnes	M	DID-37	did-82	HAL 107886	HE649760	HE649663	HE649570	38°10'	23°43'		Tison et al., 2013
G. ramulosa A.Terracc.	Spain, Morocco, Italy, Sardinia, Sicily,Greece, Turkey**	Italy: Abruzzo, Altopiano delle Rocche	M	DID-38	did-83	APP 20580	AM409355	AM409360	AM422462	42°12'	13°31'		Peruzzi et al., 2008a
		Italy: Sardinia, Supramonte	M	DID-38	did-84	HAL 104332	AM903054	AM903064	AM903049	40°12'	09°21'		Peruzzi et al., 2008a
		Spain: Granada, Monachil	M	DID-39	did-85	HAL 107868	HE649707	HE649611	HE649520	37°07'		03°32'	Tison et al., 2013
		Turkey: Central Taurus (Isauria), Ibradi district, Gembos pasture	M	DID-40	did-83	PI *s.n.* DNA1	HE649708	HE649612	HE649521	37°12'	31°27'		Tison et al., 2013
		Turkey: SW Türkei, Korkuteli-Antalya	M	DID-40	did-85	HAL 108497	HE649710	HE649614	HE649522	37°04'	30°12'		Tison et al., 2013
G. sicula Lojac.	Sicily**	Italy: Ficuzza, Palermo: sommet du Monte Cucco	M	DID-24	did-86	HAL 107884	HE649768	HE649671	HE649576	37°52'	13°22'		Tison et al., 2013

G. soleirolii F.W.Schultz ex Mutel	Spain, Morocco, Corsica, Sardinia**	Italy: Sardinia, M. Bruncu Spina	M	DID-1	-	HAL 104327	AM903060	AM903070	-	40°02'	09°17'		Peruzzi et al., 2008a
		Italy: Sardinia, M. Limbara	M	DID-1	-	CLU 9257	AM409352	AM903069	-	40°51'	09°10'		Peruzzi et al., 2008a
		Italy: Sardinia, Nuoro - Fonni: montée au Bruncu Spinu	M	DID-1	did-87	HAL 107955	HE649769	HE649672	HE649577	40°02'	09°17'		Tison et al., 2013
		Portugal: Beira Baixa, Casal-da- Serra	M	DID-1	-	HAL 105938	HE649770	FM200220	-	40°02'		07°32'	Tison et al., 2013
		Spain: Salamanca, en la subida a la Pena de Francia, Paso de los Lobos	ES	DID-1	-	Montserrat et al. s.n. (Kew 20651)	EU939297	EU912330	-	40°51'		06°17'	*Zarrei et al., 2009*
G. subtrigona J.-M.Tison	Spain, Morocco, Algeria**	Morocco: Meknes - Timhadite, Tizi-n-Tretten	M	DID-41	did-88	HAL 107414	HE649771	HE649673	HE649578	33°54'		05°33'	Tison et al., 2013
		Morocco: Ouarzazate - Aguelmouss: près du Tizi-n-Tichka	M	DID-1	did-88	HAL 107412	HE649772	HE649674	HE649579	30°55'		06°53'	Tison et al., 2013
		Spain: Alcolea, voie express NII à 1,5 km à l'est du Puerto de Alcolea	M	DID-1	did-15	HAL 107410	HE649782	HE649684	HE649589	38°59'		04°06'	Tison et al., 2013
		Spain: Cazorla, Puerto de las Palomas	M	DID-42	did-88	HAL 107415 DNA1	HE649773	HE649675	HE649580	37°54'		03°00'	Tison et al., 2013
		Spain: Cazorla, Puerto de las Palomas	M	DID-24	did-88	HAL 107415 DNA2	HE649774	HE649676	HE649581	37°54'		03°00'	Tison et al., 2013
		Spain: Malaga – Ronda, à l'ouest du Puerto del Viento	M	DID-43	did-15	HAL 107411 DNA1	HE649779	HE649681	HE649586	36°43'		05°09'	Tison et al., 2013
		Spain: Malaga – Ronda, à l'ouest du Puerto del Viento	M	DID-24	did-88	HAL 107411 DNA2	HE649780	HE649682	HE649587	36°43'		05°09'	Tison et al., 2013
		Spain: Priego-de-Cordoba, plateau de Navazuelo	M	DID-41	did-91	HAL 107413 DNA1	HE649777	HE649679	HE649584	37°26'		04°11'	Tison et al., 2013
		Spain: Priego-de-Cordoba, plateau de Navazuelo	M	DID-1	did-90	HAL 107413 DNA2	HE649778	HE649680	HE649585	37°26'		04°11'	Tison et al., 2013
		Spain: Robledo, col de la N322 à la sortie W de la ville	M	DID-41	did-89	HAL 107409 DNA1	HE649775	HE649677	HE649582	38°45'		02°27'	Tison et al., 2013
		Spain: Robledo, col de la N322 à la sortie W de la ville	M	DID-1	did-90	HAL 107409 DNA2	HE649776	HE649678	HE649583	38°45'		02°27'	Tison et al., 2013
G. tenera Pascher	W. & C. Asia**	Kazakhstan: Ili-Alatau foothills, Almaty	A-SW	DID-44	did-92	HAL 118080	LT985427	LT985807	LN874798	43°15'	76°56'41''		Peterson et al., 2016; this study
		Kyrgyzstan: Osch	A-SW	DID-44	did-92	HAL 101843	AM238527	AM238535	AM422460	40°30'	72°48'		Peterson et al., 2008
		Iran: Khorassan, Baijgiran towards Quchan	A-SW	DID-45	did-92	Zarrei & Golzarian 35256 (TUH) (Kew 23152)	EU939272	EU912303	EU912074	37°35'	58°26'		*Zarrei et al., 2009*
G. villosa (M.Bieb.) Sweet	Europe, W. Asia, Algeria; Probably archaeophyte at the N. & W.parts of the area**	Austria: Niederoesterreich – Wien: Türkenschanzpark	ES	DID-46	did-93	HAL 107418	FN868220	FN868246	FN868188	48°12'	16°22'		Peruzzi et al., 2011
		Bulgaria: Village Sushevo	ES	DID-46	did-95	PI *s.n.*	FN868214	FN868239	FN868189	43°48'	26°37'		Peruzzi et al., 2011
		Bulgaria: Chepan Hills	ES	DID-46	did-96	GAT HKEP1451a	LT985434	LT985784	LT985646	42,937534	22,951524		this study
		France: Corse, Sorio: au niveau du col entre les Monte Grimaesta et Astu	M	DID-46	did-94	HAL 107419	FN868218	FN868244	FN868190	42°34'	09°16'		Peruzzi *et al.*, 2011
		Georgia: near Tiblissi, Turtle Lake	A-SW	DID-46	-	61/08DNALevichev (LE)	LT985429	LT985779	-	41°42'	44°45'		this study
		Germany: Saxony-Anhalt	ES	DID-46	did-97	-	AJ416373	AJ419163	AJ427545	51°57'	11°41'		Peteson et al., 2008
		Germany: Thuringia, Jena	ES	DID-46	-	HAL 117637	LT985428	LT985778	-	50°53'60''	11°32'5''		this study
		Germany: Karzberg	ES	-	did-98	WÖRZ & ENGELHARDT 27.03.21.04. (STU)	-	-	FR874914	48°52'	10°24'		*Wörz et al., 2012*
		Greece: Epiros – Monts Timfi, parc national du Vikos-Aoos	M	DID-46	did-99	HAL 107420	FN868217	FN868243	FN868192	39°30'	20°54'		Peruzzi et al., 2011
		France: Alpes-Maritimes, Saint-Dalmas-le-Selvage	ES	DID-46	-	HAL 107422	FN868213	FN868242	-	44°17'	6°51'		Peruzzi et al., 2011
		France: Optevoz: cimetière	ES	DID-46	did-100	HAL 107421	FN868219	FN868245	FN868191	45°45'	5°19'		Peruzzi et al., 2011
		Iran	A-SW	DID-46	-	GAT13218	LT985430	LT985780	-				this study
		Iran: Tehran	A-SW	DID-49	did-109	Zarrei & Kamrani 35273 (TUH) (Kew 23178)	EU939276	EU912307	EU912084	36°7'	50°31'		*Zarrei et al., 2009*
		Iran: Khorassan, Bojnourd towards Gorgan	A-SW	DID-49	did-111	Zarrei & Golzarian 35253 (TUH) (Kew 23295)	EU939279	EU912310	EU912087	37°32'	56°22'		*Zarrei et al., 2009*
		Iran: Mazandaran, Firouzkuh towards Tehran, 3 km to Chel-Chesme restaurant	A-SW	DID-50	did-110	Zarrei & Golzarian 35247 (TUH) (Kew 23179)	EU939277	EU912308	EU912085	36°13'	52°31'		*Zarrei et al., 2009*
		Italy: Abruzzo, Barisciano	M	DID-46	did-101	CLU 14928	AM282991	AM265605	AM287283	42°19'	13°35'		Peruzzi et al., 2008a
		Italy: Calabria, Verbicaro-Orsomarso mountains	M	DID-46	did-102	CLU 15219	AM282992	AM265604	AM409345	39°50'	16°03'		Peruzzi et al., 2008a
		Italy: Piemonte, Valle Stura di Demonte	M	DID-46	did-103	PI *s.n.*	FN868215	FN868240	FN868193	44°22'	6°58'		Peruzzi et al., 2011
		Moldova: cultivated	ES	DID-46	did-108	HAL 101807	AJ973170	AM238538	AM180453				Peterson et al., 2008
		Russia: Krasnodarskii Krai	ES	DID-46	-	62/08DNA Levichev (LE)	LT985431	LT985781	-	44°28'	41°42'		this study
		Spain: Valdelinaves,Tervel	M	DID-46	-	HAL116084	LT985433	LT985783	-	40°23'		0°36'	this study
		Turkey: Antalya, Alanya-Hadim	M	DID-51	did-112	GAT HKEP1349b	LT985432	LT985782	LT985647	36°34'13''	32°21'42''		this study
		Turkey: Central Taurus (Isauria), Ibradi district, Gembos pasture	M	DID-46	did-105	PI *s.n.*	HE649784	HE649686	HE649591	37°00'	30°26'		Tison et al., 2013
		Turkey: Western Taurus (Lycia),Thermessos National Park	M	DID-46	did-106	PI s.n.:	HE649785	HE649687	HE649592	36°59'	30°28'		Tison et al., 2013
		Turkey: Western Taurus (Lycia), Elmali district, Cedrus Research Forest	M	DID-47	did-104	PI *s.n.*	HE649783	HE649685	HE649590	36°44'	29°55'		Tison et al., 2013
G. cf. *villosa*		Israel: Mt. Hermon	M	DID-48	did-107	HAL 116003	LT985435	LT985785	LT985648	33°13'	35°41`		this study
Gagea													
G. aipetriensis Levichev	Europe*	Ukraine: Crimea, Ai Petri	ES	GAG-1	gag-1	HAL 101801	AM049259	AJ970178	AM087955	44°45'	34°05'		Peterson et al., 2008
G. almaatensis Levichev, A.Peterson & J.Peterson	Kazhaksthan: Ili-Alatau range	Kazakhstan: Ili-Alatau range, Lake Bol. Almatinskoje	A-SW	GAG-2	gag-2	HAL 119703	LN874710	LN874781	LN874873	43°03'09''	76°59'23''		Peterson et al., 2016
		Kazakhstan: Ili-Alatau range, Lake Bol. Almatinskoje	A-SW	GAG-2	gag-2	HAL 119702	LN874711	LN874782	LN874874	43°03'09''	76°59'23''		Peterson et al., 2016
		Kazakhstan: Ili-Alatau range, Shymbulak ski ressort	A-SW	GAG-2	gag-2	HAL 119714	LN874712	LN874783	LN874875	43°08'24''	77°04'25''		Peterson et al., 2016
		Kazakhstan: Ili-Alatau range, Shymbulak ski ressort	A-SW	GAG-2	gag-2	HAL 119695	LN874713	LN874784	LN874876	43°06'44''	77°04'25''		Peterson et al., 2016
G. almaatensis × G. *kuraminica*		Kazakhstan: Ili-Alatau range, Shymbulak ski ressort	A-SW	GAG-2	gag-3	HAL 119688	LN874714	LN874785	LN874877	43°08'24''	77°04'25''		Peterson et al., 2016
G. ancestralis Levichev	Siberia*	Russia: Republic Altai, Gora Kurkure-Bazhi	ES	GAG-2	gag-2	144/10DNA Levichev (LE)	FR690844	FR690249	FR691052	51°05'	88°04'		Peterson et al., 2011
G. angelae Levichev & Schnittler	China: Xinjiang, Eastern Tian-Shan*	China: Xinjiang, Tian-Shan, Bogda-Shan, Tianchi Lake	A-E	GAG-3	gag-2	HAL 108470	FR691030	FR690261	FR690106	43°53'38″	88°06'59″		Peterson et al., 2011
		China: Xinjiang, Tian-Shan, Bogda-Shan, Tianchi Lake	A-E	GAG-3	gag-2	HAL 108477	FR691031	FR690262	FR690107	43°50'17″	88°10'15″		Peterson et al., 2011
		China: Xinjiang, Tian-Shan, Bogda-Shan, Tianchi Lake	A-E	GAG-3	gag-2	HAL 108475	FR691033	FR690264	FR690109	43°53'31″	86°06'02″		Peterson et al., 2011
		China: Xinjiang, Tian-Shan, Bogda-Shan, Tianchi Lake	A-E	GAG-3	gag-2	HAL 108476	FR691032	FR690263	FR690108	43°53'31″	86°06'02″		Peterson et al., 2011
		China: Xinjiang, Tian-Shan, Bogda-Shan, Tianchi Lake	A-E	GAG-3	gag-2	HAL 108474	FR691034	FR690265	FR690110	43°53'31	86°06'02″		Peterson et al., 2011
		China: Xinjiang, Tian-Shan, Bogda-Shan, Tianchi Lake	A-E	GAG-3	gag-2	HAL 108473	FR691035	FR690266	FR690111	43°53'31″	86°06'02″		Peterson et al., 2011
		China: Xinjiang, Tian-Shan, Bogda-Shan, Tianchi Lake	A-E	GAG-3	gag-2	HAL 108472	FR691036	FR690267	FR690112	43°53'31″	86°06'02″		Peterson et al., 2011
		China: Xinjiang, Tian-Shan, Bogda-Shan, Tianchi Lake	A-E	GAG-3	gag-2	HAL 108471	FR691037	FR690268	FR690113	43°53'31″	86°06'02″		Peterson et al., 2011
G. artemczukii Krasnova	Europe*	Ukraine: Crimea: Karadagskiy Zapovednik reserve	ES	GAG-4	gag-4	HAL 101481	AM409339	AM180470	AM409331	44°56' 3.037	35°13' 24.01''		Peterson et al., 2008
Gagea capusii A.Terracc.	Pamir-Alai, South-west Asia, Western Tien Shan*	Uzbekistan: Gissarski	A-SW	GAG-5	gag-5	HAL 101839	AM085143	AJ969123	AM422455	39°00'	68°05'		Peterson et al., 2008
G. erubescens (Besser) Besser	Europe*	Ukraine: near Charkow	ES	GAG-6	gag-1	HAL 103861	AM238516	AM180469	AM493953	50°0'	36°15'		Peterson et al., 2008

G. fedtschenkoana Pascher	Siberia, South-west Asia*	Kazakhstan: Astana	A-SW	GAG-7	gag-6	HAL 117627	LN874696	LN874767	LN874849	51°10'52''	71°25'23''	Peterson et al., 2016
		Russia: Altai; Barnaul	ES	GAG-8	-	145/10DNA Levichev (LE)	LT985436	LT985786	-	53°20'	83°46'	this study
G. cf. *fedtschenkoana Pascher*		Kazakhstan: Tenis-Korgalshinskii nature reserve	A-SW	GAG-9	gag-1	HAL 117626	LN874697	LN874768	LN874850	50°28'38''	69°32'44''	Peterson et al., 2016
		Kazakhstan: Tenis-Korgalshinskii nature reserve	A-SW	GAG-9	gag-1	HAL 117628	LN874698	LN874769	LN874851	50°29'45''	69°32'48''	Peterson et al., 2016
G. helenae Grossh.	Europe, South-west Asia*	Russia: Dagestan, Machatschkala	ES	GAG-6	gag-7	HAL 101798	AM161461	AJ969120	AM265531	42°59'	47°29'	Peterson et al., 2008
G. huochengensis Levichev	China, Xinjiang, eastern Tian-Shan	China: Xinjiang, Tian-Shan, Bogda-Shan, Tianchi Lake	A-E	GAG-2	gag-2	HAL 108466	FR691038	FR690269	FR690114	43°53′38″	88°06′59	Peterson et al., 2011
		China: Xinjiang, Tian-Shan, Bogda-Shan, Tianchi Lake	A-E	GAG-2	gag-2	HAL 108465	FR691039	FR690270	FR690115	43°53′38″	88°06′59″	Peterson et al., 2011
		China: Xinjiang, Tian-Shan, Bogda-Shan, Tianchi Lake	A-E	GAG-3	gag-2	HAL 108467	FR691040	FR690271	FR690116	43°50′17″	88°10′15″	Peterson et al., 2011
		China: Xinjiang, Tian-Shan, Bogda-Shan, Tianchi Lake	A-E	GAG-3	gag-2	HAL 108469	FR691043	FR690273	FR690119	43°50′17″	88°10′15″	Peterson et al., 2011
		Kazakhstan: Markakolskiy Nature Reserve	A-SW	GAG-3	gag-2	HAL 108468	FR691041	FR690272	FR690117	48°41'	65°18'	Peterson et al., 2011
G. kuraminica *Levichev*	South-west Asian,Western Tien Shan*	Uzbekistan: Tian-Shan, Kurama range (2500 m)	A-SW	GAG-10	gag-8	151/14DNALevichev (LE)	LN874709	LN874780	LN874862	40°45'	70°10'	Peterson et al., 2016
		Kazakhstan: Ili-Alatau rangs, Medeu statium	A-SW	GAG-8	gag-3	HAL 119683	LN874699	LN874770	LN874852	43°11'15''	77°00'07''	Peterson et al., 2016
		Kazakhstan: Ili-Alatau range, Medeu statium	A-SW	GAG-8	gag-3	HAL 119685	LN874700	LN874771	LN874853	43°10'02''	77°04'36''	Peterson et al., 2016
		Kazakhstan, Ili-Alatau range, Medeu statium	A-SW	GAG-8	gag-9	HAL 119704	LN874701	LN874772	LN874854	43°10'09''	77°02'25''	Peterson et al., 2016
		Kazakhstan, Ili-Alatau range, Shymbulak ski ressort	A-SW	GAG-8	gag-3	HAL 119687	LN874702	LN874773	LN874855	43°08'24''	77°04'25''	Peterson et al., 2016
		Kazakhstan, Ili-Alatau range, Shymbulak ski ressort	A-SW	GAG-8	gag-3	HAL 119689	LN874703	LN874774	LN874856	43°08'24''	77°04'25''	Peterson et al., 2016
		Kazakhstan, Ili-Alatau range, Shymbulak ski ressort	A-SW	GAG-8	gag-3	HAL 119697	LN874704	LN874775	LN874857	43°06'59''	77°04'26''	Peterson et al., 2016
		Kazakhstan, Ili-Alatau range, Shymbulak ski ressort	A-SW	GAG-8	gag-3	HAL 119713	LN874705	LN874776	LN874858	43°08'24''	77°04'25''	Peterson et al., 2016
		Kazakhstan, Ili-Alatau range, Shymbulak ski ressort	A-SW	GAG-8	gag-3	HAL 119700	LN874706	LN874777	LN874859	43°06'59''	77°04'26''	Peterson et al., 2016
		Kazakhstan, Ili-Alatau range, Shymbulak ski ressort	A-SW	GAG-8	gag-3	HAL 119693	LN874707	LN874778	LN874860	43°06'44''	77°04'26''	Peterson et al., 2016
		Kazakhstan, Ili-Alatau range, Shymbulak ski ressort	A-SW	GAG-8	gag-10	HAL 119692	LN874708	LN874779	LN874861	43°06'44''	77°04'26''	Peterson et al., 2016
G. kuraminica × G. *almaaatensis*		Kazakhstan, Ili-Alatau range	A-SW	GAG-8	gag-2	HAL 119701	LN874715	LN874786	LN874878	43°03'22	76°58'05	Peterson et al., 2016
G. lutea (L.) Ker Gawl.	Europe, Mediterranean, South-west Asian*	Austria: Wien, Bisamberg	ES	GAG-11	-	HAL 21276	LT985437	LT985787	-	48°33'	16°36'	this study
		Germany: Lübeck	ES	-	gag-12	WÖRZ 10.04.16.01. (STU)	-	-	FR874905	53°51'	10°41'	*Wörz et al., 2012*
		Germany: Saxony-Anhalt: Rothenschirmbach	ES	GAG-11	gag-11	no	AJ416368	AJ419165	AJ427544	51°27'	11°32'	Peterson et al., 2004
		Germany: Thuringia, Erfurt, Mühlburg (var lauca)	ES	GAG-11	gag-12	HAL 117484	LT985440	LT985790	LT985625	50°51'58''	10°50'20''	this study
		Germany: Thuringia, Jena	ES	GAG-11	-	HAL 117630	LT985439	LT985789	-	50°53'60''	11°32'5''	this study
		Germany: Tübingen	ES	-	gag-13		-	-	FR874916	48°31'	9°3'	*Wörz et al., 2012*
		Germany: Western-Pomerania, Gingst	ES	GAG-11	-	HAL 112577	LT985438	LT985788	-	54°27'	13°15'	this study
		Iran: Kuh-e Abr c. 5 km NW vill. Abr	A-SW	GAG-6	-	HAL116009	LT985444	LT985795	-	32°50'	48°32'	this study
		Iran: Mazandaran, Karaj towards Chalus	A-SW	GAG-6	gag-14	Zarrei 35285 (TUH) (Kew 23148)	EU939250	EU912281	EU912052	36°12'69	51°18'97	*Zarrei et al., 2009*
		Italy: Calabria (Verbicaro-Orsomarso mountains)	M	GAG-11	gag-12	CLU 12696	AM409354	AM265606	AM287282	39°45'	15°55'	Peruzzi et al., 2008a
		Italy: Madonna dell'Acero (Appennino Bolognese)	M	GAG-11	-	PI *s.n.*	LT985441	LT985792	-	44,14841	10,82223	this study
		Italy: Monte Cetona	M	GAG-11	-	PI *s.n.*	LT985442	LT985793	-	42° 55' 57.576''	11° 52' 16.579''	this study
		Italy: Valle di Bellino (Cuneo)	M	GAG-11	-	PI *s.n.*	LT985443	LT985794	-	44°35′	7°02	this study
		Russia: Caucasus, Mineralnye Vody	ES	GAG-11	gag-12	HAL 101800	AM161456	AM110255	AM265530	44°12'42.03''	43°7'56.582''	Peterson et al., 2008
		Sweden: Upsala	ES	GAG-11	-	HAL 17770	LT985445	LT985791	-	59° 51'	17° 38'	this study
G. nakaiana Kitag	East -Asian*	Russia: Khabarovsk Territory	A-E	GAG-14	gag-17	HAL 101799	AM161457	AM110256	AM180454	48°48	135°1'	Peterson et al., 2008
G. paczoskii (Zapal.) Grossh.	Europe*	Ukraine: Luga	ES	GAG-15	-	HAL 103858	AM493969	AM265521	-	58°44'	29°51'	Peterson et al., 2008
G. podolica Schult. & Schult. f.	Europe*	Ukraine: Slavjanogorsk	ES	GAG-6	gag-19	HAL 101832	AM238525	AM084903	AM409334	49°02'	37°35'	Peterson et al., 2008
G. pomeranica Ruthe	Germany, France	Germany: Mecklenburg-Western Pomerania, Stolpe (Anklam)	ES	GAG-17	gag-21	HAL 095846	AJ429194	LN874787	AJ429193	53°52'	13°33'	Peterson et al., 2004
		Germany, Eichert NTrochtelfingen/Ries	ES	-	gag-22	WÖRZ & ENGELHARDT 27.03.21.05 (STU)	-	-	FR874912	48°18'	09°14'	*Wörz et al., 2012*
		Germany: Saxony-Anhalt, Rothenschirmbach	ES	GAG-17	gag-20	HAL 095842	AJ416375	AJ419167	AJ427543	51° 27' 11.941''	11° 32' 46.644''	Peterson et al., 2004
		Germany: Thuringia, Jena	ES	GAG-11	-	HAL 117487	LT985446	LT985796	-	50°54'	11°32'	this study
		France: Montauban-sur-l'Ouvèze: montagne de Chamouse	M	GAG-11	-	HAL 103168	LT985447	LT985797	-	44°16'	5°33'	this study
G. pomeranica (=Gagea marchica Henker, Kiesew, U.Raabe & Rätzel)	Germany	Germany: Alt-Zeschdorf, Ortsteil Hohenjesar. Grasflächen auf dem Kirchhof	ES	GAG-12	gag-15	HAL 118006	LN874683	LN874754	LN874808	52°25'	14°25'	Peterson et al., 2016
G. pomeranica (=G. megapolitana Henker)	Germany	Germany: Mecklenburg-Western Pomerania, Wismar (Lindengarten)	ES	GAG-13	gag-16	HAL 101644	AM161455	AM084902	AM931553	53°53'	11°28'	Peterson et al., 2008
G. pratensis(Pers.) Dumort.	Europe, Mediterranean*	Czech Republik: Grahan, Bohnice	ES	GAG-17	-	HAL 079600	LT985451	LT985845	-	50°8'	14°25'	this study
		Germany: Brandenburg, Milower Berg	ES	GAG-17	gag-24	-	AJ437195	consensus (AJ416372)	AJ437202	52°31'	12°18'	Peterson et al., 2004
		Germany: Nördlingen	ES	-	gag-25	WÖRZ & ENGELHARDT 27.03.21.07. (STU)	-	-	FR874915	48°51'	10°29'	*Wörz et al., 2012*
		Germany: Saxony-Anhalt, Hegegrund (Volkstedt)	ES	GAG-17	gag-23	HAL 095847	AJ416372	AJ419162	AJ427542	51°33'	11°33'	Peterson et al., 2004
		Germany: Saxony-Anhalt, Laweketal (Räther)	ES	GAG-17	gag-23	HAL 095845	AJ416372	AJ437196	AJ427542	51°32'	11°42'	Peterson et al., 2004
		Germany: Saxony-Anhalt, Rothenschirmbach	ES	GAG-17	-	HAL 095848	LT985450	LT985844	-	51°27'	11°32'	this study
		Germany: Thuringia, Jena	ES	GAG-17	-	HAL 117631	LT985449	LT985843	-	50°53'60''	11°32'5''	this study
		Germany: Western Pomerania, Gingst	ES	GAG-18	-	HAL 112576	LT985448	LT985842	-	54°27'	13°15'	this study
		Italy: Calabria (Verbicaro-Orsomarso mountains)	M	GAG-17	gag-26	CLU 12703	AM903059	AM265607	AM903047	39,8418	16,05544	Peruzzi et al., 2008a
		Italy: Tuscany, Monte Cetona	M	GAG-23	-	PI *s.n.*	LT985452	LT985846	-	42°56'	12°22'	this study
G. pusilla (F.W.Schmidt) Sweet	Europe*	cultivated		GAG-19	gag-27	HAL 101831	AM161458	AM180464	AM422453	-	-	Peterson et al., 2008
		Serbia: East of Negotin, Donau valley	ES	GAG-19	gag-27	HAL 118008	LT985603	LT985847	LT985626	44,22909	22,59476	this study
		Serbia: South of Kladovo, Donau valley	ES	GAG-19	gag-27	HAL 118009	LT985604	LT985848	LT985627	44,55231	22,64811	this study
G. cf. *pusilla*		Italy: Friuli-Venezia Giulia (Basovizza)	M	GAG-16	gag-18	HAL 104323/HAL 104339	AM903061	AM903072	AM903051	45°38'	13°51'	Peruzzi et al., 2008a
		Italy: Friuli-Venezia Giulia (Gabrovizza)	M	GAG-16	gag-18	HAL 104338	AM903057	AM903071	AM903052	45°43'	13°43'	Peruzzi et al., 2008a
G. rubicunda Meinsh. emend. Levichev	Europe*	Estonia	ES	GAG-20	gag-28	HAL 103855	AM409338	AM238541	AM493954	58°08'	27°31'	Peterson et al., 2008
G. shmakoviana Levichev	Sibiria*	Russia: Altai Krai	ES	GAG-21	gag-29	HAL 101830	AM287265	AM265520	AM422454	52°46'	82°37'	Peterson et al., 2008
*G. terracciano*a*na* Pascher	East Asia*	Mongolia: Bogd-Ul Mountains	A-E	GAG-22	gag-30	HAL 070426	AJ973169	AJ890367	AM287279	47,8	106,99	Peterson et al., 2008
		Russia: Primorsky Krai, Dalnegorsk	A-E	GAG-22	gag-31	HAL 103853	AM287268	AM287280	AM493955	44°33'	135° 37'	Peterson et al., 2008
G. tisoniana Peruzzi, Bartolucci, Frignani & Minut.	Italy	Italy: Tuscany, Cornate di Gerfalco	M	GAG-16	gag-32	CLU 14920	AM409349	AM409358	AM422466	43°6'	10°54'	Peruzzi et al., 2008a
		Italy: Umbria, Monti Martani	M	GAG-16	-	PI *s.n.*	LT985605	LT985849	-	43.02	12.64	this study
		Italy: Umbria, Monte di Patrico	M	GAG-16	-	PI *s.n.*	LT985607	LT985851	-	42.70	12.76	this study
		Italy: Tuscany, Sassi Bianchi	M	GAG-16	-	PI *s.n.*	LT985606	LT985850	-	43.48	10.64	this study
G. transversalis Steven	Europe*	Ukraine: Crimea, Ai Petri	ES	GAG-1	gag-1	HAL 101783	AJ973167	AJ890370	AM162671	44° 45'	34° 05'	Peterson et al., 2008

G. turkestanica Pascher		Kyrgyzstan: Kyrgyz Range	A-SW	GAG-24	-	HAL 103850	AM287267	AM238534	-				Peterson et al., 2008
G. cf. *turkestanica* Pascher	South west Asia; Western Tian Shan*	Kazakhstan: Ili-Alatau foothills, Almaty	A-SW	GAG-25	gag-35	HAL 118082	LN874686	LN874757	LN874865	43°15'00"	76°56'41"		Peterson et al., 2016
		Kazakhstan: Ili-Alatau foothills, Almaty	A-SW	GAG-8	gag-34	130/09DNALevichev (LE)	LN874687	LN874758	LN874866	43°13'	76°51'		Peterson et al., 2016
		Kazakhstan: Ili-Alatau foothills, Almaty	A-SW	GAG-27	-	119/09DNALevichev (LE)	LN874689	LN874760	-	43°13'	76°51'		Peterson et al., 2016
		Kazakhstan: Ili-Alatau foothills, Almaty	A-SW	GAG-8	gag-34	HAL 119678	LN874690	LN874761	LN874867	43°15'00"	76°56'41"		Peterson et al., 2016
		Kazakhstan: Ili-Alatau foothills, Almaty	A-SW	GAG-8	gag-34	HAL 119679	LN874691	LN874762	LN874868	43°15'00"	76°56'41"		Peterson et al., 2016
		Kazakhstan: Ili-Alatau foothills, Almaty	A-SW	GAG-8	gag-34	HAL 119680	LN874692	LN874763	LN874869	43°13'34"	76°54'49"		Peterson et al., 2016
		Kazakhstan: Ili-Alatau foothills, Almaty	A-SW	GAG-8	gag-34	HAL 119708	LN874693	LN874764	LN874870	43°13'49"	76°58'38"		Peterson et al., 2016
		Kazakhstan: Ili-Alatau foothills, Almaty	A-SW	-	gag-36	HAL 119705	-	-	LN874871	43°13'36"	76°58'26"		Peterson et al., 2016
		Kazakhstan: Ili-Alatau range, Uschkonyr	A-SW	-	gag-37	HAL 119711	-	-	LN874872	43°13'15"	76°33'10"		Peterson et al., 2016
		Kazakhstan: Ili-Alatau range, Turar	A-SW	GAG-8	gag-33	HAL 119707	LN874684	LN874755	LN874863	43°14'58"	76°32'13"		Peterson et al., 2016
		Kazakhstan: Ili-Alatau range, Turar	A-SW	GAG-8	gag-34	HAL 119710	LN874685	LN874756	LN874864	43°14'58"	76°32'13"		Peterson et al., 2016
		Kazakhstan: Jezkazgan	A-SW	GAG-26	-	131/09DNALevichev (LE)	LN874688	LN874759	-	47°47'	67°42'		Peterson et al., 2016
G. xiphoidea Levichev	Sibiria*	Russia: Republic Altai, district Kosch-Agaczensis	ES	GAG-2	gag-2	71/09DNALevichev (LE)	FR691044	FR690138	FR690097	49°59'	88°40'		Peterson et al., 2011
		Russia, Republic Altai, district Kosch-Agaczensis	ES	GAG-2	gag-2	102/09DNALevichev (LE)	FR691045	FR690139	FR690098	49°59'	88°40'		Peterson et al., 2011
G. cf. *xiphoidea*	China: Xinjiang, Eastern Tian-Shan	China, Xinjiang, Tian-Shan, Bogda-Shan, Tianchi Lake,	A-E	GAG-2	gag-2	HAL 108479	FR691046	FR690140	FR690099	43°53′31″	86°06′02″		Peterson et al., 2011
		China, Xinjiang, Tian-Shan, Bogda-Shan, Tianchi Lake	A-E	GAG-2	gag-2	HAL 108478	FR691047	FR690141	FR690100	43°53′31″	86°06′02″		Peterson et al., 2011
Incrustatae **Boiss.**													
G. circumplexa Vved.	Pamir-Alai, South-west Asian, Western Tien Shan*	Uzbekistan	A-SW	INC	inc	HAL 101795	AJ969122	AJ973172	AM265529	40°23`	71°47`		Peterson et al., 2008
Lloydia (Rchb.) Peruzzi, J.-M. Tison, A. Peterson & J. Peterson													
G. noltiei Peruzzi, J.-M. Tison, A. Peterson & J.Peterson	India (Sikkim), CE Nepal, Buthan	India, SikkimWest District	A-H	LOY-1	-	ESIK 385 772*1 (E)	AM493965	AM493961	-	27°33'	88°07'		Peterson et al., 2008
		Nepal: Kanchenjunga: Simbo Khola above Yalung	A-H	LOY-1	-	McBeath 2501 772*2 E	AM493962	AM493966	-	27°42'	88°8'		Peterson et al., 2008
G. serotina (L.) Ker Gawl.	Pakistan, Kashmir, India, Nepal, Bhutan, China, Kazakhstan, Mongolia, Russia, Korea, Japan, North America, Europe* Arctic (N America & N Asia)***	Bulgaria: Ovtscharez	ES	LOY-2	-	HAL 74806	AJ585048	AJ585049	-	42°8'60"	23°34'60		Peterson et al., 2008
		Bulgaria: M Rila, Juruski Cal	ES	LOY-2	-	GAT 23117	LT985594	LT985822	-	42°11'	23°35'		this study
		Great Britain: Wales, Snowdonia	ES	-	loy-1	Jones *s.n.* (K) (Kew 1004)	-	-	EU912092	52°55		3°53	*Zarrei et al., 2009*
		Italy: Alpi Bresciane, Passo di Valfredda	ES	LOY-2	loy-2	PI *s.n.*	LT985595	LT985823	LT985628	46°25'	11°49'		this study
		Kazakhstan: Leninogorsk	A-SW	LOY-2	loy-3	HAL 101789	AM238530	AJ890376	AM087956	50°19'	83°30'		Peterson et al., 2008
		Poland: Western Carphatian	ES	LOY-2	loy-2	HAL 143063	LT985596	LT985824	LT985629	49°13'44"	20°02'45"		this study
		Russia: Tuva	ES	LOY-3	-	HAL 103849	AM409337	AM493963	-	51°53	95°37`		Peterson et al., 2008
		Russia: Karachaevo-Cherkessian Rep., North Caucasus	ES	LOY-4	-	HAL 117623	LT985601	LT985829	-	43°26'5"	41°41'14"		this study
		Russia: Karachaevo-Cherkessian Rep., North Caucasus	ES	LOY-4	loy-4	HAL 117624	LT985602	LT985830	LN874806	43°26'0"	41°41'36"		Peterson et al., 2016; this study
		USA: Alaska, Alaska range	CB	LOY-2	-	HAL 117488	LT985597	LT985825	-	63°44'33"		148°57'11"	this study
		USA: Alaska, Alaska range	CB	LOY-2	loy-5	HAL 117490	LT985599	LT985827	LN874807	63°41'16"		149°41'16"	Peterson et al., 2016; this study
		USA: Alaska, Alaska range	CB	LOY-2	-	HAL 117491	LT985600	LT985828	-	63°34'30"		149°47'25"	this study
		USA: Alaska, Brook range	CB	LOY-2	-	HAL 117489	LT985598	LT985826	-	67°55'18"		149°42'35"	this study
Minimae (Pascher) Davlian.													
G. confusa A.Terracc.	South-west Asian*	Azerbaijan:: Nr. border of Iraq	A-SW	MIN-2	min-2	TUH-E BOT.EXP. 35712 (TUH) (Kew 23169)	EU939239	EU912268	EU912041	38°12`	47°52'20"		*Zarrei et al., 2009*
		Iran: Tehran, prov. Tochal	A-SW	MIN-1	min-1	HAL 101803	AJ973173	AJ890369	AM087949	35°58'	52°11'		Peterson et al., 2008
		Iran: Tehran, Tochal Mts	A-SW	MIN-3	min-3	Zarrei & Zarrei 35266 (TUH) (Kew 23137)	EU939238	EU912267	EU912040	35°53'	51°25'		*Zarrei et al., 2009*
G. davlianidzeae Levichev	Uzbekistan, Kyryzstan, Tajikistan, Afghanistan, Pakistan, N. India[#], China: Xinjiang, Eastern Tian-Shan	China: Xinjiang, Tian-Shan, Bogda-Shan,Tianchi Lake	A-E	MIN-4	min-4	HAL 107946	FR690832	FR690237	FR689756	43°50′17″	88°10′15"		Peterson et al., 2011
		China: Xinjiang, Tian-Shan, Bogda-Shan,Tianchi Lake	A-E	MIN-4	min-4	HAL 107943	FR690240	FR690240	FR689759	43°53′51"	88°07′51"		Peterson et al., 2011
		China: Xinjiang, Tian-Shan, Bogda-Shan,Tianchi Lake	A-E	MiN-4	min-4	HAL 107941	FR690836	FR690241	FR689760	43°54′44"	88°07′00"		Peterson et al., 2011
		China: Xinjiang, Tian-Shan, Bogda-Shan,Tianchi Lake	A-E	MIN-4	min-4	HAL 107939	FR690242	FR690242	FR689761	43°54′15"	88°06′51″		Peterson et al., 2011
		China: Xinjiang, Tian-Shan, Bogda-Shan,Tianchi Lake	A-E	MIN-4	min-5	HAL 107948	FR690831	FR690236	FR689755	43°53′52"	88°08′08"		Peterson et al., 2011
G. filiformis (Ledeb.) Kar. & Kir.	EastAsian, Pamir Alai, Sibirian, Soth-west Asian, Wstern Tien Shan*	Kazakhstan	A-SW	MIN-6	min-7	HAL 101804	AM161459	AM084904	AM180457	48°44'	83°28'		Peterson et al., 2008
		Kazakhstan, Ili-Alatau foothills,Almaty, around TV tower	A-SW	MIN-7	min-6	HAL 118070	LN874660	LN874731	LN874824	43°13'36"	76°58'26"		Peterson et al., 2016
		Kazakhstan, Ili-Alatau foothills, Almaty, around TV tower	A-SW	MIN-7	min-6	HAL 118070	LN874660	LN874731	LN874824	43°13'36"	76°58'26"		Peterson et al., 2016
		Kazakhstan, Ili-Alatau foothills, Almaty, around TV tower	A-SW	MIN-8	min-6	HAL 118073	LN874661	LN874732	LN874825	43°13'50"	76°58'29"		Peterson et al., 2016
		Kazakhstan: Ili-Alatau range, Almaty, Lake Bol. Almatinskoje	A-SW	MIN-7	min-6	HAL 118069	LN874659	LN874730	LN874823	43°03'22"	76°58'05"		Peterson et al., 2016
		Kazakhstan: Ili-Alatau range: Almaty, Medeu stadium	A-SW	MIN-8	min-6	HAL 118077	LN874665	LN874736	LN874829	43°09'06"	77°04'01"		Peterson et al., 2016
		Kazakhstan: Ili-Alatau range, Almaty, Medeu statium	A-SW	MIN-7	min-6	HAL 118050	LN874646	LN874717	LN874810	43°09'60"	77°04'28"		Peterson et al., 2016
		Kazakhstan: Ili-Alatau foothills, Almaty, Medeu stadium	A-SW	-	min-6	HAL 118075	-	-	LN874827	43°10'48"	77°00'43"		Peterson et al., 2016
		Kazakhstan: Ili-Alatau foothills, Almaty, Medeu stadium	A-SW	-	min-6	HAL 118076	-	-	LN874828	43°10'48"	77°00'43"		Peterson et al., 2016
		Kazakhstan: Ili-Alatau range, Almaty, Shymbulak ski ressort	A-SW	MIN-7	min-6	HAL 118065	LN874654	LN874725	LN874818	43°06'44"	77°04'06"		Peterson et al., 2016
		Kazakhstan, Ili-Alatau range, Almaty, Shymbulak ski ressor,	A-SW	MIN-8	min-6	HAL 118055	LN874649	LN874720	LN874813	43°08'30"	77°03'51"		Peterson et al., 2016
		Kazakhstan: Ili-Alatau range, Almaty, Shymbulak ski ressort	A-SW	MIN-4	min-10	HAL 118059	LN874666	LN874737	LN874830	43°08'24"	77°04'25"		Peterson et al., 2016
		Kazakhstan: Ili-Alatau range, Almaty, Shymbulak ski ressort	A-SW	MIN-4	-	HAL 118058	LN874740	LN874669	-	43°08'30"	77°03'51"		Peterson et al., 2016
		Kazakhstan: near Kegen Pass	A-SW	MIN-10	min-9	HAL 116095	LN874668	LN874739	LN874668	49°08'12'	83°20'13"		Peterson et al., 2016
		Kazakhstan: Sharyn Canyon	A-SW	MIN-9	min-8	HAL 115984	LN874667	LN874738	LN874832	43°12'07	79°12'18"		Peterson et al., 2016
		Western Tian-Shan	A-SW	MIN-5	min-6	72/09DNALevichev (LE)	FR690845	FR690250	FR689768	41°29'	70°03'		Peterson et al., 2011

G. granulosa Turcz.	East Asian, European, Sibirian, South-west Asia*	Kazakhstan: Ili-Alatau range	A-SW	MIN-4	min-13	HAL 118046	LN874677	LN874748	LN874842	43°08'30	77°03'51	Peterson et al., 2016
		Kazakhstan: Ili-Alatau foothills, Almaty	A-SW	MIN-4	min-11	HAL 118037	LN874670	LN874741	LN874835	43°13'34''	76°54'49	Peterson et al., 2016
		Kazakhstan, Ili-Alatau range, Almaty, Medeu statium	A-SW	MIN-4	min-12	HAL 118040	LN874674	LN874745	LN874839	43°09'37''	77°03'33''	Peterson et al., 2016
		Kazakhstan: Ili-Alatau range, Almaty, Shymbulak ski ressort	A-SW	MIN-11	min-15	HAL 118045	LN874678	LN874749	LN874843	43°08'30''	77°03'51	Peterson et al., 2016
		Kazakhstan: Tian-Shan, Ketmen	A-SW	MIN-4	min-14	11b/04DNALevichev (LE)	AM238517	AM28727	AM287278	43°27'13''	80°20'41''	Peterson et al., 2008
		Russia, Sibiria	ES	MIN-13	-	141/09DNALevichev (LE)	FR690848	FR690253	-	56°30'	84°59'	Peterson et al., 2016
		Ukrain:, Poltava	ES	MIN-12	-	100/09DNALevichev (LE)	FR690847	FR690252	-	49°34'	34°49'	Peterson et al., 2016
G. minima (L.) Ker Gawl.	European, Mediterranean, South-west Asia*	Czech Republic: Hrázský Mlýn, Zárubova stráň	ES	MIN-14	-	HAL 103610	LT985608	LT985831	-	49°58´19''	15°09´58''	this study
		France: Montagne de Lachens (Alpes Maritimes)	ES	MIN-14	-	PI *s.n.*	LT985609	LT985832	-	43°56'	07°10'	this study
		Germany: Bad Doberan	ES	-	min-17	WÖRZ 10.04.20.01. (STU)			FR874901	54°6'	11°54'	*Wörz et al., 2012*
		Germany: Saxony-Anhalt, Einetal (Welbsleben)	ES	MIN-14	min-16	-	AJ416374	AJ416374	AJ427546	51°42'25''	11°25'19''	Peterson et al., 2004
		Germany: Saxony-Anhalt, Kühlbachtal (Zörnitz)	ES	MIN-14	min-16	-	AJ416374	AJ419164	AJ427546	51°33'57''	11°45'54''	Peterson et al., 2004
		Italy: Calabria, Pollino Massif	M	MIN-14	min-18	CLU 12698	AM282996	AM283109	AM287273	39°53'	16°13'	Peruzzi et al., 2008a
		Italy: Lazio, Pian di Mezzo (Pianisco)	M	MIN-14	-	PI *s.n.*	LT985611	LT985834	-	41°39'	13°35'	this study
		Italy: Piemont, Valle Varaita, Sampeyre, St. Anna	ES	MIN-14	-	HAL 116000	LT985610	LT985833	-	44°34'	7°10'	this study
		Italy: Umbria Monti Sibillini	M	MIN-14	min-19	PI *s.n.*	LT985612	LT985835	LT985630	42°49'	13°16'	this study
		Russia: Republic Tatarstan	ES	MIN-14	min-21	HAL 101806	AM238519	AM180471	AM180459	55°36'	51°50'	Peterson et al., 2008
		Sweden: Smaland; Madesjö	ES	MIN-14	-	HAL 21281	LT985613	LT985836	-	56°44' 31.117''	15°53' 11.828''	this study
		Ukraine, Carpathians	ES	MIN-14	min-20	HAL 101808	AM238524	AM238539	AM087948	48°58'	24°02'	Peterson et al., 2008
G. nigra L.Z Shue	Eastern Tian Shan	China: Xinjiang, Tian-Shan, Bogda-Shan, Tianchi Lake	A-E	MIN-15	min-22	HAL 108459	FR691012	FR690125	FR690083	43°53'27''	88°06'46''	Peterson et al., 2011
		China: Xinjiang, Tian-Shan, Bogda-Shan, Tianchi Lake	A-E	MIN-15	min-22	HAL 108460	FR691015	FR690128	FR690088	43°53'31''	88°06'02''	Peterson et al., 2011
		China: Xinjiang, Tian-Shan, Bogda-Shan, Tianchi Lake	A-E	MIN-15	min-22	HAL 108456	FR691016	FR690129	FR690089	43°52'35''	88°07'41''	Peterson et al., 2011
		China: Xinjiang, Tian-Shan, Bogda-Shan, Tianchi Lake	A-E	MIN-15	min-22	HAL 108183	FR691018	FR690131	FR690091	43°50'17''	88°10'15''	Peterson et al., 2011
		China: Xinjiang, Tian-Shan, Bogda-Shan, Tianchi Lake	A-E	MIN-15	min-22	HAL 108457	FR691019	FR690132	FR690092	43°53'51''	88°08'23''	Peterson et al., 2011
		China: Xinjiang, Tian-Shan, Bogda-Shan, Tianchi Lake	A-E	MIN-15	min-22	HAL 108184	FR691021	FR690134	FR690094	43°53'38''	88°06'59''	Peterson et al., 2011
		China: Xinjiang, Tian-Shan, Bogda-Shan, Tianchi Lake	A-E	MIN-15	min-22	HAL 108452	FR691022	FR690135	FR690095	43°47'47''	87°34'38''	Peterson et al., 2011
		China: Xinjiang, Urumqi	A-E	MIN-15	min-22	HAL 108464	FR691010	FR690123	FR690081	43°43'53	87°35'46	Peterson et al., 2011
		China: Xinjiang, Urumqi	A-E	MIN-15	min-22	HAL 108462	FR691011	FR690124	FR690082	43°38'50''	87°39'04''	Peterson et al., 2011
		China: Xinjiang, Urumqi	A-E	MIN-15	-	134/10DNALevichev (LE)	FR691024	FR690137	-	43°47'	87°37'	Peterson et al., 2011
Persicae **(Levichev) Peruzzi**												
G. gageoides (Zucc.) Vved.	Pamir-Alai, South-west Asian, Western Tien Shan* Lebanon, Israel#	Afghanistan: Zentral-Nuristan, Mum-Pass	A-SW	PER-1	-	HAL 111510	LT985614	LT985860	-	35°32'	71°31'	this study
		Azerbaijan: nr border of Iraq, Dizaj	A-SW	PER-2	per-1	TUH-E BOT.EXP. 35714 (TUH) (Kew 23145)	EU939246	EU912276	EU912048	37°09'	44°52'09	*Zarrei et al., 2009*
		Iran: Tehran	A-SW	PER-3	per-2	Zarrei & Kamrani 35274 (TUH) (Kew 23172)	EU939247	EU912277	EU912049	36°7'	50°31'	*Zarrei et al., 2009*
		Kazakhstan: Leviy Bereg	A-SW	PER-4	per-3	HAL 101792	AM161462	AM084905	AM162673	50°36	83°5	Peterson et al., 2008
Platyspermum **Boiss.**												
G. alexeenkoana Miscz	Europe, South-west Asia*	Russia: Caucasus, Mineralnye Vody	ES	PLA-1	pla-1	HAL 101794	AM161460	AM110257	AM180458	44°22'	43°13'	Peterson et al., 2008
		Iran: Ardabil	A-SW	PLA-1	pla-2	TUH-E BOT.EXP. 35305(TUH) (Kew 23142)	EU939224	EU912250	EU912024	38°15'	48°17'	*Zarrei et al., 2009*
		Iran: Ardabil, Sarein, Sabalan Mts.	A-SW	-	pla-3	TUH-E BOT.EXP. 35306 (TUH) (Kew 23297)	-	-	EU912030	38°12'831	47°52'203	*Zarrei et al., 2009*
G. bergii Litv.	South-west Asia,Western Tien Shan*	Iran: Khorassan	A-SW	-	pla-4	Zarrei & Golzarian 35222 (TUH) (Kew 23141)	-	-	EU912026	32°31'	59°06'	Zarrei *et al*., 2009
G. calcicola Zarrei & Wilkin	SW Iran	Iran: Khuzestan, 85 km towards Andimeshk, from Poldokhar	A-SW	PLA-2	pla-5	Zarrei & Kamrani 35194 (TUH) (Kew 23155)	EU939255	EU912286	EU912057	33°05'	48°28'	*Zarrei et al., 2009*
G. caroli-kochii Grossh.	South-west Asia*	Azerbaijan	A-SW	PLA-3	pla-6	TUH-E BOT.EXP. 35715 (TUH) (Kew 23139)	EU939228	EU912256	EU912029	37°09'	44°52'	*Zarrei et al., 2009*
G cf. caroli-kochii		Azerbaijan	A-SW	PLA-15	pla-14	GAT43975	LT985615	LT985861	LT985631	40°50'29''	48°52'14''	this study
G. commutata K.Koch	South-west Asia*	Azerbaijan: Ardabil, mts. Around Kalibar	A-SW	PLA-4	-	Zarrei 876 (K, LE, TUH) (Kew 23336)	EU939237	EU912266	-	38°51'	47°02'	*Zarrei et al., 2009*
		Israel: Mt. Hermon	M	PLA-5	-	HAL 116004	LT985616	LT985862	-	33° 13'	35°41`	this study
		Israel: Alon Ha'Galil	M	PLA-5	-	Dafni s.n. (Kew 23410)	EU939296	EU912329	-	32°45'	35°13'	*Zarrei et al., 2009*
G. divaricata Regel	Pamir-Alai, South-west Asian, Western Tien Shan*	Kazakhstan: Kapchagay	A-SW	PLA-6	pla-7	HAL 118078	LT985617	LT985863	LN874795	43°44'28''	76°01'20''	Peterson et al., 2016, this study
		Kazakhstan: Kapchagay	A-SW	PLA-6	pla-7	HAL 118079	LT985618	LT985864	LN874796	43°44'28''	76°01'20''	Peterson et al., 2016, this study
		Kazakhstan: Kapchagay	A-SW	PLA-6	pla-7	146/10DNALevichev (LE)	LT985619	LT985865	LN874797	43°51'	77°03'	Peterson et al., 2016, this study
G. *eleonorae* Levichev	South-west Asia*	Russia: Region Stawropol, Swetlograd	ES	PLA-1	pla-8	HAL 101838	AJ973163	AJ970179	AM287274	45°19'	42°51'	Peterson et al., 2008
G. graminifolia Vved.	Pamir-Alai, South-west Asian, Western Tien Shan*	Kyrgyzstan:	A-SW	PLA-7	pla-9	114/09DNALevichev (LE)	FR690846	FR690251	FR689769	39°35'	69°45`	Peterson et al., 2011
G. helicophylla Levichev	Pamir-Alai, South-west Asia*	Russia: Dagestan	A-SW	PLA-1	pla-10	HAL 103618	AM265590	AM238542	AM422456			Peterson et al., 2008
G. reticulata (Pall.) Schult. et Schult.f.	Europea, South-west Asian*	Armenia: near Erivan	A-SW	PLA-8	-	HAL 103856	AM238528	AM238532	-	40°10'	44° 29'	Peterson et al., 2008
		Israel: Central Negev: from Diamona to the S.W., near Yerocham	M	PLA-8	pla-11	Z 34650	AJ973161	AJ890371	AM087954	30°59'	34°55'	Peterson et al., 2008
		Israel: Southern Negev: vic. Har Ramon	M	PLA-8	-	Z 34651	AM287270	AM265518	-	30°29'	34°37'	Peterson et al., 2008
		Iran: Fars	A-SW	PLA-11	pla-11	Zarrei & Kamrani 35186 (K, TUH) (Kew 23284)	EU939258	EU912289	EU912060	29°42'	51°47'	*Zarrei et al., 2009*
		Iran: Khorassan	A-SW	PLA-9	pla-11	Zarrei & Ajani 832 (IRAN, K, M, TUH) (Kew 23287)	EU939259	EU912290	EU912061	35°35'	59°39'	*Zarrei et al., 2009*
		Iran: Khorassan	A-SW	PLA-9	-	Zarrei & Ajani 832 (IRAN, K, M, TUH) (Kew 23287)	EU939259	EU912290	-	35°35'	59°39'	*Zarrei et al., 2009*
		Iran: Khorassan	A-SW	PLA-10	pla-11	Zarrei & Golzarian 35260 (TUH) (Kew 23150)	EU939254	EU912285	EU912056	35°35'	59°05'	*Zarrei et al., 2009*
		Iran: Khuzestan, 10 km from Poldokhtar towards Andimeshk	A-SW	PLA-11	pla-11	Zarrei & Kamrani 35196 (TUH) (Kew 23174)	EU939256	EU912287	EU912058	33°15'	47°43'	*Zarrei et al., 2009*
		Iran: Province Tehran: Karai rood	A-SW	PLA-8	pla-11	HAL 103616	AJ973162	AJ969121	AM422461			Peterson et al., 2008
		Lebanon: Houwaghir	M	PLA-8	-	PI *s.n.*	LT985680	LT985866	-	34,499478	36,11824	this study
G. cf. *reticulata*	Iran	Iran: NE, Golestan, Golestan NP, hills above Almeh	A-SW	PLA-12	pla-12	Zarrei & Zarre 1032 (K) (Kew 23271)	EU939257	EU912288	EU912059	37°54'	56°38'	*Zarrei et al., 2009*

G. rigida Boiss. & Spruner	Mediterranean; South-west Asia*	Greece: Crete, Kritsa to Katharo high plain	M	PLA-13	-	HAL 117639	LT985715	LT985817	-	35° 9'	25° 38'	this study
		Greece: Crete, Kolkithi peninsula	M	PLA-13	-	HAL 117640	LT985716	LT985818	-			this study
		Greece: Crete, Plakias, Kouroupa	M	PLA-13	pla-13	HAL 112571	LT985719	LT985821	LT985634	35° 13'	24° 26'	this study
		Greece: Crete, Plakias, Siela (Krioneritis)	M	PLA-13	pla-13	HAL 112570	LT985717	LT985819	LT985632	35° 12'	24°22'	this study
		Greece: Crete, Plakias, Siela (Krioneritis)	M	PLA-13	pla-13	HAL 112569	LT985718	LT985820	LT985633	35° 12'	24°22'	this study
G. sarmentosa K.Koch	Europe, South-west Asia*	Russia: Dagestan, Machatschkala	ES	PLA-14	pla-14	HAL 103617	AM265587	AM238537	AM422458	42,98	47,49	Peterson et al., 2008
G. setifolia Baker	Pamir-Alai, South-west Asia*	Iran: Khorasan	A-SW	PLA-16	pla-15	Zarrei & Golzarian 35246	EU939261	EU912292	EU912063	37°32'	56°22'	*Zarrei et al., 2009*
		Iran: Khorassan, Mashhad	A-SW	PLA-20	pla-15	Zarrei & Golzarian 35213	EU939267	EU912298	EU912069	35°24'	59°13'	*Zarrei et al., 2009*
		Iran: Khorassan, Neyshabur, Binalud Mts, Kharr-e bala	A-SW	PLA-16	pla-18	Heydari 30547 (K) (Kew	EU939263	EU912294	EU912065	36°25'	58°51'	*Zarrei et al., 2009*
		Iran: Khorassan, Qaen towards Khaf	A-SW	PLA-17	pla-17	Zarrei & Golzarian 35254 (TUH) (Kew 23175)	EU939262	EU912293	EU912064	33°57'	59°38'	*Zarrei et al., 2009*
		Iran: Semnan, Jabil Pass, Toroud twd Soheil, Biarjomand Pass	A-SW	PLA-12	pla-20	Mohammadi 35198 (K,TUH) (Kew 23285)	EU939265	EU912296	EU912067	35°34'	53°22'	*Zarrei et al., 2009*
		Iran: Tehran, Karaj towards Chalus	A-SW	PLA-16	pla-16	Zarrei 35289 (TUH) (Kew 23149)	EU939253	EU912284	EU912055	36°04'	51°18'	*Zarrei et al., 2009*
		Iran: Tehran, N of Tehran, Tochal Mts	A-SW	PLA-19	pla-19	Zarrei & Zarrei 35268 (K,TUH) (Kew 23290)	EU939266	EU912297	EU912068	35°53'	51°25'	*Zarrei et al., 2009*
		Iran: Yazd, 90 km S of Yadz, Kermanshah Pass	A-SW	PLA-18	-	Zarrei 1017 (K) (Kew 23272)	EU939264	EU912295	-	31°13'	55°31'	*Zarrei et al., 2009*
G. taschkentica Levichev	Pamir-Alai, South-west Asia, Western Tien Shan*	Uzbekistan	A-SW	PLA-21	-	HAL 103854	AM265591	AM238536	-	41°17'	69°14'	Peterson et al., 2008
G. ugamica Pavl.	South-west Asia, Western Tien Shan*	Kyrgyzstan: Pereval Ashu-Ter	A-SW	PLA-22	pla-21	HAL 101827	AM265593	AM265516	AM422459	42°27'	79°3'	Peterson et al., 2008
G. vegeta Vved.	Pamir-Alai, South-west Asia*	Iran: Prov. Gorgan, Agh-Ghala, Seghar Tappeh	A-SW	PLA-4	pla-22	Shafii 475 (Shahed University Herbarium) (Kew 23181)	EU939274	EU912305	EU912076	37°01'	54°46'	*Zarrei et al., 2009*
		Uzbekistan	A-SW	PLA-23	pla-23	HAL 103691	AM238520	AM180468	AM287275	39°17'	66°57'	Peterson et al., 2008
Plecostigma (Turcz.) Engler												
G. afghanica A.Terracc.	Pamir-Alai; South-west Asian, Western Tien Shan*	Iran: Golestan, Kalaleh towards Ashkhaneh, 5 km after Robate	A-SW	PLE-2	ple-2	Zarrei & Golzarian 35207 (K,TUH) (Kew 23283)	EU939223	EU912249	EU912023	37°41'	56°16'	*Zarrei et al., 2009*
		Iran: Khorassan, Baigiran towards Quchan, 4 km after Baigiran	A-SW	PLE-2	ple-2	Zarrei & Golzarian 35257 (TUH) (Kew 23156)	EU939221	EU912247	EU912021	37°35'	58°26'	*Zarrei et al., 2009*
		Iran: Khorassan, 65 km to Ghaen from Gonabad, 10 km before Khezry	A-SW	PLE-2	ple-2	Zarrei & Golzarian 35223 (K,TUH) (Kew 23282)	EU939222	EU912248	EU912022	34°11'	58°59'	*Zarrei et al., 2009*
		Iran: Province Khorasan, 5 km after Fariman to Aghar villade	A-SW	PLE-1	ple-1	HAL 101785	AJ973160	AJ890373	AM087953	35°44'50''	59°55'15''	Peterson et al., 2008
G. altaica Schischk & Sumnev.	East Asian; Siberian; South-west Asian*	Kazakhstan: Karabirok	A-SW	PLE-3	ple-3	HAL 101786	AJ973159	AJ890374	AM162670	48°00'	84°40'	Peterson et al., 2008
G. chlorantha (M.Bieb.) Schultes & Schultes	European, South-west Asian*	Iran: Ardabil, 10 km to Kaleibar from Ahar	A-SW	PLE-8	ple-6	Zarrei 872 (K, TUH) (Kew 23269)	EU939233	EU912261	EU912034	38°51'	47°07'	*Zarrei et al., 2009*
		Iran: Hamadan, 36 km to Avaj from Takestan	A-SW	PLE-7	ple-5	Zarrei & Zarre 778 (K, TUH) (Kew 23268)	EU939232	EU912260	EU912033	35°48'	49°25'	*Zarrei et al., 2009*
		Iran: Khuzestan	A-SW	PLE-6	ple-4	Zarrei & Kamrani 35192 (TUH) (Kew 23138)	EU939230	EU912258	EU912031	32°29'	48°38'	*Zarrei et al., 2009*
		Iran: Khuzestan, 10 km from Poldokhtar towards Andimeshk	A-SW	PLE-6	ple-4	Zarrei & Kamrani 35195 (TUH) (Kew 23167)	EU939231	EU912259	EU912032	33°00'	47°76'	*Zarrei et al., 2009*
		Israel: Betlemme	M	PLE-5	-	PI *s.n.*	LT985684	LT985870	-	31.77	35.20	this study
		Israel: Gerusalemme	M	PLE-5	-	PI *s.n.*	LT985683	LT985869	-	31.77	35.22	this study
		Israel: Mt. Ghilboa (NE Israel)	M	PLE-4	-	HAL 116001	LT985681	LT985867	-	32.26	35.24	this study
		Israel: Mt. Ghilboa (NE Israel)	M	PLE-4	-	HAL 116002	LT985682	LT985868	-	32.26	35.24	this study
G. iranica M.Zarrei & Zarre	Iran*	Iran: Gorgan	A-SW	PLE-9	ple-7	Zarrei & Golzarian 35210 (TUH) (Kew 23147)	EU939248	EU912279	EU912050	37°39'	56°04'	*Zarrei et al., 2009*
		Iran: Mazandaran	A-SW	-	ple-7	Zarrei & Golzarian 35251 (TUH) (Kew 23173)	EU939249	EU912280	EU912051	36°13'	52°32'	*Zarrei et al., 2009*
G. jensii Levichev & Schnittler	N China	China: Xinjiang,Urumqi, hills near cemetery	A-E	PLE-10	ple-8	HAL 108482	FR691025	FR690256	FR690101	43°43′53″	87°35′46"	Peterson et al., 2011
		China: Xinjiang,Urumqi, hills near cemetery	A-E	PLE-10	ple-8	HAL 108483	FR691026	FR690257	FR690102	43°43′53″	87°35′46"	Peterson et al., 2011
		China: Xinjiang,Urumqi,Yamalik Hill Forest Park	A-E	PLE-10	ple-8	HAL 108480	FR691027	FR690258	FR690103	43°47′26″	87°34′21″	Peterson et al., 2011
G. neo-popovii Golosk	East Asian; South-west Asian; Western Tien Shan*	Kazakhstan: Eastern, Za-Iliskii Altai	A-SW	PLE-11	ple-9	120/09DNALevichev (LE)	FR690849	FR690254	FR689770	43°09'	77°04'	Peterson et al., 2011
G. olgae Regel	Pamir-Alai, South-west Asian, Western Tien Shan*	Uzbekistan: Samarkand	A-SW	PLE-12	-	HAL 103859	AM085144	AM161465	-	39°37'	66°58'	Peterson et al., 2008
G. pauciflora Turcz. ex Ledeb.	East Asian, Siberian*	Mongolia: Ulan Bator	A-E	PLE-13	ple-10	HAL 070423	AJ973168	AJ890372	AM409330	47°53'	106°54'	Peterson et al., 2008
		Russia: District Krasnojarsk: Sajano-Schuschensker Sapowednik	ES	PLE-13	ple-10	HAL 103857	AM287266	AM161468	AM493952	52°49'	91°22'	Peterson et al., 2008
G. uliginosa Siehe & Pascher	East Asian, Siberian*	Iran: Ardabil	A-SW	PLE-14	ple-11	TUH-E BOT.EXP. 35304 (TUH) (Kew 23153)	EU939281	EU912312	EU912089	38°12'	47°52'	*Zarrei et al., 2009*
		Iraq: Kandil Range (NE of Ranial)	A-SW	PLE-15	-	Rawi & Serhang 18286 (K) (Kew 23277)	EU939282	EU912313	-	36°32'	45°0'	*Zarrei et al., 2009*
G. wendelboi Rechinger	South-west Asian*	Iran: E Alborz	A-SW	PLE-16	ple-12	Matin 35605 (IRAN) (Kew 23183)	EU939284	EU912315	EU912091	35,996	50,93	*Zarrei et al., 2009*
Spathaceae Levichev												
G. spathacea (Hayne) Salisb.	Europe, Caucasus*	Belgium: Raspaillebos, Geraardsbergen	ES	SPA-1	spa-1	HAL 110368	FR874122	LT985871	FR874113	50°46'	3°52'	Pfeiffer et al., 2012; this study
		Germany, Bavaria, Oberpfalz, Berngau "Obersau"	ES	SPA-1	spa-1	HAL 108202	FR691830	LT985872	FR691811	49°15'	11°23'	Pfeiffer et al., 2012; this study
		Germany Mecklenburg-Western Pomerania, Altkalener Wald	ES	SPA-1	spa-1	HAL 108198	FR691834	LT985873	FR691815	53°53'	12°44'	Pfeiffer et al., 2012; this study
		Germany: Saxony-Anhalt, Altmark	ES	SPA-2	spa-2	HAL 095844	AJ416369	AJ419166	AJ427541			Peterson et al., 2004
		Italy: Emilia Romagna (Serramazzoni)	M	SPA-1	spa-1	CLU 17518	AM409347	AM283108	AM422465	44°25'	10°47'	Peterson et al., 2008
		Netherlands: Denekamp	ES	SPA-1	spa-3	HAL 108188	FR691844	LT985874	FR691825	52°22'	7°00'	Pfeiffer et al., 2012; this study
		Russia: Kaliningrad	ES	SPA-1	spa-1	HAL 101829	AJ973174	AJ969126	AM422457	54°42'	20°27'	Peterson et al., 2008
		Russia: Caucasus Mts, near Adler	A-SW	SPA-1	spa-1	HAL 112557	FR691826	LT985875	FR691808	43°30'	40°2'	Pfeiffer et al., 2012; this study

***Stipitatae* (Pascher) Davlian.**												
G. caelestis Levichev	South-west Asia,Western Tien Shan*	Kyrgyzstan: Atschik Tasch	A-SW	STI-1	sti-1	HAL 101790	AJ973165	AJ969118	AM180456	39°21'	72°52'	Peterson et al., 2008
G. capillifolia Vved.	Pamir-Alai,South-west Asia,Western Tien Shan*	Uzbekistan: Minora	A-SW	STI-2	sti-2	HAL 101791	AJ973171	AJ970177	AM087951			Peterson et al., 2008
G. chomutovae (Pascher) Pascher	Pamir-Alai,South-west Asia,Western Tien Shan*	Afghanistan: Kabul, Tob-Berg	A-SW	STI-3	-	HAL 111691	LT985685	LT985812	-	34°53'	69°17'	ths study
		Iran	A-SW	STI-3	-	HAL 116008	LT985686	LT985813	-	37.27082	58.35078	this study
		Iran: Khorassan	A-SW	STI-4	sti-3	Zarrei & Golzarian 35214 (TUH) (Kew 23146)	EU939234	EU912263	EU912036	35°35'	59°05'	*Zarrei et al., 2009*
		Iran: Tehran, Talegghan, mts around Shahrak	A-SW	STI-4	sti-4	Zarrei 35814 (TUH) (Kew 23168)	EU939235	EU912264	EU912037	35°60'	51°42'	*Zarrei et al., 2009*
		Kyrgyzstan: Talasskaya Oblast	A-SW	STI-3	sti-3	HAL 101793	AM049262	AJ970176	AM087950	42°17'	72°31'	Peterson et al., 2008
G. dschungarica Regel	Pamir-Alai,South-west Asia,Western Tien Shan*	Iran: Mazandaran, Tehran to Polur, c. 500 m after Emamzade-Hashem	A-SW	STI-6	sti-6	Zarrei 35815 (TUH) (Kew 23143)	EU939240	EU912270	EU912043	35°46'	52°2'	*Zarrei et al., 2009*
		Iran: Tehran	A-SW	STI-6	sti-6	Zarrei 35290 (TUH) (Kew 23170)	EU939241	EU912271	EU912044	36°04'	51°18'	*Zarrei et al., 2009*
		Kyrgyzstan: Pamirsky National Park, Tadschikistan Atschik Tasch	A-SW	STI-5	sti-5	HAL 101802	AJ973164	AJ970175	AM087952	39°45'	73°28'	Peterson et al., 2008
		Turkmenistan: Zentraler Kopet Dag Xeupachg	A-SW	STI-5	-	GAT13178	LT985687	LT985814	-	38°04'	57°22'	this study
G. libanotica (Hochst.) Greuter	Meditereanean, South-west Asia*	Israel: Mt. Hermon	M	STI-7	-	Townsend 74/38 (K) (Kew 23338)	EU939286	EU912317	-	33°25`	35°51`	*Zarrei et al. 2009*
		Lebanon: Jebel Sannin	M	STI-7	-	HAL 116005	LT985688	LT985815	-	33°57'0"	35°52' 26.695"	this study
G. ova Stapf	Pamir-Alai,South-west Asia,Western Tien Shan*	Afghanistan: near Kabul	A-SW	STI-8	-	HAL 11508	LT985689	LT985816	-	34°52'	69°17'	this study
		China: Xinjiang, Urumqi, Yamalik Hill Forest Park	A-E	STI-9	sti-9	HAL 107951	FR690838	FR690243	FR689762	43°47′44″	87°34′29″	Peterson et al., 2011
		China: Xinjiang, Urumqi, Yamalik Hill Forest Park	A-E	STI-9	sti-9	HAL 107950	FR690839	FR690244	FR689763	43°47′44″	87°34′29″	Peterson et al., 2011
		Iran: Province Khorasan: Bajgiran rood	A-SW	STI-9	sti-7	HAL 101834	AM238526	AM180465	AM287276			Peterson et al., 2008
		Iran: Province Golestan: Golestan National Park, Dashte Sharlegh	A-SW	STI-9	sti-8	HAL 101833	AM265588	AM180466	AM287277	37°33`	56°23`	Peterson et al., 2008
G. pseudominutiflora Levichev	South-west Asia,Western Tien Shan*	Kazakhstan	A-SW	STI-1	sti-1	HAL 103851	AM238523	AM238533	AM493957			Peterson et al., 2008
G. stipitata Merckl. ex Bunge	Pamir-Alai,South-west Asia,Western Tien Shan*	Iran: Fars, Saadat-Shahr twds. Abadeh	A-SW	STI-11	sti-12	Zarrei & Kamrani 35197 (TUH) (Kew 23177)	EU939270	EU912301	EU912072	30°05'	53°12'	*Zarrei et al., 2009*
		Iran: Khorassan, Robat-e Sang, twds. Kadkan	A-SW	STI-11	sti-13	Zarrei & Golzarian 35215 (K, TUH) (Kew 23293)	EU939271	EU912302	EU912073	35°35'	59°05'	*Zarrei et al., 2009*
		Iran: Province Khorasan: 7 km from Bojnourd to Shirvan	A-SW	STI-10	sti-10	HAL 101828	AM265594	AM265519	AM409336	37°28'46''	57°24'21''	Peterson et al., 2008
		Iran: Tehran, 43 km towards Damavand from Firouzkuh, between Firouzkuh and Tehran	A-SW	STI-11	sti-11	Zarre & Zarrei 35297 (TUH) (Kew 23154)	EU939268	EU912299	EU912070	36°05'	52°42'	*Zarrei et al., 2009*
		Iran: Tehran, Abyek twds Talegan, 2 km after Zeyaran twds Samgh-Abad	A-SW	STI-11	-	Zarrei & Kamrani 35275 (TUH) (Kew 23176)	EU939269	EU912300	-	36.121	50.55	*Zarrei et al., 2009*
G. ×turanica Levichev	South-west Asia,Western Tien Shan*	Iran: Province Kemran	A-SW	STI-12	sti-14	HAL 103852	AM238529	AM238540	AM493956	28°53'01''	56°23'48''	Peterson et al., 2008
***Triflorae* Peruzzi**												
G. triflora (Ledeb.) Schult. & Schult.f.	East Asian*	Russia: Amur Oblast, Khinganskiy Zapovednik	A-E	TRIF	trif	HAL 101788	AM049261	AJ890377	AM162674	49°6'	130°23'	Peterson et al., 2008
***Tricholloydia* (Engl.) Zarrei & Wilkin**												
Gagea flavonutans (H.Hara) Zarrei & Wilkin	S Xizang [Bhutan, Nepal, Sikkim][##]	India: Sikkim, North District: Tembawa Chhu valley, E of Yume Samdong	A-H	TRIL-1	-	EEN 422 772*3 E	AM493967	AM493959	-	27°54'	88°43'	Peterson et al., 2008
Gagea oxycarpa (Franch.) Zarrei & Wilkin	S Gansu, Sichan, Xizang, Yunnan[##]	China: Yunnan, Gaoligong Shan Region	A-H	TRIL-2	-	GSE 12650 772*8 (E)	AM493968	AM493960	-	27°41'46''	98°27'19''	Peterson et al., 2008
		China: Yunnan, Thongdian, Little Zhongdian, Tianchi Lake	A-H	TRIL-3	-	ACE 137 (K) (Kew 23342)	EU939289	EU912322	-	27°37'83''	99°37'27''	*Zarrei et al., 2009*
**** G. sect. not determined***												
Lloydia yunnannsis Franch. (*"yun"*)	Kashmir, India, Bhutan, Sikkim, China, W Sichuan, Yunnan[##]	China: Yunnan, Yulong Snow Mt.	A-H	*yun* sect*	-	Luo, Yi-bo 64 (K) (Kew 23337)	EU939287	EU912318	-	27°09'	100°04'	*Zarrei et al., 2009*
		India, Sikkim, West District, between Dzongri Pass and Dzongri	A-H	*yun* sect*	-	ESIK 406 772*4 (E)	AM493964	AM493958	-	27°29'	88°09'	Peterson et al., 2008
Lloydia tibetica Baker ex Oliv. (*"tib"*)	Gansu, Hebei, Hubei, Shaanxi, Shanxi, Sichuan, Xizang [Nepal][##]		A-H	-	*tib* sect**	H 12014	-	-	KP712029*			*Yang,L.Q., He,X.J. and Zhou,S.D.unpubl.*
**OUTGRPOUP**												
**Tribe Tulipeae Duby**												
Amana edulis (Miq.) Honda									KP712031*			
Amana erythronioides (Baker) D.Y.Tan and D.Y.Hong							EU939293*	KY401424*				
Erythronium californicum Purdy									AF485292*			
Erythronium japonicum Decne							HE649688	HE649786				
Tulipa cretica Boiss. & Heldr.							AM049257	AJ810118	AM180461			
**Tribe Lilieae Lam. & DC.**												
Lilium candidum L.							AM049257	AJ810118	AM292424*			

Notes:

[a]Taxononic remarks: New taxonomic conclusions are out of the scope of this paper.

[b]Source of information: *Levichev & Jezniakowsky, 2008 (Historia Gagearum); **Tison et al., 2013; ***Meusel et al., 1965; [#]Ali & Levichev, 2007 (Flora of Pakistan), [##]Xinqi & Turland, 2000 (Flora of China)

[c]In the case of lacking detailed collection data, coordinates of the given region/area were estimated (see Material & Methods). Sample information of Zarrei et al., 2009 were used from The Royal Botanic Gardens, Kew, DNA Bank,http://apps.kew.org/dnabank/ (accessed on 18 August 2009). [d]Sequences from other authors are marked in italics.

Figure S1. BEAST-derived maximum clade credibility (MCC) chronogram of *Gagea* (plus outgroups) based on rITS sequences (CAL1: dating according divergence time of genera of the tribe Tulipeae provided by Givnish et al., 2016). Blue bars indicate 95% highest posterior density (HPD) intervals around mean node ages (in million years ago, Ma). The posterior probabilities (>0.40) for the clades are given above the nodes. For infrageneric classification of *Gagea* shown on the right side see Table S1.

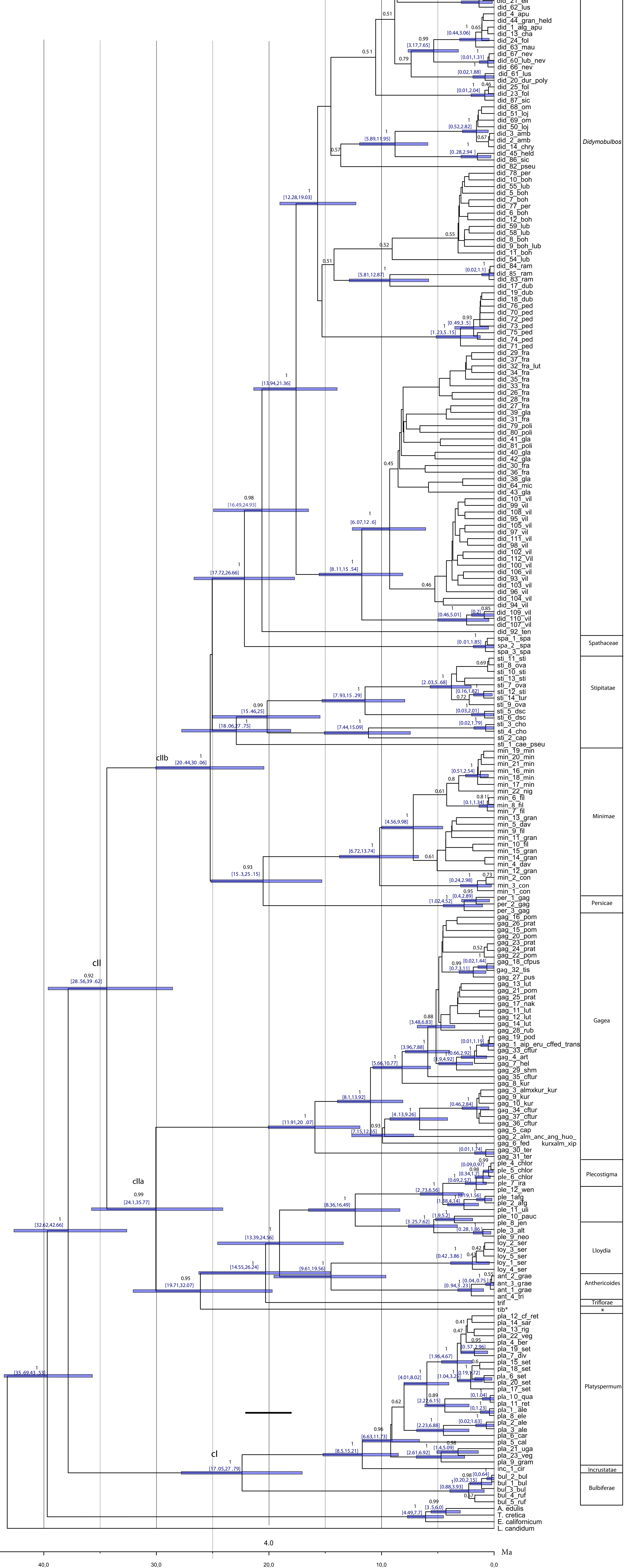

Figure S2. BEAST-derived maximum clade credibility (MCC) chronogram of *Gagea* (plus outgroups) based on nrITS sequences (CAL2: dating according crown age of the genus *Gagea* provided by KIM & KIM, 2018). Blue bars indicate 95% highest posterior density (HPD) intervals around mean node ages (in million years ago, Ma). The posterior probabilities (>0.40) for the clades are given above the nodes. For infrageneric classification of *Gagea* shown on the right side see Table S1.

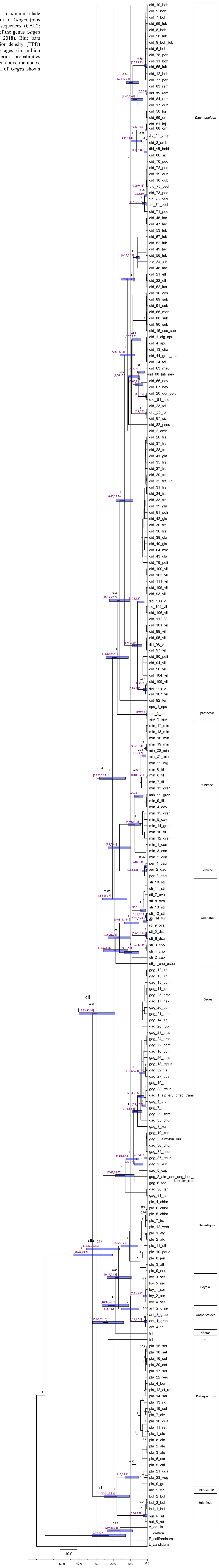

Figure S3. Phylogenetic tree inferred by Bayesian analyses (BA; general time reversible with gamma rates, 5 million cycles) of the combined data set of internal transcribed spacer (ITS) and cpDNA (*psb*A-*trn*H IGS + *trn*L-*trn*F IGS) sequences (265; ITS type_cpDNA haplotype_*taxon*). The two major clades are marked as cI and cII. A major lineage which is not congruent with the ITS tree (Fig. 3) is marked with an apostrophe. For infrageneric classification of *Gagea* shown on the right side see Table S1.

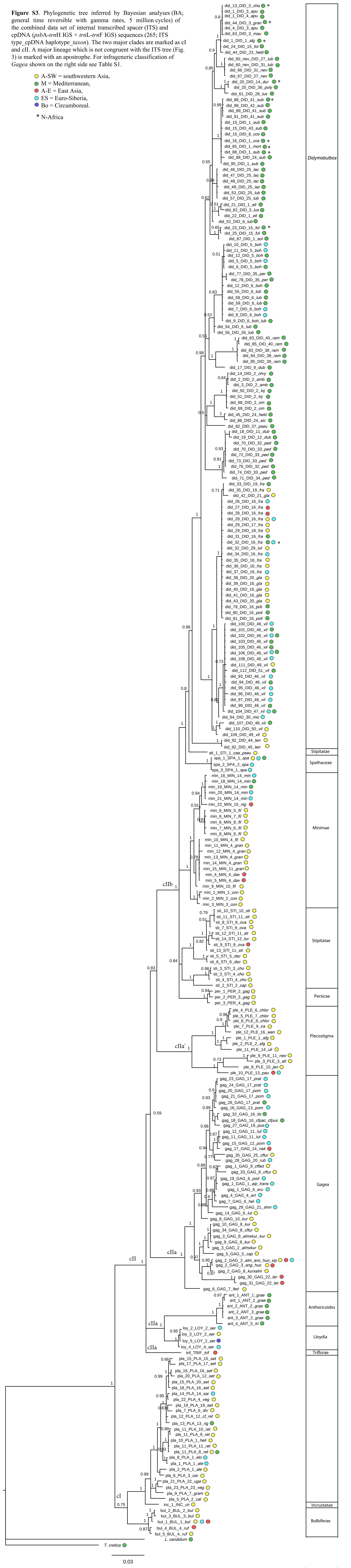

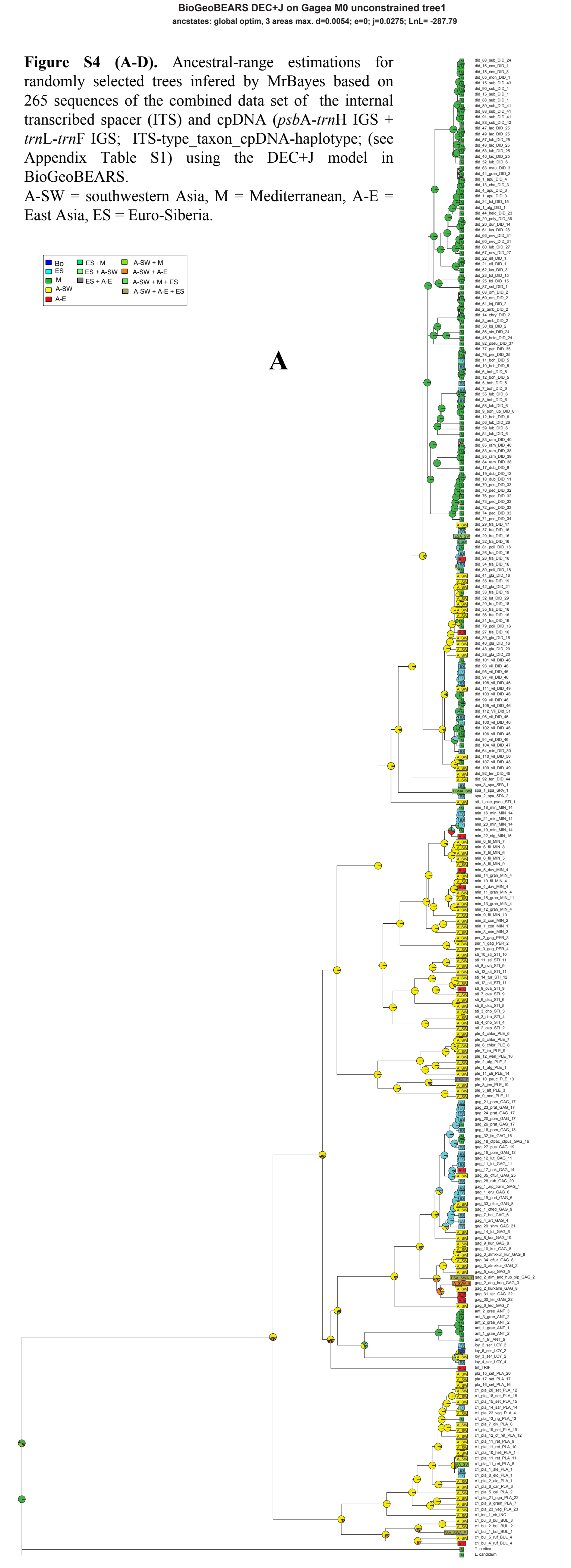

Figure S4 (A-D). Ancestral-range estimations for randomly selected trees infered by MrBayes based on 265 sequences of the combined data set of the internal transcribed spacer (ITS) and cpDNA (*psb*A-*trn*H IGS + *trn*L-*trn*F IGS; ITS-type_taxon_cpDNA-haplotype; (see Appendix Table S1) using the DEC+J model in BioGeoBEARS.
A-SW = southwestern Asia, M = Mediterranean, A-E = East Asia, ES = Euro-Siberia.

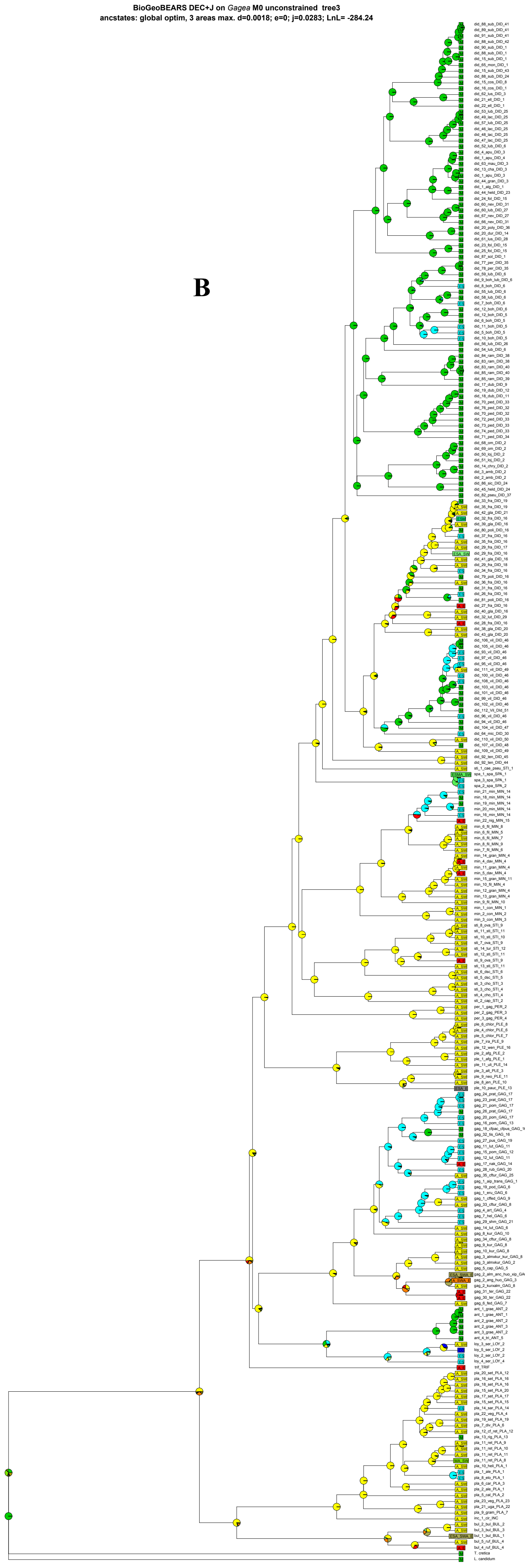
BioGeoBEARS DEC+J on Gagea M0 unconstrained tree3
ancstates: global optim, 3 areas max. d=0.0018; e=0; j=0.0283; LnL= -284.24
B

BioGeoBEARS DEC+J on Gagea M0 unconstrained tree19
ancstates: global optim, 3 areas max. d=0.0091; e=0; j=0.0279; LnL= -294.33

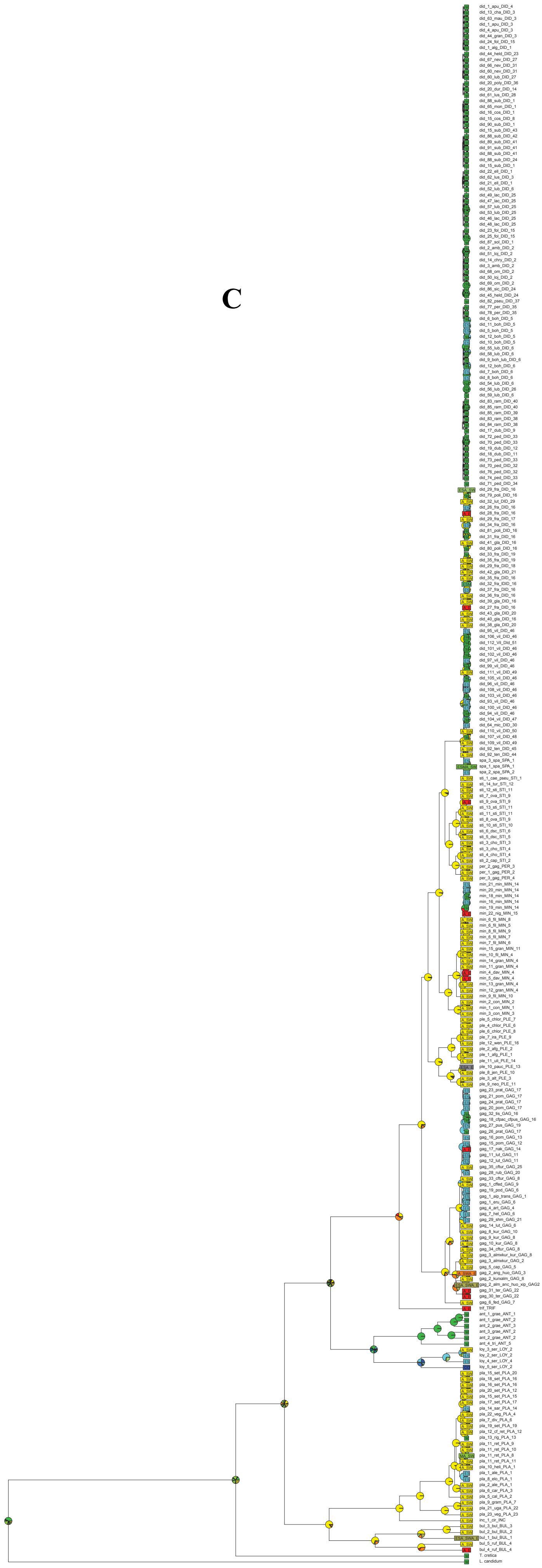

BioGeoBEARS DEC+J on Gagea M0 unconstrained tree11
ancstates: global optim, 3 areas max. d=0.0069; e=0; j=0.0264; LnL= -292.18

D

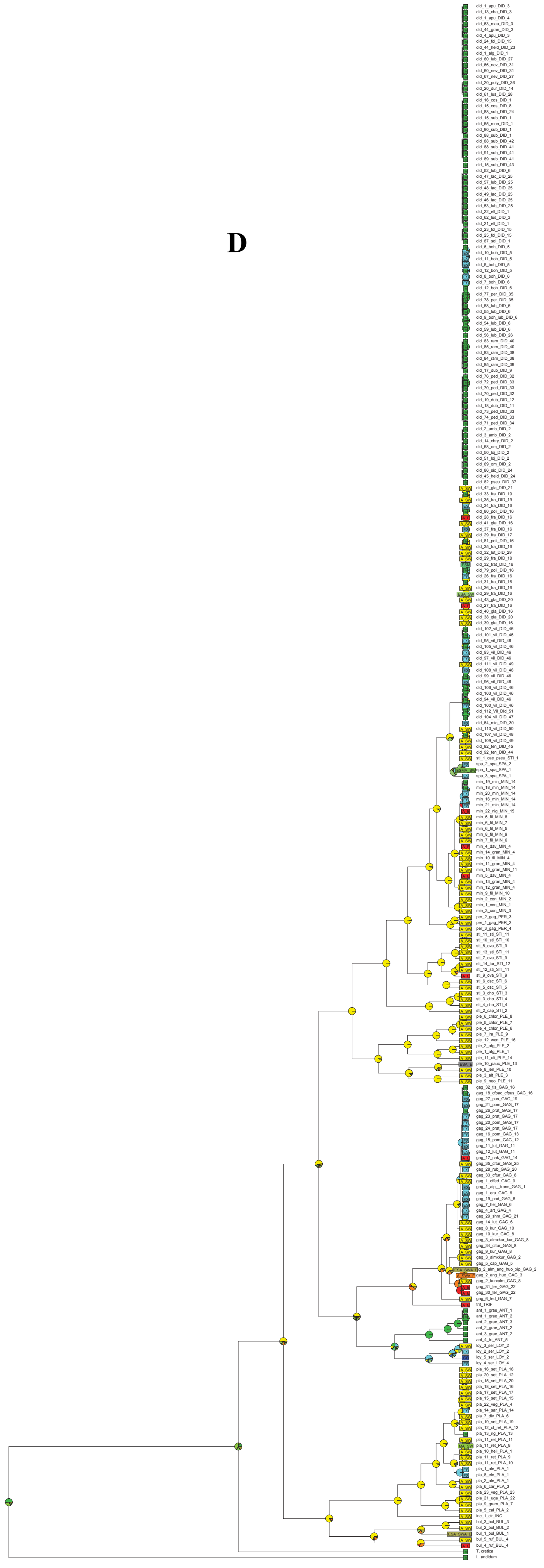

Supplement: Supplementary file 1 [file ECE3-9-5870-s001.pdf]
